# Lipid droplet degradation by autophagy connects mitochondria metabolism to Prox1-driven expression of lymphatic genes and lymphangiogenesis

**DOI:** 10.1038/s41467-022-30490-6

**Published:** 2022-05-19

**Authors:** Odeta Meçe, Diede Houbaert, Maria-Livia Sassano, Tania Durré, Hannelore Maes, Marco Schaaf, Sanket More, Maarten Ganne, Melissa García-Caballero, Mila Borri, Jelle Verhoeven, Madhur Agrawal, Kathryn Jacobs, Gabriele Bergers, Silvia Blacher, Bart Ghesquière, Mieke Dewerchin, Johan V. Swinnen, Stefan Vinckier, María S. Soengas, Peter Carmeliet, Agnès Noël, Patrizia Agostinis

**Affiliations:** 1 Cell Death Research and Therapy Group, Department of Cellular and Molecular Medicine, KU Leuven, Herestraat 49, 3000 Leuven, Belgium; 2 VIB Center for Cancer Biology Research, 3000 Leuven, Belgium; 3 Laboratory of Tumor and Development Biology, GIGA (GIGA-Cancer), Liege University, B23, Avenue Hippocrate 13, 4000 Liege, Belgium; 4 Laboratory of Angiogenesis and Vascular Metabolism, VIB Center for Cancer Biology, VIB, Leuven, Belgium; 5 Laboratory of Angiogenesis and Vascular Metabolism, Department of Oncology, Leuven Cancer Institute, KU Leuven, Leuven, Belgium; 6 Laboratory for Tumor Microenvironment and Therapeutic Resistance, Department of Oncology, KU Leuven, Leuven, Belgium; 7 Laboratory for Tumor Microenvironment and Therapeutic Resistance VIB Center for Cancer Biology, VIB, Leuven, Belgium; 8 Metabolomics Expertise Center, Department of Oncology, KU Leuven, Leuven, Belgium; 9 Laboratory of Lipid Metabolism and Cancer, Department of Oncology, KU Leuven, Leuven, Belgium; 10 Melanoma Laboratory, Molecular Oncology Programme, Spanish National Cancer Research Centre (CNIO), Madrid, 28029 Spain

**Keywords:** Molecular medicine, Medical research

## Abstract

Autophagy has vasculoprotective roles, but whether and how it regulates lymphatic endothelial cells (LEC) homeostasis and lymphangiogenesis is unknown. Here, we show that genetic deficiency of autophagy in LEC impairs responses to VEGF-C and injury-driven corneal lymphangiogenesis. Autophagy loss in LEC compromises the expression of main effectors of LEC identity, like VEGFR3, affects mitochondrial dynamics and causes an accumulation of lipid droplets (LDs) in vitro and in vivo. When lipophagy is impaired, mitochondrial ATP production, fatty acid oxidation, acetyl-CoA/CoA ratio and expression of lymphangiogenic PROX1 target genes are dwindled. Enforcing mitochondria fusion by silencing dynamin-related-protein 1 (DRP1) in autophagy-deficient LEC fails to restore LDs turnover and lymphatic gene expression, whereas supplementing the fatty acid precursor acetate rescues VEGFR3 levels and signaling, and lymphangiogenesis in LEC-Atg5^−/−^ mice. Our findings reveal that lipophagy in LEC by supporting FAO, preserves a mitochondrial-PROX1 gene expression circuit that safeguards LEC responsiveness to lymphangiogenic mediators and lymphangiogenesis.

## Abstract (web summary)

Autophagy is essential to endothelial cell homeostasis. Here the authors show that lipophagy in LEC supports fatty acid oxidation to sustain a mitochondrial-Prox1 gene expression circuit and promote response of LEC to lymphangiogenic mediators.

## Introduction

Lymphatic vessels are specialized components of the circulation system involved in tissue fluid homeostasis, dietary fat absorption, inflammatory and immune responses^1^. Understanding the mechanisms regulating lymphatic vessel formation, a process known as lymphangiogenesis, is a pressing need since its excessive activation or dysfunction contributes to a variety of disease conditions, including inflammation and cancer metastasis, or lymphedema respectively^1,2^.

During development, lymphatic endothelial cells (LEC) differentiate from embryonic venous endothelial cells, through a signaling pathway driven by the programmed induction of the transcription factor Prospero Homeobox 1 (PROX1), which increases the expression of the VEGFR3 receptor^3–5^. VEGFR3 receptors are sensors of the pro-lymphangiogenic ligands VEGF-C or VEGF-D in the microenvironment, determining the activation and directional migration of LEC^6^. Several studies have disclosed the essential role of the master transcription factor PROX1 for the maintenance of LEC identity^5,7^. However, a full understanding of the molecular mechanisms tuning the PROX1-driven gene expression program that sustains the main LEC effector VEGFR3 and functional lymphangiogenesis in adulthood is still lacking^8^.

Mounting evidence suggests a fundamental role for autophagy, the main lysosomal pathway for intracellular disposal of aberrant or obsolete cellular components and their recycling, in vascular biology^9,10^. Recycling of metabolites through autophagy can either be used for biosynthetic routes, or to support mitochondria metabolism and energy production. Apart from being a general degradative pathway, selective autophagy pathways^11^ guarantee organellar and cellular homeostasis and contribute to redirect metabolite flow, in a cell-type and stress-dependent manner^12^. The vasculoprotective role of autophagy in endothelial cells has been derived mainly from genetic and pharmacological studies in blood vessels^9^. In contrast, whether and how autophagy regulates fundamental aspects of LEC biology and postnatal lymphangiogenesis, remains understudied. Here, we demonstrate that autophagy in LEC through the degradation of LDs acts as a main mechanism for the supply of fatty acids to the mitochondria to foster fatty acid oxidation (FAO) and TCA catabolism, which are required for the maintenance of the PROX1-VEGFR3 feedback loop, and the responses to lymphangiogenic cues in vivo.

## Results

### Autophagy is required for LEC homeostasis and injury-driven lymphangiogenesis

**Fig. 1:**
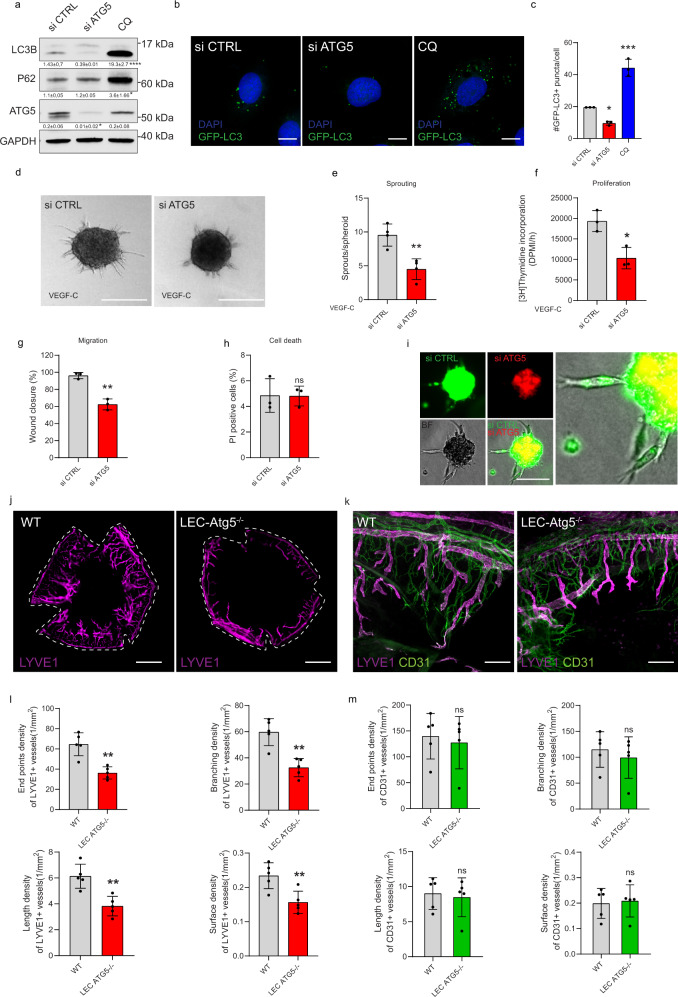
Autophagy is essential for lymphatic endothelial cell homeostasis. **a** Representative blots for indicated proteins of si CTRL, si ATG5, and chloroquine (CQ 25 µM, 48 h) treated LEC. Densitometric quantification is indicated beneath the blots. Mean ± SD, *N* = 3 biological replicates analyzed using one-way ANOVA, with Tukey’s test for multiple comparisons *p < 0.05 and ***p < 0.001 vs si CTRL. Representative images (**b**) and quantification (**c**) of si CTRL, si ATG5 and CQ-treated LEC after GFP-LC3 transfection. Nuclei are stained with DAPI. Scale bars represent 10 µm. Mean ± SD, *N* = 3 biological replicates analyzed by one-way ANOVA, with Tukey’s test for multiple comparisons, *p = 0.02 and ***p = 0.0002 vs si CTRL. Mean represents mean per independent experiment, with a minimum of 19 cells analyzed per condition per independent experiment. Representative images (**d**) and quantification (**e**) of si CTRL and si ATG5 LEC spheroids upon stimulation with VEGF-C (100 ng/mL, 48 h). Scale bar represents 100 µm. Mean ± SD, *N* = 4 independent donors analyzed using unpaired Student’s t test (two-tailed), **p = 0.004. Mean represents mean per independent experiment, with a minimum of 10 spheroids analyzed per condition per independent experiment. **f** [3H]Thymidine incorporation assay into DNA of si CTRL and si ATG5 LEC upon stimulation with VEGF-C. Mean ± SD, *N* = 3 biological replicates analyzed using unpaired Student’s t test (two-tailed), *p = 0.01. **g** Quantification of scratch wound healing for si CTRL and si ATG5 LEC monolayers in the presence of 500 μg/ml Mitomycin C. Mean ± SD, *N* = 3 analyzed using unpaired Student’s t test (two-tailed), ** *p* = 0.001. **h** Flow cytometry analysis of cell death by propidium iodide (PI) of si CTRL and si ATG5 LEC. Mean ± SD, *N* = 3 biological replicates analyzed using unpaired Student’s t test (two-tailed), *p* = 0.97. **i** Representative images of mixed spheroids containing equal amount of fluorescently green labeled si CTRL and red labeled si ATG5 LEC. Scale bar represents 100 µm. **j** Representative immunofluorescent images of whole corneal mounts (dashed lines) of wild type (WT) and LEC-Atg5 knock out mice (LEC-Atg5^−/−^) stained for LYVE1, 8 days post corneal cauterization. Scale bar represents 1 mm. **k** Representative immunofluorescent images of corneal sections dissected from WT and LEC-Atg5^−/−^ mice stained for LYVE1 and CD31, 8 days post corneal cauterization. Scale bars represent 100 µm. **l** Quantification for the number of end points, number of branch points, average of cumulative length, and surface density for LYVE1+ lymphatic vessels. Mean ± SD, *N* = 5 corneas for WT and LEC-Atg5^−/−^ analyzed using unpaired Student’s t test (two-tailed), **p < 0.01. **m** Quantification for the number of end points, number of branch points, average of cumulative length, and surface density for CD31+ blood vessels. Mean ± SD. *N* = 5 corneas for WT and LEC-Atg5^−/−^ analyzed using unpaired Student’s t test (two-tailed), *p* = not significant.

To investigate the overall impact of autophagy in lymphatic EC, we first assessed key autophagy parameters in human dermal LEC (from now on referred to as simply LEC) expressing GFP-LC3, upon autophagy blockade. As expected^13^, silencing the essential autophagy gene ATG5 (si ATG5; resulting in around 80% reduction of ATG5 protein levels) (Fig. 1a), reduced baseline GFP-LC3 punctae and LC3B formation (Fig. 1a–c). Chloroquine (CQ), a lysosomotropic drug that prevents autophagosome-lysosome fusion, increased the accumulation of GFP-LC3 punctae (Fig. 1b, c), LC3B and p62 (Fig. 1a), indicating that LEC display a constitutive autophagic flux, under replete conditions.

We next evaluated the functional consequences of autophagy blockade in LEC. Compared to their si-scrambled controls (si CTRL), ATG5-silenced LEC (si ATG5) displayed a significantly impaired ability to sprout, as detected by a 3D-collagen gel assay recapitulating key features of in vivo sprouting angiogenesis^14^ (Fig. 1d, e), proliferate (Fig. 1f) and migrate (Fig. 1g) in response to VEGF-C, without appreciable differences in cell death (Fig. 1h). We next generated hybrid/chimeric spheroids comprised of equal numbers of autophagy-proficient LEC labeled with a green tracer dye and ATG5-deprived LEC, labeled with a red tracer dye. This analysis showed that while autophagy-proficient green cells competed to migrate and started to sprout, ATG5-compromised red cells remained encapsulated in the spheroid’s core (Fig. 1i), further underscoring their inability to proliferate, migrate, and respond to sprouting cues. LEC sprouting, proliferation, and migration were also repressed by CQ (Supplementary Fig. 1a–d). Similar sprouting defects were observed by the blockade of lysosomal hydrolytic activity with a leupeptin/NH_4_Cl cocktail (Supplementary Fig. 1e, f).

To assess the relevance of autophagy in the response of lymphatic vessels to physiological cues, we generated LEC-specific conditional *Atg5* knockout mice (LEC-*Atg5*^−/−^) by crossing *Agt5*^fl/fl^ mice with *Prox1*-*cre*^*ERT2*^ mice expressing a tamoxifen-inducible Cre recombinase in LEC and used a corneal wound healing model of lymphangiogenesis. In this model, inflammation drives a massive upregulation of proangiogenic factors, which overwhelms the antiangiogenic mechanisms of the cornea and results in a secondary ingrowth of both blood and lymphatic vessels from the limbal area into the corneal center^15^. Tamoxifen induction of Cre recombinase resulted in *Atg5* gene excision in Cre^+^ LEC-*Atg5*^−/−^ mice but not in Cre^−^ Wild Type (WT) mice (Supplementary Fig. 1g), along with a reduction of ATG5 and autophagosomal-bound LC3B immunoreactivity to background levels in the corneal lymphatic vessels, as detected by the specific lymphatic-endothelial cell marker LYVE1 (Supplementary Fig. 1h, i).

To assess the effects of loss of autophagy in vivo, adult 8–12 weeks WT and LEC-*Atg5*^−/−^ mice were subjected to corneal cauterization and lymphangiogenesis was evaluated in the injured corneas 8 days later. Staining of whole mount corneas with the lymphatic marker LYVE1 revealed that LEC-*Atg5*^−/−^ mice displayed significantly reduced lymphangiogenesis (Fig. 1j, k). We then used a computerized quantification method for the determination of morphometric parameters of the corneal structure and neovascularization^16^. Loss of LEC-autophagy in vivo impaired all tested parameters (Fig. 1j, l), indicating that both the lymphangiogenic response (length, density) and the complexity of the vasculature (branching, end points) were significantly affected. Double staining of blood (CD31^+^) and lymphatic (LYVE1^+^) vessels did not reveal defects in blood vessel growth by loss of *Atg5* in LEC (Fig. 1k, m). Similar lymphatic defects, albeit mitigated, were observed with CQ, which toned-down angiogenesis as previously reported^17^ (Supplementary Fig. 1j–m).

Thus, autophagy regulates LEC homeostasis, response to VEGF-C, and injury-induced lymphangiogenesis in vivo.

### Autophagy deficiency impairs the expression of lymphatic markers

**Fig. 2:**
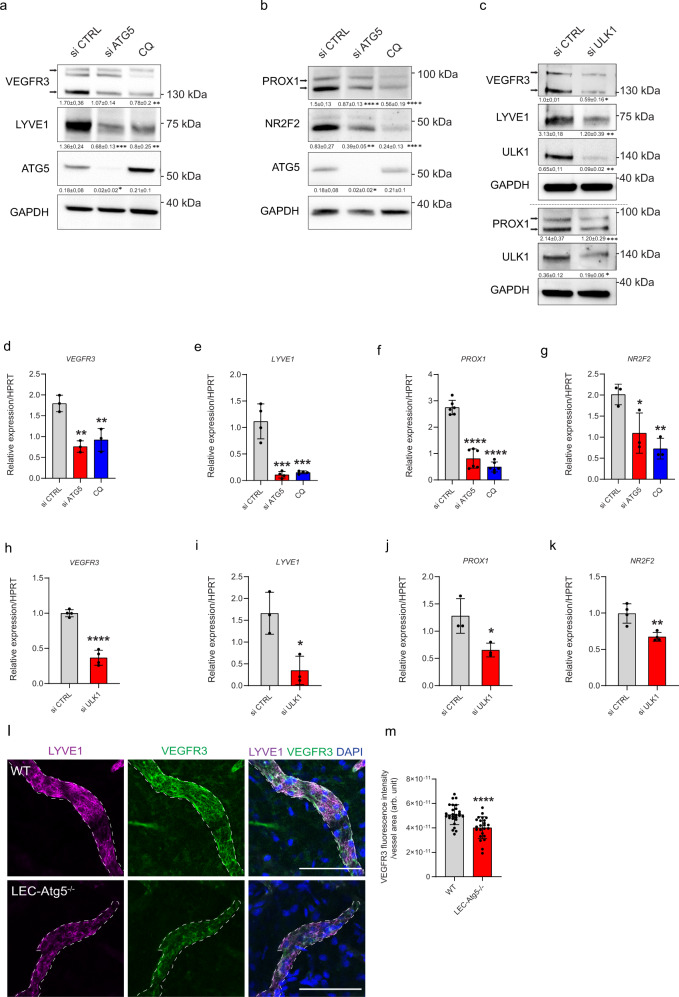
Autophagy deficiency impairs expression of lymphatic markers. **a**, **b** Representative blots for the indicated proteins in si CTRL, si ATG5 or CQ-(25 µM, 48 h) treated LEC. Densitometric quantification is indicated beneath the blots. Mean ± SD, *N* ≥ 3 biological replicates analyzed by one-way ANOVA, with Tukey’s test for multiple comparisons, *p < 0.05, **p < 0.01, ***p < 0.001 and ****p < 0.0001 vs si CTRL. **c** Representative blot for the indicated proteins in si CTRL and si ULK1 LEC. Densitometric quantification is indicated beneath the blots. Mean ± SD, *N* ≥ 3 biological replicates analyzed using unpaired Student’s t test, *p < 0.05, **p < 0.01 and ***p < 0.001. RT-qPCR analysis of si CTRL, si ATG5, or CQ-treated LEC. mRNA expression of VEGFR3, LYVE1, PROX1, and NR2F2 (relative to HPRT). Mean ± SD, *N* = 3 (**d**, **g**), *N* = 4 (**e**) or *N* = 6 (**f**) biological replicates analyzed by one-way ANOVA, with Tukey’s test for multiple comparisons, *p < 0.05, **p < 0.01, ***p < 0.001 and ****p < 0001 vs si CTRL. **h**–**k** RT-qPCR analysis of si CTRL and si ULK1 LEC. mRNA expression of VEGFR3, LYVE1, PROX1, and NR2F2 (relative to HPRT). Mean ± SD, *N* = 3 (**i**, **j**), *N* = 4 (**h**, **k**) biological replicates analyzed by unpaired Student’s t test (two-tailed), *p < 0.05, **p < 0.01, ****p < 0.0001. **l** Representative immunofluorescent images of LYVE1+ lymphatic vessels (dashed lines) from corneal sections dissected from wild type (WT) and LEC-Atg5 knock out mice (LEC-Atg5^−/−^) stained for-VEGFR3. Nuclei are stained with DAPI. Scale bar represents 100 µm. **m** Quantification of VEGFR3 fluorescent intensity (arb. unit, arbitrary unit) per vessel area. Mean ± SD, *N* = 3 corneas with a minimum of 24 images analyzed per condition, analyzed by unpaired Student’s t test (two-tailed), ****p < 0.0001.

We next ascertained whether the observed unresponsiveness of autophagy compromised LEC to VEGF-C could be a consequence of defects in VEGFR3 receptor availability^18^. VEGFR3 protein levels were similarly reduced by the downregulation of ATG5 or CQ treatment (Fig. 2a). Likewise, ATG5 knockdown or CQ attenuated protein levels of other lymphatic markers, including LYVE1, the transcription factors (TFs) Nuclear Receptor 2F2 (NR2F2), and PROX1, which control VEGFR3 signaling and LEC identity^1,19–21^ (Fig. 2a, b). Similarly to CQ, treatment with leupeptin/NH_4_Cl resulted in the downregulation of both VEGFR3 and PROX1 proteins (Supplementary Fig. 2a). Furthermore, genetic interference with Unc-51-like kinase 1 (ULK1) (Fig. 2c), a protein involved in incipient stages of autophagosome biogenesis^22^, or the E1-like activating enzyme ATG7 (Supplementary Fig. 2b) involved in the conjugation of LC3 to PE^22^, phenocopied the inhibitory effects of ATG5 silencing on all tested lymphatic markers. Because compromising the autophagy-lysosomal machinery exerted a downregulation of key LEC markers, we then evaluated the effects of autophagy depletion on their RNA expression. Matching the effects observed on the protein levels, the knockdown of ATG5 (Fig. 2d–g), ULK1 (Fig. 2h–k), ATG7 (Supplementary Fig. 2c–f) or CQ treatment (Fig. 2d–g) in LEC lowered the RNA expression of *VEFGR3* and all tested lymphatic markers, in vitro. Consistent with their curbed response to injury-driven lymphangiogenesis, as compared to WT mice, corneal lymphatic vessels of LEC-*Atg5*^−/−^ mice displayed significantly reduced levels of VEGFR3 (Fig. 2l, m). ATG5 silencing did not significantly alter the expression of *VE-Cadherin, VEGFR2,* or of the blood EC (BEC) markers *CD34, VEGFR1, NRP1,* and *ICAM1* (Supplementary Fig. 2g–l), as reported after ablation of PROX1 expression^7,23,24^, although chemical inhibition of autophagy by CQ showed a trend towards increased expression of *CD34* and *VEGFR1* (Supplementary Fig. 2g–l). This suggests that in cultured LEC transcription is not globally impaired by compromising autophagy and that notwithstanding a milder effect on the upregulation of BEC markers, likely due to incomplete shut down of PROX1 expression (Fig. 2b), autophagy inhibited LEC could still respond to VEGF-A-mediated angiogenesis and lymphangiogenesis. To test this hypothesis, we implanted in the corneas of WT and LEC-*Atg5*^−/−^ mice a pellet containing mouse VEGF-A (mVEGF-A) at the concentration able to mount a lymph/angiogenic response^25^. In contrast to injury-driven lymphangiogenesis, which is promoted by the release of VEGF-C by inflammatory cells^26^, in this micropocket assay an inflammatory response is not a prerequisite for neovascularization^27^ and can therefore be used to determine the direct effect of VEGF-A on lymphangiogenesis^26^. Staining of lymphatic (LYVE1^+^) or blood (CD31^+^) vessels showed that genetic loss of *Atg5* in LEC did not result in significant changes in VEGF-A induced lymph/angiogenesis (Supplementary Fig. 2m–o).

Thus, loss of LEC autophagy impairs primarily the ability of lymphatic vessels to maintain proficient transcription of VEGFR3 and to respond to major lymphangiogenic signals.

### Autophagy regulates lipid droplet homeostasis in LECs

We next wished to clarify which autophagy-regulated mechanism could explain the observed effects of autophagy inhibition on LEC homeostasis and responsiveness to VEGF-C both in vitro and in vivo. We first considered whether compromising autophagy altered mitochondria degradation, a pathway known to contribute to differentiation processes^28^. Silencing ATG5 did not alter the turnover of inner (OPA1) or outer (TOMM20) mitochondrial proteins, mitochondrial network (MitoTracker green), mitochondrial depolarization (TMRM), superoxide levels (MitoSOX), or the co-localization of mitochondria with the lysosomes (Supplementary Fig. 3a, f), when compared to autophagy-proficient LEC. This suggests that the homeostatic unbalance caused by ATG5 deficiency in replete LEC is likely not a direct consequence of obvious defects in canonical mitochondrial degradation pathways.

Because lymphatics are specialized in lipid trafficking and absorption^29^, they express higher levels of fatty acid transporters, such as CD36, as compared to BEC^21^ and (Supplementary Fig. 3g–i).

Since we operate under lipid replete conditions, we then posited that loss of constitutive autophagy could affect lipid trafficking or their storage/turnover. CD36 surface expression was unaffected by the genetic loss of *Atg5* in murine LEC (Supplementary Fig. 3h, i). Likewise, autophagy loss in cultured LEC did not alter the expression of surface CD36 or the cytosolic FA binding protein FABP4 (Supplementary Fig. 3j–m).

FAs are stored as triglycerides (TGs) in lipid droplets (LDs), fat storage organelles composed of a neutral lipid core surrounded by a phospholipid monolayer embedding a variety of proteins, which are dynamically remodeled to support energy demands and FAO^30^.

**Fig. 3:**
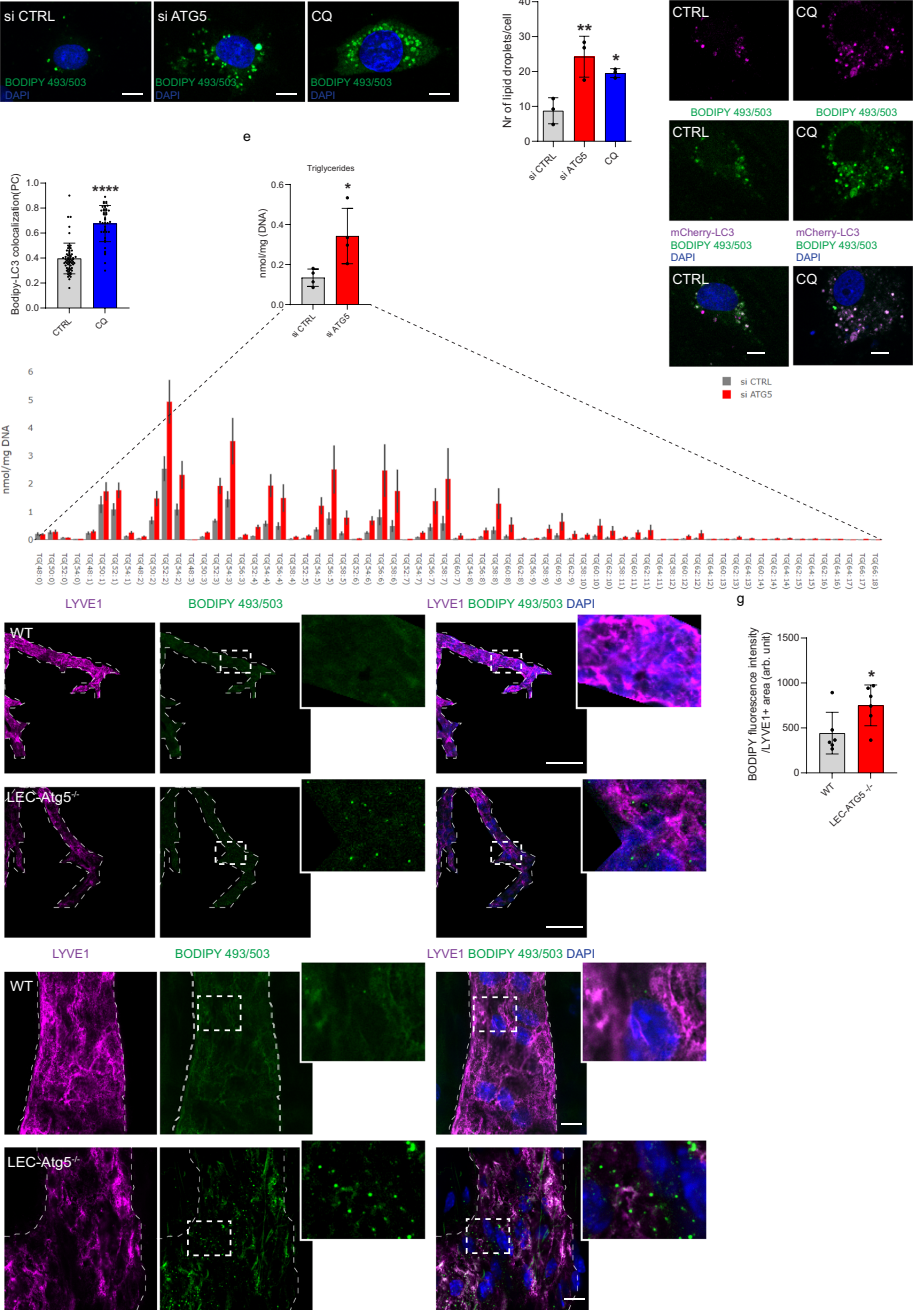
Autophagy regulates lipid droplets homeostasis in lymphatic endothelial cells. **a** Representative images of BODIPY 493/503 staining of lipid droplets in si CTRL, si ATG5, and CQ-(25 µM, 48 h) treated LEC. Nuclei are stained with DAPI. Scale bars represent 10 µm. **b** Quantification of lipid droplet number per cell is shown. Mean ± SD, *N* = 3 biological replicates analyzed by one-way ANOVA, with Tukey’s test for multiple comparisons, *p = 0.04, **p = 0.008 vs si CTRL. Mean represents mean per independent experiment, with a minimum of 52 cells analyzed per condition per independent experiment. **c** Representative images of BODIPY 493/503 staining of lipid droplets in untreated (CTRL) or CQ-treated LEC transfected with a mCherry-LC3 plasmid. Nuclei are stained with DAPI. Scale bars represent 10 µm. **d** Co-localization of BODIPY 493/503 and mcherry-LC3 in untreated (CTRL) or CQ-treated LEC (PC = Pearson coefficient). Mean ± SD, *N* = 3 biological replicates with a minimum of 40 cells per condition in total analyzed by Mann–Whitney test (two-tailed), ****p < 0.0001. **e** Triglycerides bar plots calculated as an average of the lipid subspecies analyzed across si CTRL and si ATG5 LEC. Mean ± SD, *N* = 4 independent LEC donors analyzed by unpaired Student’s t test (two-tailed), *p = 0.03. **f** Representative confocal images of BODIPY 493/503 fluorescence staining (green) of LYVE1+ lymphatic vessels (dashed lines) from corneal sections dissected from wild type (WT) and LEC-Atg5 knock out mice (LEC-Atg5^−/−^). A mask following the shape of the lymphatic vessel (LYVE1+ staining) was applied to isolate the lipid droplets (LDs) signal (BODIPY 493/503) coming from the LYVE1+ lymphatic vessel (dashed lines). Merged images of LYVE1 (magenta), BODIPY 493/503 (green), and DAPI (blue) represent masked images of the three channels. Nuclei are stained with DAPI. Scale bar represents 100 µm. **g** Quantification of BODIPY 493/503 fluorescent intensity calculated per LYVE1+ positive area (representative of lymphatic vessel area) (arb. unit, arbitrary unit). Mean ± SD, *N* = 6 corneas analyzed using unpaired Student’s t test (two-tailed), *p = 0.04. **h** Representative super resolution AiryScan images of BODIPY 493/503 fluorescence staining (green) of LYVE1+ lymphatic vessels (dashed lines) from corneal sections dissected from wild type (WT) and LEC-Atg5 knock out mice (LEC-Atg5^−/−^). Nuclei are stained with DAPI. Scale bars represent 10 µm.

To determine whether autophagy regulates LD homeostasis in LEC, we performed lipid staining with BODIPY 493/503, a fluorescent neutral lipid marker. Inhibiting autophagosome formation by downregulating ATG5 (Fig. 3a, b) or ULK1 (Supplementary Fig. 3n, o), or counteracting the lysosomal degradation of the LD content by CQ (Fig. 3a, b), led to a significant elevation in LD numbers. To further confirm that components of the autophagic machinery associated with LDs, we transiently expressed mCherry-LC3 in control cells stained with BODIPY 493/503. Co-localization analysis revealed that a remarkable fraction of LDs was decorated with LC3 (Fig. 3c, d). BODIPY 493/503/mCherry-LC3 co-localization was significantly boosted in LEC treated with CQ (Fig. 3c, d). Consistent with their increased LD number, quantitative lipidomics analysis revealed significantly higher levels of TGs in ATG5-silenced LEC compared to their si CTRL (Fig. 3e), enriched in several abundant TG species (such as TG 52:2 and TG 54:2) known to accumulate in LDs^31^. CQ treatment increased the cellular content of cholesterol esters (CE) (Supplementary Fig. 3p), likely as a result of the inhibition of lysosomal acid lipase^32^, degrading TGs and CE to FAs and cholesterol, by the CQ-mediated alkalinization of the lysosomes^33^.

We then assessed whether genetic inhibition of ATG5 affects LDs turnover in vivo under conditions driving lymphangiogenesis. To this end, we co-stained lymphatic vessels with LYVE1 and BODIPY 493/503 after corneal injury in WT and LEC-*Atg5*^−/−^ mice (Fig. 3f). Confocal (Fig. 3f, g) and super-resolution imaging (Fig. 3h) analysis showed that endothelial cells lining LYVE1^+^ vessels from LEC-*Atg5*^−/−^ mice displayed increased BODIPY 493/503 fluorescent signal, resulting from the accumulation of LDs, as compared to LEC from WT mice.

Thus, LEC-autophagy regulates LD turnover in steady state conditions in vitro and in response to injury-induced lymphangiogenesis in vivo.

### LEC-autophagy is required for the traffic and release of fatty acid to mitochondria

Ester hydrolysis of TGs liberates free FAs, which can be transported as fatty acyl-CoA into the mitochondria through the outer mitochondrial membrane carnitine-palmitoyl CoA transferase 1 (CPT1A). CPT1A converts long-chain fatty acyl-CoA to the corresponding fatty acyl-carnitines for transport into the mitochondria matrix and thus represents a key step in FAO. Since the genetic loss of the CPT1A isoform compromises in vivo lymphangiogenesis^21^, we reasoned that autophagy-mediated LD degradation might supply FAs to fuel mitochondria FAO and oxidative phosphorylation in LEC^34^.

**Fig. 4:**
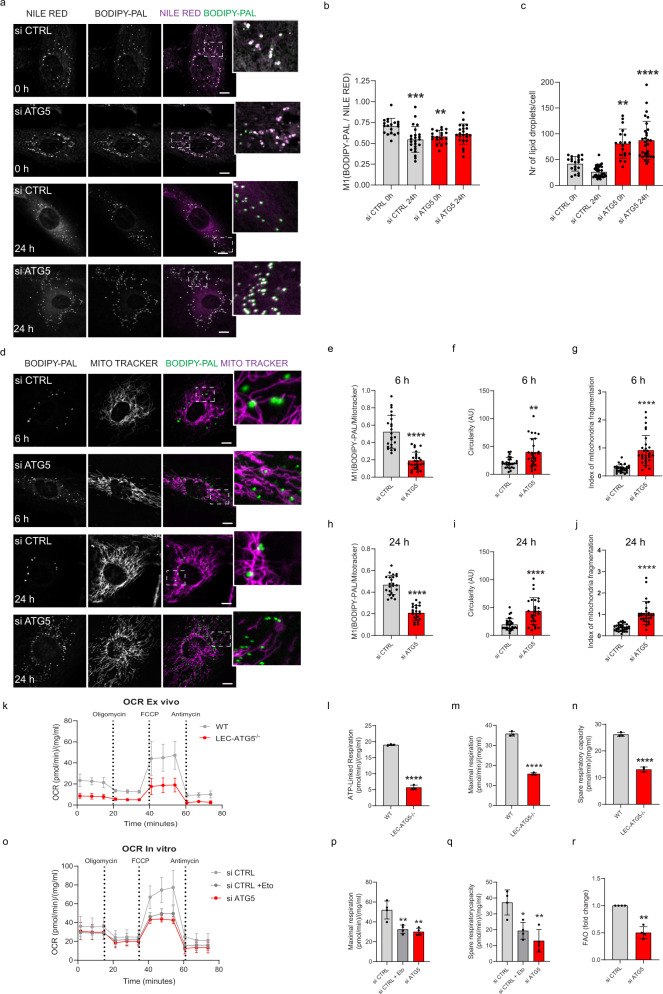
LEC-autophagy is required for the traffic and release of lipids for mitochondria fatty acid oxidation. Representative super resolution AiryScan live images (**a**) and quantification of colocalization of fluorescently labeled (C16:0) palmitate (BODIPY-PAL) in combination with NILE RED (Manders M1 coefficient) (**b**) and quantification of lipid droplets (**c**) in si CTRL and si ATG5 LEC pulsed with BODIPY-PAL (overnight incubation) and imaged at 0 h and after 24 h chase period. Zoomed boxed areas show BODIPY-PAL (green) colocalization with NILE RED-stained lipid droplets (magenta). Mean ± SD, *N* = 3 biological replicates with a minimum of 19 cells per condition in total analyzed using one-way ANOVA with Tukey’s test for multiple comparisons (**b**) and Kruskal–Wallis test with Dunn’s multiple comparisons test (**c**), **p < 0.01, ***p < 0.001 and ****p < 0.0001 vs si CTRL (0 h). Scale bars represent 10 μm. **d**–**j** Representative super resolution AiryScan live images (**d**) of si CTRL and si ATG5 LEC pulsed with BODIPY-PAL (overnight incubation) and imaged after 6 h and 24 h chase period. Mitochondria were labeled with MitoTracker deep red (30 min), before imaging (**e**, **h**), mitochondrial circularity (**f**, **i**), and index of mitochondrial fragmentation (number of mitochondria/ total mitochondrial area) (**g**, **j**). Zoomed boxed areas show colocalization of the mitochondria (magenta) with BODIPY-PAL-signals (green). Mean ± SD, *N* = 3 biological replicates with a minimum of 23 cells per condition in total analyzed using unpaired two-tailed Student’s t test (**e**, **f**, **h**) or two-tailed Mann-Whitney test (**g**, **i**, **j**), **p < 0.01 and ****p < 0.0001 vs si CTRL. Scale bars represent 10 μm. **k** Oxygen consumption rate (OCR) of LEC isolated from WT or LEC-Atg5^−/−^ mice. **l** ATP-linked respiration. **m** Maximal respiration and **n** Spare respiratory capacity from (**k**). Mean ± SD, *N* = 3 biological respiration using unpaired Student’s t test (two-tailed), ****p < 0.0001. **o** OCR of si CTRL or si ATG5 LEC measured in ß-oxidation assay medium. Etomoxir (Eto, 40 μM) was added 15 min prior to baseline measurement in ß-oxidation assay medium. **p** Maximal respiration and **q** Spare respiratory capacity from OCR in (**o**). Mean ± SD, *N* = 4 biological replicates analyzed using one-way ANOVA with Tukey’s test for multiple comparisons, *p < 0.05 and **p < 0.01 vs si CTRL. **r** Fatty acid oxidation (FAO) assay of si CTRL and si ATG5 LEC supplemented with 2 mCi/mL [9,10-3H]-palmitic acid for 18 h. Mean ± SD, *N* = 4 biological replicates analyzed using one sample t test (two-tailed), **p = 0.003.

We then tracked FA incorporation into LDs and their distribution in relation to the mitochondria network by live imaging, using the saturated 16-carbon backbone fluorescent analog BODIPY-palmitate (BODIPY-PAL), which has been used for lipid trafficking studies and shown to incorporate into neutral lipids of LDs^35^. LEC were pulsed overnight with BODIPY-PAL in replete conditions, washed, and incubated in the absence of BODIPY-PAL with Nile Red (to stain LDs) or MitoTracker deep red (to stain the mitochondria network) for 30 min (0 time) or chased further for 6 and 24 h. We then performed co-localization analysis using super-resolution microscopy. Both autophagy-proficient and autophagy-deficient chased cells showed a predominant co-localization of BODIPY-PAL with Nile Red, indicating the incorporation of this FA into LDs (Fig. 4a, b). In si CTRL cells, the BODIPY-PAL/Nile Red co-localization and Nile Red signals gradually decreased within the pulse-chase labeling period, whereas they persisted in the ATG5-defective LEC displaying a significantly higher amount of LDs (Fig. 4a–c). Concomitantly, in si CTRL LEC, the BODIPY-PAL signal co-localized throughout the pulse-chase period with the mitochondrial network (Fig. 4d, e, h), suggesting efficient handover of this FA into the mitochondria. By contrast, autophagy-impaired LEC showed a significantly reduced overlap between BODIPY-PAL with the mitochondria network (Fig. 4d, e, h).

Previous reports indicated the relevance of an interconnected mitochondrial network for the efficient uptake of FAs from LDs^36–39^. Autophagy-depleted LEC exhibited hallmarks of a fragmented mitochondria morphology as indicated by an increased mitochondrial circularity and mitochondrial fragmentation index (number of mitochondria/total mitochondria area) (Fig. 4 d, f, g, i, j), TOMM20 immunocytochemistry (Supplementary Fig. 4a, b) and morphometric analysis by EM (Supplementary Fig. 4c–f). Changes in mitochondria morphology were also observed in CTRL LEC treated with the CPT1A inhibitor etomoxir (Supplementary Fig. 4g, h) as reported previously^33^.

Mitochondria dynamics and shape are vital parameters of the mitochondrial metabolic and bioenergetic status^40^. We, therefore, measured oxygen consumption rates (OCR) in freshly isolated LEC from wild type or LEC-*Atg5* ^−/−^ mice. A notable decrease in both the basal and maximal OCR, which is linked to the ability of EC to proliferate^41^, was observed in LEC isolated from LEC-*Atg5* ^−/−^ mice (Fig. 4k–n). A similar phenotype, although attenuated, was observed in ATG5-depleted LEC (Supplementary Fig. 4i–l) and LEC treated with CQ (Supplementary Fig. 4m–p). Downregulation of ATG5 did not affect extracellular acidification rate (ECAR) under basal conditions and after 2-deoxy-D-glucose (2-DG) treatment (Supplementary Fig. 4q), as also confirmed by MS analysis showing unchanged total abundancies of lactic acid levels (Supplementary Fig. 4r).

We then measured FAO-linked oxygen consumption by evaluating OCR under etomoxir-treated conditions to inhibit CPT1A. Maximal respiration and spare respiratory capacity decreased significantly in autophagy-proficient LEC by the short-term addition of the CPT1 blocker etomoxir, to levels similar to those observed in autophagy-compromised cells (Fig. 4o–q). This suggests that a substantial fraction of OCR in steady state is due to oxidation of FAs^37^ as shown in a previous study, and that the source of the FAs in our model is through lipophagy.

Given that defects in lipophagy compromise FAs delivery to mitochondria (Fig. 4d, e, h), we then evaluated the impact of ATG5 inhibition on mitochondrial FAO, by assessing the rate of [9,10-^3^H]-palmitate oxidation. ATG5 silencing caused a significant reduction (50% decrease) in the ability of LEC to perform FAO (Fig. 4r). Consistent with the role of FAO in the maintenance of redox homeostasis in EC^42^, impairing lipophagy increased the levels of oxidized glutathione GSSG (Supplementary Fig. 4s) and reduced levels of eNOS (Supplementary Fig. 4t, u), which maintains redox homeostasis in EC by regulating levels of reducing equivalents GSH and NADPH^43^.

Hence, lipophagy mobilizes FA through the degradation of LDs thereby sustaining mitochondrial FAO and oxidative phosphorylation in LEC.

### Lipophagy regulates CPT1A expression and acetyl-CoA levels in LEC

CPT1A has been found to be a target of a PROX1-driven feedback loop in LEC^21^, but the homeostatic mechanism that supports this mitochondria-gene expression circuit is unknown. Given that lipophagy supplies FAs to boost FAO and TCA in mitochondria, we then reasoned that loss of LEC-autophagy could result in a concomitant downregulation of CPT1A levels, further impairing the overall capacity of the LEC to utilize FAs as energy source.

**Fig. 5:**
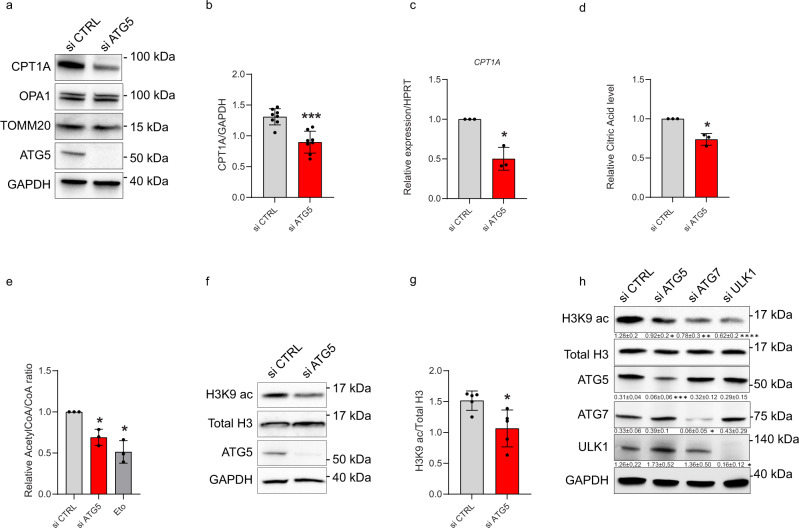
Autophagy regulates CPT1A expression and acetyl-CoA levels. Representative blots for indicated proteins (**a**) and densitometric quantification (**b**) of CPT1A levels in si CTRL or si ATG5 LEC. Mean ± SD, *N* = 3 biological replicates analyzed using unpaired Student’s t test (two-tailed), ***p = 0.0001. **c** RT-qPCR analysis of si CTRL and si ATG5 LEC. mRNA expression of CPT1A (relative to HPRT). Mean ± SD, *N* = 3 biological replicates analyzed using one sample t test (two-tailed), *p = 0.03. **d** Relative Citric acid levels in si CTRL and si ATG5 LEC measured via mass spectrometry. Mean ± SD, *N* = 3 biological replicates analyzed using one sample t test (two-tailed), *p = 0.03. **e** Relative acetyl CoA/CoA ratio measured via mass spectrometry in si CTRL, si ATG5, or etomoxir (Eto, 100 µM, 48 h) treated LEC. Mean ± SD, *N* = 3 biological replicates analyzed using one way ANOVA with Tukey’s test for multiple comparisons, *p < 0.05 vs si CTRL. Representative blots (**f**) and densitometric quantification (**g**) for H3K9 ac levels relative to total H3 of si CTRL and si ATG5 LEC. Mean ± SD, *N* = 5 biological replicates analyzed using unpaired Student’s t test (two-tailed), *p = 0.02. **h** Representative blots for indicated proteins with GAPDH as loading control of si CTRL, si ATG5, si ATG7, or si ULK1 LEC. Densitometric quantification is indicated beneath the blots (H3K9 ac levels relative to total H3 and ATG5, ULK1, and ATG7 levels relative to GAPDH). Mean ± SD, *N* ≥ 3 biological replicates analyzed using one way ANOVA with Tukey’s test for multiple comparisons, *p < 0.05, **p < 0.01, ***p < 0.001 and ****p < 0.0001 vs si CTRL.

Autophagy-depleted LEC displayed reduced CPT1A protein levels (Fig. 5a, b), whereas OPA1 and TOMM20 protein turnover remained unaltered (Fig. 5a and Supplementary Fig. 3a), suggesting that autophagy regulates the selective expression of this mitochondrial FA transporter. In line with the downregulation of PROX1-mediated gene expression in autophagy-compromised LEC, CPT1A transcript levels were reduced (Fig. 5c). A trend, albeit not significant at the RNA level, was observed for the inner mitochondria membrane-associated CPT2A (Supplementary Fig. 5a–c), which converts acylcarnitine back to acyl-CoA for oxidation in the matrix.

Along with the downregulation of PROX1, treatment of LEC with the NH_4_Cl/leupeptin cocktail also dampened the expression of CPT1A (Supplementary Fig. 5d, e). Instead, exposing LEC to a concentrated FA emulsion (lipid concentrate), stimulated both CPT1A and PROX1 protein expression in si CTRL LEC, but not in lipophagy-compromised LEC (Supplementary Fig. 5f).

We then tested if the reduced levels of CPT1A and impaired FAO and TCA in autophagy-compromised LEC, affected cytosolic acetyl-CoA, which is generated after the export of citrate from the mitochondria to the cytosol and its conversion by ATP citrate lyase (ACLY) in acetyl-CoA. MS analysis revealed that in ATG5-silenced LEC both citrate (Fig. 5d) and acetyl-CoA/CoA (Fig. 5e) levels were significantly reduced. Likewise, etomoxir reduced acetyl-CoA/CoA ratios to an extent similar to that observed in autophagy-compromised LEC (Fig. 5e). Thus hampering the ability of mitochondria to perform efficient FAO consistently dwindles acetyl-CoA/CoA ratio.

Because histone H3 acetylation at lysine 9 (H3K9ac) by p300 is a marker of active gene promoters sensitive to acetyl-CoA level^44^, which bolsters epigenetic regulation of PROX1 driven transcription of lymphatic genes^21^, we then tested if compromising autophagy in LEC reduced H3K9 acetylation. Loss of ATG5 (Fig. 5f, g), ATG7, or ULK1 (Fig. 5h) silencing led to a significant reduction in H3K9ac levels in LEC. Compromising ATG5 expression in LEC did not affect other histone modifications, such as H3K27 acetylation or H3K4me3 methylation (Supplementary Fig. 5g, h), which have been reported to be sensitive to inhibition of the mitochondrial complex III by antimycin A in cultured LEC^23^.

In summary, lipophagy sustains levels of acetyl-CoA for histone H3K9 acetylation and CPT1A levels.

### Acetate rescues the mitochondrial-PROX1 transcriptional circuit caused by loss of autophagy in LEC

We then investigated if supplementation of acetate, a precursor of acetyl-CoA which does not require a carnitine shuttle mechanism of mitochondrial transport^45^, could restore expression of lymphatic markers in autophagy-defective LEC.

**Fig. 6:**
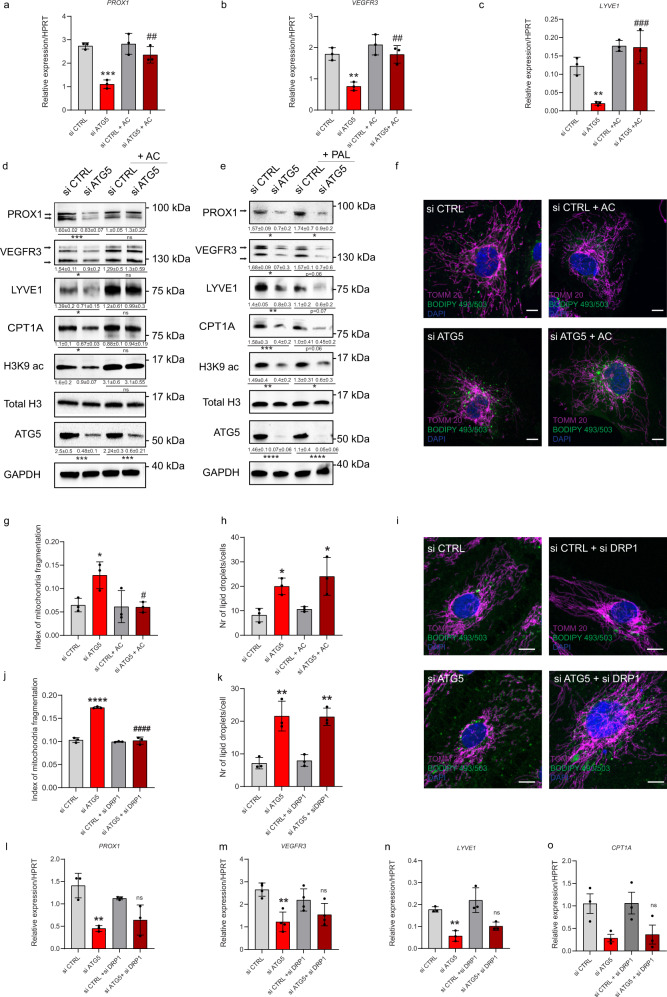
Acetate rescues the mitochondrial-Prox1 transcriptional circuit caused by loss of autophagy in LEC. **a**–**c** RT-qPCR analysis of si CTRL and si ATG5 LEC treated with sodium acetate (AC, 20 mM, 48 h) or vehicle. mRNA expression of Prox1, VEGFR3, and LYVE1 (relative to HPRT). Mean ± SD, *N* = 3 biological replicates analyzed by one-way ANOVA, with Tukey’s test for multiple comparisons, **p < 0.01 and ***p < 0.001 vs si CTRL, ##*p* < 0.01 and ###*p* < 0.001 vs si ATG5. **d** Representative blots for the indicated proteins in si CTRL or si ATG5 LEC treated with AC or vehicle. Densitometric quantification is indicated beneath the blots. Mean ± SD, *N* ≥ 3 biological replicates analyzed by one-way ANOVA, with Tukey’s test for multiple comparisons, *p < 0.05,***p < 0.001. **e** Representative blots for indicated proteins in si CTRL and si ATG5 LEC treated with palmitate (PAL, 500 nM, 48 h) or BSA. Densitometric quantification is indicated beneath the blots. Mean ± SD, *N* ≥ 3 biological replicates analyzed by one-way ANOVA, with Tukey’s test for multiple comparisons. *p < 0.05, **p < 0.01, ***p < 0.001 and ****p < 0.0001. **f** Representative immunofluorescent images of si CTRL and si ATG5 treated with AC or vehicle and stained for mitochondrial protein TOMM20 and BODIPY 493/503. Nuclei are stained with DAPI. Scale bars represent 10 µm. **g** Quantification of mitochondrial fragmentation index (number of mitochondria/ total mitochondrial area). Mean ± SD, *N* = 3 biological replicates analyzed by one-way ANOVA, with Tukey’s test for multiple comparisons, *p < 0.05 vs si CTRL, #*p* < 0.05 vs si ATG5. Mean represents mean per independent experiment, with a minimum of 12 cells analyzed per condition per independent experiment. **h** Quantification of lipid droplet number per cell. Mean ± SD, *N* = 4 biological replicates analyzed by one-way ANOVA, with Tukey’s test for multiple comparisons, *p < 0.05 vs si CTRL. Mean represents mean per independent experiment, with a minimum of 29 cells analyzed per condition per independent experiment. **i** Representative super resolution AiryScan images of si CTRL and si ATG5, si CTRL + si DRP1, and si ATG5 + si DRP1 LEC stained for the mitochondrial protein TOMM20 and BODIPY 493/503. Nuclei are stained with DAPI. Scale bars represent 10 µm. **j** Quantification of mitochondrial index of fragmentation (number of mitochondria/ total mitochondrial area). Mean ± SD, *N* = 3 biological replicates analyzed by one-way ANOVA, with Tukey’s test for multiple comparisons, ****p < 0.0001 vs si CTRL, ####*p* < 0.0001 vs si ATG5. Mean represents mean per independent experiment, with a minimum of 35 cells analyzed per condition per independent experiment. **k** Quantification of lipid droplet number per cell. Mean ± SD, *N* = 3 biological replicates analyzed by one-way ANOVA, with Tukey’s test for multiple comparisons, **p < 0.01 vs si CTRL. Mean represents mean per independent experiment, with a minimum of 47 cells analyzed per condition per independent experiment. **l**–**o** RT-qPCR analysis in si CTRL, si ATG5, si CTRL + si DRP1 and si ATG5 + si DRP1 LEC. mRNA expression of PROX1, VEGFR3, LYVE1, and CPT1A (relative to HPRT). Mean ± SD, *N* = 3 (**l**, **n**, **o**) and *N* = 4 (**m**) biological replicates analyzed by one-way ANOVA, with Tukey’s test for multiple comparisons, **p < 0.01 vs si CTRL and *p* = ns vs si ATG5.

Acetate supplementation to ATG5-silenced LEC indeed restored the expression of PROX1, VEGFR3, and LYVE1, both at the RNA (Fig. 6a–c) and protein level (Fig. 6d) to that of si CTRL LEC. Similar rescuing effects of acetate on protein and RNA level were observed upon silencing of ULK1 (Supplementary Figure Fig. 6a–d) or ATG7 (Supplementary Fig. 6e–h). Notably, both the defects in H3K9 acetylation and CPT1A levels observed in LEC deprived of autophagy were reversed by acetate supplementation (Fig. 6d). In contrast, providing palmitate that requires CPT-mediated shuttle to enter mitochondria, failed to rescue PROX-1 lymphatic gene expression in autophagy-incompetent LEC (Fig. 6e and Supplementary Fig. 6i–l).

Pharmacological inhibition of ACLY curbed both the overall expression of LEC markers in si CTRL and the rescue effect of acetate on ATG5-silenced LEC (Supplementary Fig. 6m). This suggests that acetyl-CoA preserves LEC markers and that the action of acetate likely relies on the epigenetic regulation of PROX-1 transcription as shown previously^46^.

Providing acetate to LEC-depleted cells, as exogenous fuel for FAO, re-established a tubular mitochondrial network characteristic of respiring mitochondria (Fig. 6f, g), but did not alter LDs turnover (Fig. 6f, h). To clarify whether restoring mitochondrial tubular morphology in ATG5-compromised LECs, was per se sufficient to regain LEC specific markers, we silenced the GTPase dynamin-related protein (DRP1), a main mitochondrial fission regulator shuttling between the cytosol and the mitochondria surface^47^. Consistent with their fragmented mitochondrial morphology, ATG5-silenced LEC displayed a significant increase in the level of the DRP1 activating S616 phosphorylation (Supplementary Fig. 6n) and enhanced recruitment of DRP1 to the mitochondrial network as compared to si CTRL LEC (Supplementary Fig. 6o). DRP1 silencing (Supplementary Fig. 6p), significantly rescued the mitochondria fragmentation phenotype of ATG5-depleted LEC (Fig. 6i, j) but failed to alter LD’s number (Fig. 6i, k) and to rescue *PROX1, VEGFR3*, *LYVE1* and *CPT1A* expression levels (Fig. 6l–o).

Thus, when degradation of LDs is inhibited, exogenous provision of acetate recovers the elongated morphology of FAO-proficient mitochondria and fuels the transcriptional lymphatic program, whereas the sole rescue of a tubular mitochondrial network in lipophagy-compromised conditions fails to do so.

### Acetate rescues LEC function in autophagy inhibited cells in vitro and injury-driven lymphangiogenesis in LEC-ATG5 KO mice

**Fig. 7:**
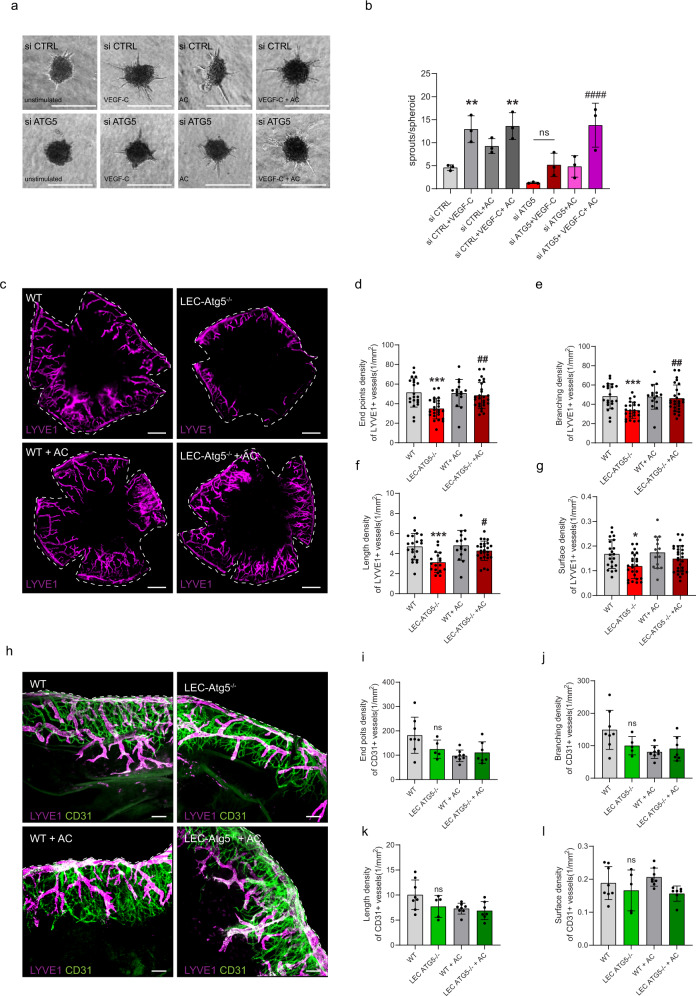
Acetate rescues LEC function in autophagy inhibited cells in vitro and injury-driven lymphangiogenesis in LEC-ATG5 KO mice. **a** Representative spheroid images from si CTRL and si ATG5 LEC under basal conditions, stimulated with recombinant VEGF-C (100 ng/mL) alone, supplemented with sodium acetate (AC, 20 mM, 48 h) alone or in combination. Scale bars represent 100 µm. **b** Quantification of sprouts per spheroid. Mean ± SD, *N* = 3 independent donors analyzed using Two-way ANOVA, with Tukey’s test for multiple comparisons **p < 0.05 vs si CTRL, ####*p* < 0.0001 vs si ATG5. Mean represents mean per independent experiment, with a minimum of 10 spheroids analyzed per condition per independent experiment. **c** Representative immunofluorescent images of whole corneal mounts (dashed lines) of wild type (WT) and LEC-Atg5 knock out mice (LEC-Atg5^−/−^) treated with vehicle or sodium acetate (AC, 400 µl 0.5 M i.p daily) stained for LYVE1, 8 days post corneal cauterization. Scale bar represents 1 mm. **d**–**g** Quantification of the number of end points, number of branch points, average of cumulative length, and surface density for LYVE1+ lymphatic vessels. Mean ± SD, *N* = 21 corneas for WT, *N* = 24 corneas for LEC-Atg5^−/−^, *N* = 15 corneas for WT + AC and *N* = 27 corneas for LEC-Atg5^−/−^ + AC analyzed using one-Way ANOVA corrected for multiple comparisons using Tukey’s test *p < 0.05, ***p < 0.001 vs WT, #*p* < 0.05 ## *p* < 0.01 vs LEC-Atg5−/−. **h** Representative immunofluorescent images of corneal sections dissected from WT and LEC-Atg5^−/−^ treated with vehicle or sodium acetate (AC), stained for LYVE1 and CD31, 8 days post corneal cauterization. Scale bar 100 µm. **i**–**l** Quantification for the number of end points, number of branch points, average of cumulative length, and surface density for the CD31+ blood vessels. Mean ± SD, *N* = 8 corneas for WT, *N* = 5 corneas for LEC-Atg5^−/−^, *N* = 8 corneas for WT + AC, and *N* = 7 corneas for LEC-Atg5^−/−^ + AC analyzed using one-Way ANOVA corrected for multiple comparisons using Tukey’s test. *p* = not significant.

To further portray the effects of acetate at the functional level, we then tested whether this FA precursor could restore VEGF-C induced sprouting responses in autophagy-deprived LEC. In si CTRL LEC, acetate had a marginal effect on spheroid sprouting in combination with VEGF-C (Fig. 7a, b). Notably, supplementing acetate restored sprout numbers of autophagy-defective LEC almost to control values (Fig. 7a, b), in line with the recovery of VEGFR3 protein levels (Fig. 6b, d).

Given the major rescuing effects of acetate observed in vitro, we then tested if acetate supplementation could restore corneal lymphangiogenesis in the LEC-ATG5^−/−^ mice. While acetate injection in WT mice did not exert significant effects, supplementing acetate to LEC-ATG5^−/−^ mice rescued injury-driven lymphangiogenesis (Fig. 7c). Especially LYVE1^+^ vessel branching, end points, and length (Fig. 7d–g) were restored to levels similar to those of WT treated animals, indicating that acetate restores responsiveness of autophagy-deprived LEC to pro-lymphangiogenic factors promoting migration and proliferation of LEC, under these inflammatory conditions. No significant effects were observed on blood vessel formation (Fig. 7h–l). Acetate did not significantly rescue the milder effects observed after CQ treatment on lymphatic branching and end points (Supplementary Fig. 7a–e). This suggests that CQ might inhibit additional (paracrine) inflammatory pathways regulating injury-driven lymphangiogenesis in vivo, which are insensitive to acetate.

**Fig. 8:**
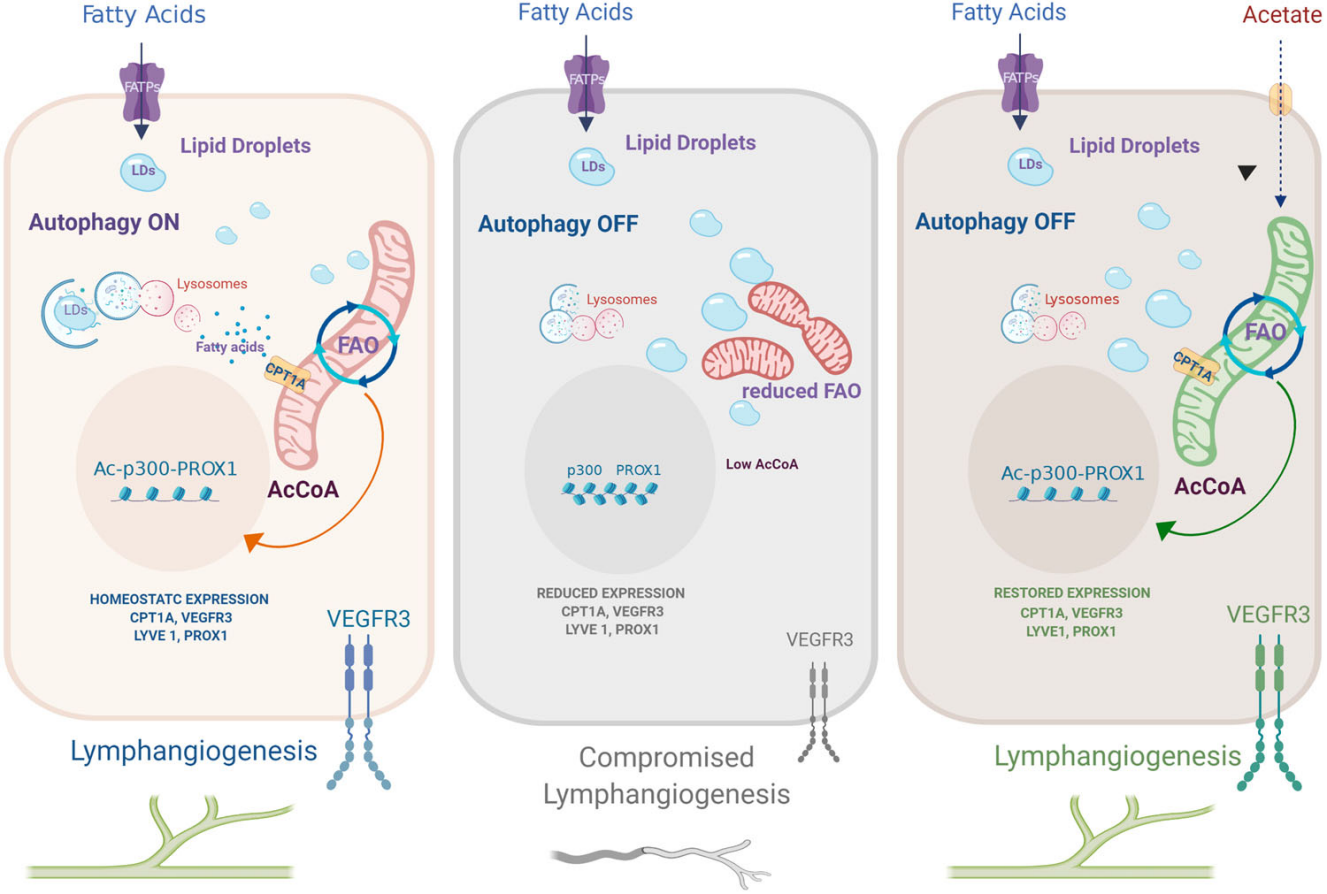
Lipophagy maintains lymphatic identity and lymphangiogenesis by supplying fatty acids to the mitochondria for fatty acid oxidation and PROX1-mediated gene expression. The figure depicts the main phenotypes of (left to right) autophagy-proficient LEC (autophagy ON), autophagy-deficient LEC (autophagy OFF), and their rescue with acetate, described in the study. Under homeostatic conditions (left panel), constitutive levels of autophagy favor the turnover of lipid droplets (LDs) in order to supply free fatty acids to the mitochondria and foster fatty acid oxidation (FAO). The actively respiring elongated mitochondria replete acetyl-CoA pools, which is used by the acetyltransferase p300 to acetylate histones at PROX1-target genes. This autophagy-regulated mechanism maintains PROX1-driven expression of lymphatic markers and VEGFR3-mediated lymphangiogenic signaling. When autophagy is compromised (center panel), LDs turnover is impaired causing accumulation of LDS in the cytosol. Under these conditions, the ability of mitochondria to perform FAO is compromised, the mitochondrial network is fragmented and acetyl-CoA levels drop to levels unable to support transcription of PROX1-driven lymphatic gene networks and lymphangiogenesis. This phenotype can be rescued by feeding LEC with the acetyl-CoA precursor acetate (right panel). Acetate rescues mitochondrial morphology and FAO and recovers PROX1-mediated expression of lymphatic genes, thus bypassing the lymphangiogenesis defects caused by genetic loss of autophagy. Image created with Biorender.com.

Thus, impaired corneal lymphangiogenesis caused by genetic loss of LEC-autophagy can be reversed in vivo by rescuing metabolic alterations through acetate supplementation (Fig. 8).

## Discussion

In this study, we demonstrate that LD degradation by lipophagy is a critical process sustaining metabolic homeostasis in LEC and postnatal lymphangiogenesis. We provide compelling evidence showing that impairing the autophagic machinery, results in the accumulation of TG- containing LDs and mitochondria which are unable to efficiently perform FAO and to maintain acetyl-CoA levels, required to support (epigenetic) regulation of PROX1-target genes.

While sequestration of FAs in LDs functions as a buffering mechanism to prevent the potential deleterious effect of free FAs and lipotoxicity^30,36,48^, selective lysosomal degradation of LDs during periods of nutrient unavailability, during cell growth or differentiation^37,49^ fosters metabolic processes and membrane biosynthesis^30^.

Our study unravels that compromising lipophagy results in defective trafficking of LDs to mitochondria to support FAO and altered mitochondria dynamics, which interrupt the mitochondrial-PROX1-driven transcriptional circuit, responsible for the expression of lymphangiogenic markers and underscores the intimate, but still elusive link between LD degradation, mitochondrial shape, and metabolism^40^.

While it is well established that the elongated mitochondrial shape is critical for proficient metabolic activity, current insights into the interaction between LDs and mitochondria dynamics are still limited^30,40^. Also, the molecular entities operating as lipophagy receptors are still largely undefined. In starved cells, preventing mitochondria fusion resulted in unmetabolised FAs, which were re-routed to LDs in order to avoid lipotoxicity^37^.

We show that in autophagy-depleted LEC, the supplementation of the FA precursor acetate recovers the elongated mitochondria morphology without altering the accumulation of LDs. On the other hand, rescuing mitochondria elongation by silencing DRP1 in autophagy-compromised LEC does not alter LD accumulation and fails to correct the expression of VEGFR3 and CPT1A. Together, these findings suggest that lipophagy in LEC operates primarily as a mechanism of FA-supply to the mitochondria and that LD turnover is not secondary to alterations of the mitochondria bioenergetic status. However, further research is required to completely clarify the complex interconnection between mitochondria dynamics and LD metabolism^36,38,49^ and to rule out that LD accumulation in autophagy deficient cells is a consequence of a reduction of CPT1A levels.

Given the specialized function of lymphatic vessels in lipid trafficking, it is plausible that LEC become particularly dependent on signaling mechanisms regulating FA storage and degradation to maintain metabolic fitness, homeostasis, and LEC specification. The finding that acetate supplementation restores LEC markers and rescues the corneal lymphangiogenesis deficits of the LEC-Atg5^−/−^ mice, unravels the key regulatory role that autophagy plays in the metabolic homeostasis of lymphatic vessels. Deletion of the QPC subunit of complex III in differentiating LEC of mouse embryos, results in loss of LEC fate, likely as a consequence of reduced citrate generated in the mitochondria and histone hypoacetylation at *VEGFR3* and *PROX1* promoters observed in cultured LEC^23^. While this study^23^ further unravels the crucial relevance of the mitochondria-gene expression circuit in a model of developmental lymphangiogenesis, it also suggests that specific mitochondrial-derived metabolites may fine-tune LEC fate, which is highly plastic^7,23,24^. Notably, chemical inhibition of mitochondrial complex III by antimycin A^23^ evoked a landscape of histone modifications in LEC, namely H3K27ac and H3K4m3 methylation, which we do not portray in our study. An increase in H3K27ac in the *NRP1* and *ICAM1* loci after antimycin A was suggested as epigenetic mechanism of derepression of these BEC genes in LEC promoting dedifferentiation of LEC into BEC in vitro^23^. Instead, here we show that genetic inhibition of different autophagy genes reduced H3K9 acetylation, which is sensitive to acetyl-CoA levels^50^. Hence, differences in the landscape of histone modifications following alterations of mitochondrial metabolic pathways or inhibition of respiratory complexes, could explain why we did not observe a significant upregulation of BEC genes in cultured LEC upon loss of autophagy. This is an interesting assumption that needs to be experimentally explored in future studies addressing the full spectrum of histone modifications associated to mitochondria-derived metabolites and their impact on LEC identity.

Recent reports show that ketone bodies provided by high-fat, low-carbohydrate ketogenic diet, which are known autophagy stimulators^51,52^, improve lymphangiogenesis after corneal injury and myocardial infarction, and lymphatic vessel function and growth in a mouse model of lymphedema^53^. Hepatic loss of *Atg7* or *Atg5* significantly impairs LD degradation, FAO, and ketone bodies production upon fasting^54^, further highlighting the link between tissue-specific catabolism of FAs and autophagy. Since PROX1 is also expressed in the hepatocytes of the adult liver^55^, it would be interesting to assess whether in our Prox1-CRE-ERT2 transgenic mouse model starvation further aggravates the lymphangiogenic defects observed after loss of *Atg5* in lymphatic EC and hepatocytes. Considering that LEC-autophagy provides the fuel supply to maintain FAO and ultimately lymphatic proliferation and migration, it is tempting to speculate that dietary or life-style conditions that stimulate autophagy (e.g., caloric restriction, keto-diet, improved exercise) may facilitate restoring lymphatic functions in pathological conditions such as obesity or lymphedema.

## Methods

### Cell culture and RNA interference

Human dermal LECs were commercially purchased from Promocell (C-12217 cultured on dishes precoated with 0.1% gelatin (Sigma Aldrich) and used between passages 2 and 8. LEC were grown in ECGMV2 added with SupplementMix (C-22211 and C-39226, Promocell). SiRNA transient transfection was performed twice on consecutive days using 40 nM, non-targeting siRNA (si CTRL), siRNA against human ATG5 (si ATG5), ULK1 (si ULK1), ATG7 (si ATG7), and DRP1 (si DRP1) purchased from Dharmacon (D-001810, L-004374, L-005049, L-020112, and L-012092 respectively). Treatments with chloroquine (CQ), sodium acetate (AC), etomoxir (Eto), VEGF-C, palmitate, ATP citrate lyase inhibitor (ACLYi), ammonium chloride (NH4Cl) and leupeptin (C6628, S2889-250G, E1905, SRP3184, 14464-31-4, SML0784, 12125-02-9 and 108975 respectively, Sigma Aldrich) for assessing signaling and functional assays were done for 48 h. All cells were maintained in an incubator at 37 °C with 5% CO2 and 95% air.

### LC3-GFP transfection

26000 cells/cm^2^ were seeded in a 12 well plate on day one. siRNA transient transfection was performed following the protocol described above. The next day, plasmid (pBABEpuro GFP-LC3, addgene plasmid #22405) transfection (800 ng/well) was performed with Lipofectamine 2000 (Invitrogen, 16688), following the manufacturer’s protocol. Cells were fixed with 4% paraformaldehyde (J19943K2, Thermo Fisher Scientific) 24 h after plasmid transfection. Cell nuclei were stained with DAPI (D1306, Thermo Scientific). Fluorescence images were acquired using an inverted microscope (IX83, Olympus). Analysis was performed using NIH ImageJ software.

### Immunoblot analysis

Cells were lysed in a modified Laemli buffer (125 mM Tris-HCl, pH 6.8 buffer containing 2% SDS and 20% glycerol) with the addition of protease and phosphatase inhibitors (A32953, A32957 respectively, Thermo Fisher Scientific). Proteins were separated using SDS-PAGE under reducing conditions, transferred to a nitrocellulose membrane and examined by immunoblotting. Primary antibodies used were rabbit anti-ATG5 (12994S, CST), rabbit anti-ATG7 (1:1000, 8558S, CST), rabbit anti-LC3 (1:1000, 3868S, CST), rabbit anti-GAPDH (1:5000, 2118S, CST), rabbit anti-p62 (1:1000, p0067, Millipore), rabbit anti-β-actin (1:5000, A5441, Sigma-Aldrich), goat anti-LYVE1 (1:1000, AF2089, R&D systems), rabbit anti-PROX1 (1:1000, 11067-2, Proteintech), rabbit anti-VEGFR3 (1:1000, ab154079, Abcam), mouse anti-NR2F2 (1:1000, ab41859, Abcam), rabbit anti-CPT1 (1:1000, D3B3, CST) antibody, rabbit anti-CPT2 (1:1000, ab18114, Abcam), rabbit anti-acetyl histone H3 (lysine 9) antibody (1:1000, 9671, CST), rabbit anti-pan-acetyl histone H3 antibody (1:1000, 39139, Active Motif)), mouse anti-CD36 antibody (1:1000; ab17044, Abcam), mouse anti-ULK1 antibody (1:1000; ab56344, Abcam), rabbit anti-TOMM20 (1:1000; BD612278, BD Biosciences), mouse anti-OPA1 (1:1000; 612607, BD Biosciences), mouse anti-ENOS (1:1000; 610297, BD biosciences), rabbit anti-phospho DRP1 (Ser616) (1: 1000; 3455S, CST), mouse anti-DRP1 (1:1000; 611113, BD Biosciences), rabbit anti-H3K4me antibody (1:1000; ab8580, Abcam), rabbit anti-H3K27 ac (1:1000; 8173T, CST) and rabbit anti-FABP4 antibody (1:1000; 2120S, CST). Appropriate secondary antibodies were from Cell Signaling Technology and Thermo Fisher Scientific (Erembodegem, Belgium). Membranes were scanned using the Bio-Rad Chemidoc Imager (Bio-Rad Laboratories N.V.3). Quantification of western blot data was done using ImageLab software.

### Quantitative-real-time PCR

RNeasy Plus mini kit (74136, Qiagen) was used for RNA extraction and reverse transcription kit QuantiTect (205313, Qiagen) for CDNA generation. Gene expression was determined with ORA qPCR Green L mix (QPD0105, HighQu) utilizing the ABI 7,500 machine (Applied Biosystems) and analyzed using the delta delta Ct method. Primer sequences are available in Supplementary Table 1 (Supplementary Information File).

### Spheroid sprouting assay

Spheroids of LEC were generated from 2000 cells in hanging drops in ECGM2 containing 20% of methylcellulose (M6385, Sigma Aldrich). As previously described in ref. ^56^, spheroids were embedded in a collagen gel (08-115, Merck, Germany) and cultured in the respective medium of LEC. Depending on experimental settings, cells were stimulated with 100 ng/mL VEGF-C, treated with 25 µM CQ, 20 mM ammonium chloride (NH4Cl), and 100 µM leupeptin or supplemented with 20 mM AC and cultured for 48 h to analyze sprouting. For the mixed spheroids, LEC were collected 48 h after transfection and labeled with cell membrane labeling lipophilic dyes CellVue™ Jade and CellVue™ NIR780 (88-0876-16 and 88-0875-16 respectively, Thermo Fisher Scientific). Spheroids containing equal amounts of si CTRL transfected cells (green) and si ATG5 transfected cells were generated, embedded, and analyzed after 48 h. Images were taken in an inverted microscope (IX83, Olympus) and an analysis for number of sprouts per spheroid was performed using NIH ImageJ software.

### Scratch wound assay

The scratch wound was made on confluent LEC monolayers. Cell plates were washed twice to remove floating cells and treated with 500 μg/ml Mitomycin C (M4287, Sigma Aldrich) to block proliferation. A scratch wound was applied on the confluent LEC monolayer using a 200 µL tip. After scratch wounding and immediate photography, cultures were further incubated for 24 h and photographed again. Images were taken in an inverted microscope (IX83, Olympus). Migration distance was measured with NIH ImageJ software and is expressed as percentage wound closure.

### Proliferation assay via Thymidine incorporation

25000 cells were seeded per well on a 24-well plate. The next day, cells were incubated with 1 μCi ml^−1^ [3H]-thymidine (NET355L005MC, Perkin Elmer) for 24 h, fixed with 100% ethanol at 4 °C for 15 min, and precipitated using 10% trichloroacetic acid (T6399, Sigma Aldrich) and lysed with 0.1 N NaOH. Amounts of [3H]-thymidine incorporated into DNA were measured by scintillation counting (Scintillation counter, Perkin Elmer).

### Cell death

LEC were trypsinized 48 h post transfection and incubated with 2 µg/mL propidium iodide (PI) (P4170, Sigma). Dead cells were defined as PI-positive cells using AttuneTM Cytometer (Thermo Fisher Scientific). Quantification was performed using FlowJo software.

### BODIPY (493/503) staining

Cells were seeded on coverslips at the desired confluence. After 24 h, plates were washed with PBS (D8537, Sigma Aldrich) and incubated in the dark for 30 min with 2 µM BODIPY 493/503 (D3922, Thermo Fisher Scientific) staining solution prepared in ECGM2. Coverslips were washed 3 times in PBS, fixed in 4% PFA for 10 min, stained with DAPI for another 5 min, washed in PBS, and mounted in a drop of Prolong^®^ Gold (P36934, Thermo Fisher Scientific). Images were acquired on the *Nikon C2 Eclipse* Ni-E inverted confocal microscope, the super resolution LSM 880 AiryScan, and the Olympus IX73. Analysis was performed using NIH ImageJ software. For corneal tissue staining, dissected corneas were washed in PBS followed immediately by staining with 2 µM BODIPY 493/503 staining solution prepared in PBS, for 30 min in the dark. Corneas were washed for 30 min at room temperature, fixed in 70% ethanol for 1 h, washed with PBS, stained with DAPI for 10 min, and flat-mounted on a microscope slide. Images were acquired on the super resolution LSM 880 AiryScan and the Zeiss LSM780 microscope.

### Immunocytochemistry of LEC

In case of co-staining with BODIPY 493/503, cells were washed twice times in PBS. In all cases, cells were fixed in 4% PFA for 15 min, blocked (1× PBS / 5% normal goat serum (#5425, CST)/0.1% Saponin (S-4521 Sigma Aldrich) for 1 h and stained overnight at 4 °C using mouse anti-TOMM20 (1:500; BD 612278, BD Biosciences), rabbit anti-TOMM22 (1:100; MBS7605092, MyBiosource), mouse anti-LAMP1 (1:100; ab25630, Abcam) and mouse anti-DRP1 (1:100; 61113, BD Biosciences) prepared in blocking solution. Cells were washed and incubated with corresponding secondary antibodies (1: 200; A21235, A21244, and A11001, Thermo Fisher Scientific) for 2 h in the dark at room temperature. Cells were rinsed with PBS, stained with DAPI for 5 min, and mounted using Prolong^®^ Gold Antifade Reagent. Images were acquired on the *Nikon C2 Eclipse* Ni-E inverted confocal microscope and the super resolution LSM 880 AiryScan. Mitochondrial morphology was analyzed using the ‘Mito chondrial morphology’ macro from ImageJ/Fiji Software and colocalization was analyzed using JACoP plugin Manders coefficients or Coloc 2 Pearson coefficient from ImageJ/Fiji Software.

### MitoTracker green, MitoSox, and TMRM analysis via flow cytometry

Prior to staining with TMRM or MitoSox respective positive controls were prepared either by treating with 1 µM Antimycin A (A8674, Sigma Aldrich) for 30 min or 0.5 µM FCCP (1528-10, Sanbio) for 20 min. Then cells were washed, trypsinized, and collected in endothelial growth medium. We stained in parallel for MitoSox 2.5 µM and TMRM 20 nM or stained for MitoTracker green 50 nM (M36008, T-668, M7514, Thermo Fisher Scientific respectively) for 1 h. Samples were then washed two times with cold PBS and read using AttuneTM Cytometer (Thermo Fisher Scientific). Analysis was performed using FlowJo software.

### Pulse chase experiments

Palmitate (PAL) was conjugated to BSA following the standard protocol from Seahorse Biosciences. BODIPY (D3821, Thermo Fisher Scientific) and PAL were conjugated in a 0.1% defatted bovine serum albumin solution for 30 min at 37 °C. Cells were incubated with 0.5 µM BODIPY-PAL overnight and immediately, after 6 h or 24 h stained with 0.1 µM Nile red (N1142, Thermo Fisher Scientific) or MitoTracker Deep Red (M22426, Thermo Fisher Scientific) for 30 min. Live cells images were acquired using the super resolution LSM 880 AiryScan. ‘Mitochondrial morphology’ macro from ImageJ/Fiji Software and colocalization was analyzed using JACoP plugin Manders coefficients from ImageJ/Fiji Software.

### Lipid concentrate assay

Cells were cultured in a medium containing 10% dialyzed and lipid-depleted serum (obtained through overnight incubation with silica), to which different concentrations of a chemically defined lipid concentrated emulsion (11905031, Thermo Fisher Scientific) were added for 48 h.

### Seahorse real-time extracellular acidification (ECAR) and oxygen consumption (OCR) test

30000 (human LEC) or 60000 (murine LEC; see below) cells were seeded on Seahorse Xp culture plates (103022-100, Agilent) in Seahorse XF Assay Medium (103335-100, Agilent), pH 7.4 containing 10 mM Glucose (8769, Sigma Aldrich), 1 mM Sodium Pyruvate (S8636, Thermo Fisher Scientific) and 2 mM Glutamine (G7513, Sigma Aldrich), following the manufacturer’s instructions. Prior to analysis, cells were maintained for 1 h in a CO_2_-free incubator at 37 °C. OCR was measured after serial injections of 3 µM Oligomycin (75351, Sigma Aldrich), 2 µM phenylhydrazone (FCCP, 1528-10, Sanbio), and 0.5 µM Antimycin A (A8674, Sigma Aldrich). ECAR was measured after serial injections of 80 mM glucose, 3 µM oligomycin, and 500 mM 2-deoxy-D-glucose (D-6134, Sigma Aldrich). Analysis was performed using Seahorse Wave Desktop Software (Agilent).

### Mitostress test for B-oxidation via seahorse

24 h prior to the assay, cells were put in substrate limited DMEM medium containing 0.5 mM Glucose, 1 mM Glutamine, 0.5 mM L-Carnitine (8400920025, Sigma Aldrich) and 1% fetal bovine albumin (FBS). 45 min prior to the assay, cells were washed and placed in KHB medium supplemented with 2.5 mM glucose, 0.5 mM L-carnitine, and 5 mM Hepes (12509079, Gibco), pH 7.4 in a CO_2_-free incubator at 37 °C. 40 µM Etomoxir (Eto) (E1905, Sigma Aldrich) was added in specific conditions 15 min before the start of the assay. Analysis was performed using Seahorse Wave Desktop Software (Agilent).

### Fatty acid oxidation assay

After 48 h of transfection, LEC were supplemented with 100 µM unlabeled palmitate, 50 µM carnitine (Sigma-Aldrich), and 2 mCi/mL [9,10-^3^ H]-palmitic acid (Perkin Elmer) for 18 h in complete endothelial growth medium. Then, supernatants were collected into glass vials sealed with rubber stoppers. As a readout for the assay, ^3^H_2_O was captured in Whatman paper soaked with H_2_O over a period of 48 h at 37 C^57^. Radioactivity was determined by liquid scintillation counting.

### Mouse models

Animal procedures were approved by the Ethical Committee for Animal Experimentation (ECD), Laboratory Animal Center of the KU Leuven, Leuven, Belgium and were performed in accordance with the institutional and national guidelines and regulations. For all experiments mice were kept at 24–25 °C, 40% humidity, group-housed in standard cages under a 12 h light/dark cycle, and fed ad libitum with standard animal chow and water. Mice from the EC-specific inducible Cre-driver line Prox1-cre^*ERT2*^^58^ were crossed with Atg5^fl/fl^ mice ^59^ to obtain mice with LEC-specific deletion of the *Atg5*gene. These lines were on a 100% C57BL/6 background. For experiments in this study, we used mice expressing Cre (Prox1-Cre+^*ERT2*^; *Atg5*^*fl/fl*^), referred to as LEC-Atg5^−/−^ and their Cre-negative littermates (Prox1-Cre-^*ERT2*^; *Atg5*^*fl/fl*^), referred to as WT. Both female and male mice were included in the experiments between the ages of 8-12 weeks. Tamoxifen (T5648, Sigma Aldrich) injection (i.p.50 mg/kg) was done daily for 5 consequent days 1 week prior to surgical procedure and Cre-expression was confirmed through PCR for genomic DNA using primers encompassing the floxed region, by the appearance of a 125 bp band.

### Corneal cauterization lymphangiogenesis assay

The cauterization assay was carried out as previously described^16,60^. Eight week old female and male mice, after anaesthetization with intraperitoneal injection of ketamine hydrochloride (100 mg/kg body weight, Thea), xylazine (10 mg/kg body weight, VMD), and the local anesthetic (Unicaïne 0.4%; Thea Pharma, Wetteren, Belgium), were cauterized in the cornea using an ophthalmic cautery (Optemp II V; Alcon Surgical, Fort Worth, TX). Intraperitoneal (i.p.) injections were done daily with 400 μl of a 0.5 M sodium acetate (AC), 50 mg/kg chloroquine (CQ) or 30 mg/kg etomoxir (Eto) solution while PBS was injected as a vehicle control, starting the day after corneal cauterization. Mice were euthanized by cervical dislocation nine days post-injury, eyes were removed and corneas dissected for histological analysis. Corneas were fixed in 70% ethanol for 1 h at room temperature, blocked in 3% BSA-3% Gloria milk (Nestlé) for another hour, washed with PBS, and incubated overnight with anti-LYVE1 (1:100, R&D systems, AF2125), anti-CD31 (1:100, Pharmingen, 553370), anti-ATG5 (1:100, 847410, Biolegend), anti-LC3B (1:100, 3868S, CST) or anti-VEGFR3 (1:100, 14-5988-82, Thermo Fisher Scientific) diluted 1/200 in PBS-1% BSA. After 1 h washing in PBS at room temperature corneas were subsequently incubated with respective secondary antibodies (A21222, A11081, and A21206, Thermo Fisher Scientific) diluted 1/200 in PBS-1% BSA for 2 h. Corneas were washed in PBS for 1 h, stained with DAPI for 5 min, and flat-mounted on a microscope slide with Vecta-shield mounting medium (Vector Laboratories) and imaged using a Leica DMI6000, super resolution LSM 880 AiryScan, and Leica sp8x confocal microscope. Lymphatic and blood vasculatures were quantified with the toolbox of MATLAB 8.3 (R2014a) software, and the following parameters were analyzed: area, branching (number of bifurcations), end-point (number of sprout tips), and length densities as described in ref. ^16^. Analysis of VEGFR3 fluorescence intensity was performed using NIH ImageJ software.

### Corneal pocket assay

Assay was performed as previously described^25^. Following the same sedation procedure as above, an incision of 0.5 mm was made with a straight keratome knife into the cornea. Pellets containing 300 ng VEGF-A (493-MV-025, R&D systems) were placed in the pocket 2 mm from the limbus. Mice were euthanized by cervical dislocation seven days post-injury, eyes were removed and corneas dissected for histological analysis. Staining was performed as mentioned above.

### Isolation of murine LEC

Murine endothelial cells were isolated from lungs after treatment with collagenase A and selected in a magnetic field after the cultures were incubated with magnetic beads (11533D CELLection, Invitrogen) coated with anti-mouse CD102 antibody (1:50, 553326 BD Biosciences) for the first selection. Endothelial cells were then released from beads and reselected with magnetic beads conjugated with anti-mouse Podoplanin anti-PDPN (8.1.1) (1: 50, BioLegend) in order to obtain CD102+ and Podoplanin+ cells. Cells were cultured in DMEM/F12 supplemented with 20% FCS, 100 u/ml penicillin, 100 µg/ml streptomycin, 2mM l-glutamine, 25 μg/ml Heparin, 75 μg/ml endothelial mitogen. Cultures from WT and LEC-ATG5 ^−/−^ mice were started simultaneously.

### In vivo CD36 analysis

Lung tissues were transferred into a gentleMACS C tube (130-093-237, Miltenyi Biotec) containing digestion medium (KnockOut™ DMEM (10829018, Thermo Fisher Scientific), penicillin/streptomycin (P4333, Sigma Aldrich), 2× Antibiotic-Antimycotic (A5955, Sigma Aldrich), 1 mM sodium pyruvate (S8636, Sigma Aldrich), 1× MEM Non-Essential Amino Acids Solution (M7145, Sigma Aldrich) supplemented with 0.1% collagenase II (17101015, Thermo Fisher Scientific), 0.25% collagenase IV (17104019, Thermo Fisher Scientific) and 15 μg/mL DNase (D4527-10KU, Sigma Aldrich) I. Each sample was further dissociated using the gentle MACS dissociator system (Miltenyi Biotec). Further purification of endothelial cells was done by magnetic enrichment of the CD31+ fraction (130-097-418, Miltenyi Biotec). Enriched cells were incubated with FC block (101319, Biolegend) for 5 min and then stained with anti-CD45 (1:200; 557659, BD Biosciences), anti-ter119 (1:200; 560509, BD Biosciences), Fixable Viability Dye eFluor™ 780 (1:1000; 65-0865-14, Thermo Fisher Scientific), anti-CD31 (1:200; 563356, BD Biosciences), anti-GP38 (1:100; 156207, Biolegend) and anti-CD36 (1:100; 102605, Biolegend) for 25 min. Cells were then washed and fixed with 1% PFA for 45 min. After fixation, cells were washed and resuspended in FACS buffer (554656, BD Biosciences) for analysis. Data was recorded in BD FACSymphony A5 instrument within 24 h of staining. Data were analyzed in FCS express V7 (De Novo Software).

### Lipidomic analysis

Cells were trypsinized, washed three times with cold DPBS and cell pellets were re-suspended in 0.8 ml DPBS. Lipid extraction and multiple reaction monitoring (MRM)-based phospholipid (semi)quantification analysis was performed as previously described in ref. ^61^. In brief, 0.7 ml of homogenized cells were mixed with 0.9 ml MeOH:HCl (1 M) (8:1), 0.8 ml CHCl_3_ and 200 μg per ml of the antioxidant 2,6-di-*tert*-butyl-4-methylphenol (Sigma). The organic fractions were evaporated under vacuum using a Savant Speedvac spd111v (Thermo Fisher Scientific) at room temperature and the remaining lipid pellet was stored at −20 °C under argon. Before mass spectrometry analysis, lipid pellets were reconstituted in running solution (CH_3_OH:CHCl_3_:NH_4_OH; 90:10:1.25; v/v/v). The lipid standards phosphatidylcholine (PC)25:0, PC43:6, sphingomyelin (SM)30:1, phosphatidylethanolamine (PE)25:0, PE43:6, phosphatidylinositol (PI)25:0, PI31:1, PI43:6, phosphatidylserine (PS)25:0, PS31:1 and PS37:4 (Avanti Polar Lipids) were added based on the amount of DNA of the original sample. Phospholipids were analyzed by electrospray ionization tandem mass spectrometry (ESI-MS/MS) on a hybrid quadrupole linear ion trap mass spectrometer (4000 QTRAP system, AB SCIEX) equipped with a TriVersa NanoMate robotic nanosource (Advion Biosciences) for automated sample injection and spraying as previously described^61^. Phospholipid profiling was executed by (positive or negative) precursor ion or neutral loss scanning at a collision energy of 50 eV or 45 eV, 35 eV, −35 eV, and −60 eV for precursor 184 (SM or PC), neutral loss 141 (PE), neutral loss 87 (PS) and precursor 241 (PI), respectively. Phospholipid quantification was performed by MRM, the transitions being based on the neutral losses or the typical product ions as described above. Typically, a 3 min period of signal averaging was used for each spectrum. The data were corrected for carbon isotope effects and chain length, and analyzed using in-house-developed software (RALP). As a background, the intensities of species detected in the ‘internal standards only’ spectra were considered after being divided by the ion suppression factor of each sample. The ion suppression factor was calculated for each sample separately by dividing the intensity of the standards in the ‘internal standards only’ spectrum by the intensity of the standards in the sample spectrum. Only the phospholipid species displaying an intensity of at least five times the blank value were taken into account. To quantify the total amount of phospholipids in a phospholipid class, the abundances of individually measured species within the phospholipid class were totaled. Data were normalized on the basis of DNA amount.)

### Electron microscopy

Transmission electron microscopy (TEM) was performed on a JEOL JEM1400 (JEOL Europe BV, Zaventem, Belgium) (VIB Bio Imaging Core, Leuven Platform). For TEM observations samples were fixed for 24 h with 2.5% glutaraldehyde at pH 7.3, buffered with 0.05 M sodium cacodylate. Prior to embedding in Agar 100 Resin (Agar Scientific, Stansted, UK) the material was post fixed in 2% OsO4 (buffered with 0.05 M sodium cacodylate, pH 7.3) and dehydrated in a graded acetone series. Semi-thin (± 1 mm) sections were cut with a Reichert Jung Ultracut E microtome and stained with 0.1% thionin—0.1% methylene blue. The ultra-thin (±70 nm) sections, on copper grids, were stained with uranyl acetate and lead citrate. Mitochondrial morphometric analysis was performed on electron microscopic images using Leica MetaMorph AF2.1 morphometric software.

### Super resolution and confocal microscopy

Super resolution images were recorded on a Zeiss LSM 880 – Airyscan, (Cell and Tissue Imaging Cluster (CIC), supported by Hercules AKUL/15/37_GOH1816N and FWO G.0929.15 to Pieter Vanden Berghe, University of Leuven, 63× magnification. Confocal images were acquired with Leica sp8x confocal microscope (KUL-VIB CCB), 60× magnification, Zeiss LSM780 microscope at 20× magnification (Neofluar 20×/0.75), Nikon C2 Eclipse Ni-E, Apo 60× (1,40 oil). We would like to thank the VIB BioImaging Core for training, support, and access to the instrument park Nikon C2 Eclipse Ni-E.

### Statistical analysis

All data are represented as mean ± SD. Statistical significance between two groups was determined by standard unpaired t-test with F-testing or one sample t-test. Unless otherwise indicated, statistical significance between multiple groups was determined by two-tailed one-way ANOVA to ensure comparable variance, then individual comparisons performed by Tukey’s post hoc test. In case of non-normality (using Anderson–Darling, D’Agostino & Pearson, and Shapiro–Wilk testing), Mann–Whitney or Kruskal Wallis tests were performed. Analysis was done in Prism v9.0f, GraphPad. GP p value style was used. */$/# represents a *p*-value < 0.05, **/##/$$ < 0.01, ***/###/$$$ *p* < 0.001 and ****/####/$$$$ *p* < 0.0001 where a *p*-value < 0.05 is considered significant.

### Reporting summary

Further information on research design is available in the Nature Research Reporting Summary linked to this article.

## Supplementary information

Supplementary Information

Reporting Summary

## Data Availability

All data presented in this study are included in this article. Source data, including raw lipidomics data, are provided with this paper. Source data are provided with this paper.

## Source data

Source Data
