# Supplementary material for: Lipid droplet degradation by autophagy connects mitochondria metabolism to Prox1-driven expression of lymphatic genes and lymphangiogenesis
Source: Nat Commun. 2022 May 19;13:2760. doi: 10.1038/s41467-022-30490-6 (PMC9120506; doi:10.1038/s41467-022-30490-6)
Supplement: Supplementary file 1 — Supplementary Information [file 41467_2022_30490_MOESM1_ESM.pdf]

Supplementary Information

LIPID DROPLET DEGRADATION BY AUTOPHAGY CONNECTS MITOCHONDRIA METABOLISM TO PROX1-DRIVEN EXPRESSION OF LYMPHATIC GENES AND LYMPHANGIOGENESIS

Odeta Meçe*, Diede Houbaert*, Maria-Livia Sassano, Tania Durré, Hannelore Maes, Marco Schaaf, Sanket More, Maarten Ganne, Melissa García-Caballero, Mila Borri, Jelle Verhoeven, Madhur Agrawal, Kathryn Jacobs, Gabriele Bergers, Silvia Blacher, Bart Ghesquière, Mieke Dewerchin, Johan V. Swinnen, Stefan Vinckier, María S. Soengas, Peter Carmeliet, Agnès Noel, Patrizia Agostinis

Supplementary Fig. 1

Supplementary Fig. 2

Supplementary Fig. 3

Supplementary Fig. 4

Supplementary Fig. 5

Supplementary Fig. 6

Supplementary Fig. 7

Supplementary Table 1: Primer sequences

Supplementary Table 2: Antibodies, Reagents and other Resourses

Gating strategies Extended data 3i

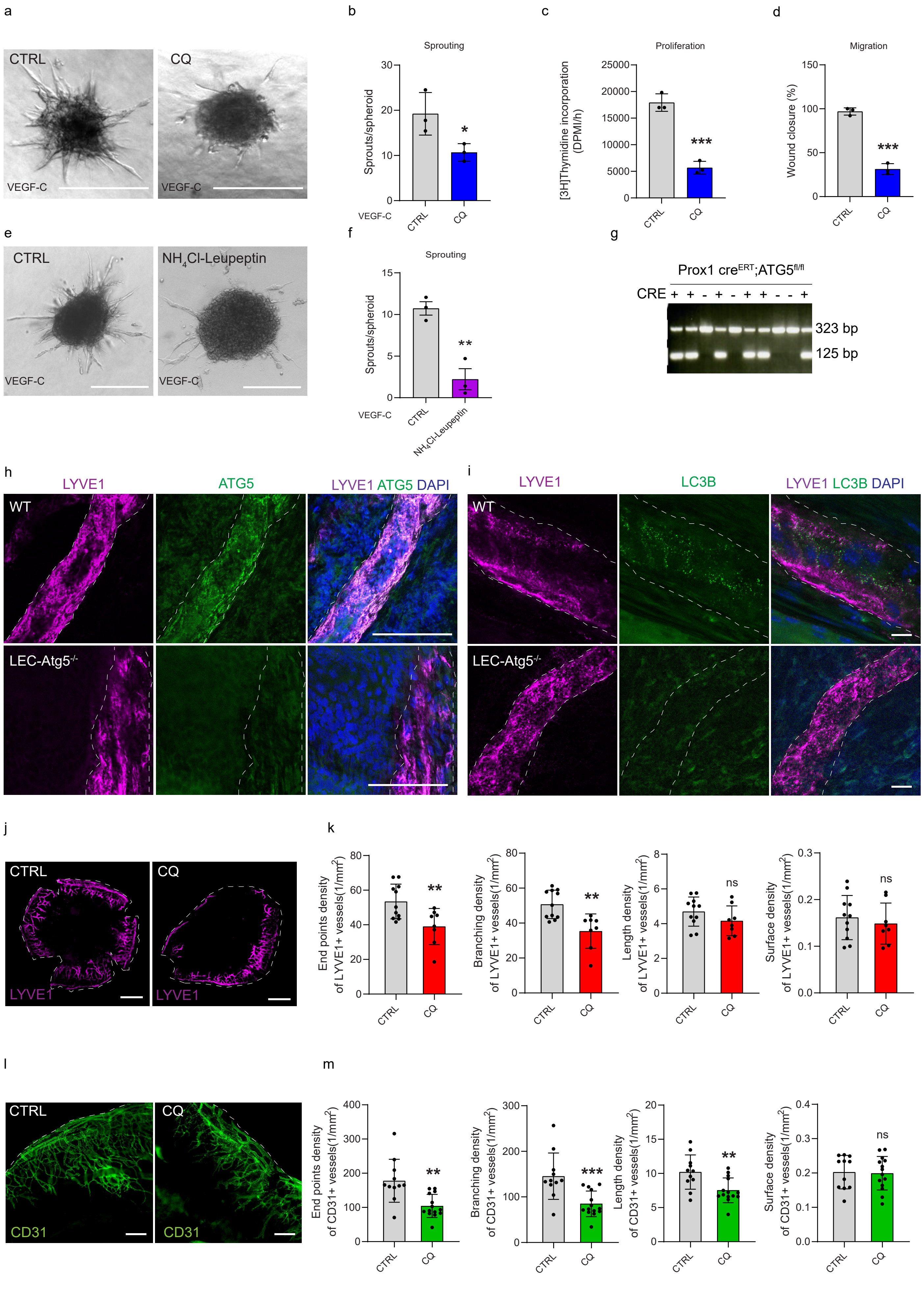

Supplementary Fig. 1

Supplementary Fig.1 | Effects of chloroquine and NH4Cl-Leupeptin on LEC function and confirmation of LEC-ATG5-/- mouse model.

a,b) Representative images (a) and quantification (b) of CTRL and CQ-(25 µM, 48h) treated LEC spheroids upon stimulation with VEGF-C (100 ng/mL, 48h). Scale bar represents 100 µm. Mean ± SD, N=3 independent donors analyzed using unpaired Student's t test (two-tailed), *p = 0.04. Mean represents mean per independent experiment, with a minimum of 10 spheroids analyzed per condition per independent experiment. **c)** [3H]Thymidine incorporation assay into DNA of CTRL and CQ-treated LEC upon stimulation with VEGF-C . Mean ± SD, N=3 biological replicates analyzed using unpaired Student's t test (two-tailed), ***p = 0.0005. **d)** Quantification of scratch wound healing for CTRL and CQ-treated LEC monolayers in the presence of 500 µ g/ml Mitomycin C. Mean ± SD, N=3 biological replicates analyzed using unpaired Student's t test (two-tailed), ***p = 0.0001. **e,f)** Representative images (e) and quantification (f) of CTRL and NH4CL-Leupeptin (20mM and 100µM, 48h) treated LEC spheroids upon stimulation with VEGF-C. Mean ± SD, N=3 independent donors analyzed using unpaired Student's t test (two-tailed), **p = 0.005. Mean represents mean per independent experiment, with a minimum of 9 spheroids analyzed per condition per independent experiment. **g)** Genotyping for the presence of Atg5 floxed (323bp) and excision band upon tamoxifen administration (125bp) in Prox1 creERT; ATG5fl/fl mice upon tamoxifen administration. Genotyping was performed prior to every in vivo experiment to confirm knockout of Atg5. **h)** Representative super resolution AiryScan images of LYVE1+ lymphatic vessels (dashed lines) from corneal sections dissected from wild type (WT) and LEC-Atg5 knock out mice (LEC-Atg5-/-) stained for ATG5 and ATG5 from corneas of WT and LEC-Atg5-/- mice. Note the absence of ATG5 immunoreactive signal in the LYVE1+ lymphatic vessel. Three corneas per condition were stained with similar results. Nuclei are stained with DAPI. Scale bar represents 100 µm. **i)** Representative super resolution AiryScan images of LYVE1+ lymphatic vessels (dashed lines) from corneal sections dissected from wild type (WT) and LEC-Atg5-/- mice stained for LC3B showing absence of autophagosomal bound LC3B in the lymphatic vessels of LEC-Atg5-/-mice. Three corneas per condition were stained with similar results. Nuclei are stained with DAPI. Scale bar represents 10 µm. **j)** Representative immunofluorescent images of whole corneal mounts (dashed lines) of vehicle treated or CQ-(50 mg/kg daily) treated wild type (WT) mice, stained for LYVE1, 8 days post corneal cauterization. Scale bar represents 1 mm. **k)** Quantification for the number of end points, number of branch points, average of cumulative length and surface density for LYVE1+ lymphatic vessels. Mean ± SD, N=11 corneas for CTRL and N=8 corneas for CQ analyzed using unpaired Student's t test (two-tailed), **p < 0.01. **l)** Representative immunofluorescent images of corneal sections dissected from vehicle treated or CQ-treated mice stained for CD31, 8 days post corneal cauterization. Scale bar represents 100 µm. **m)** Quantification for the number of end points, number of branch points, average of cumulative length and surface density for CD31+ blood vessels. Mean ± SD, N=11 corneas for CTRL and N=14 corneas for CQ analyzed using unpaired Student's t test (two-tailed), **p < 0.01 and ***p < 0.001.

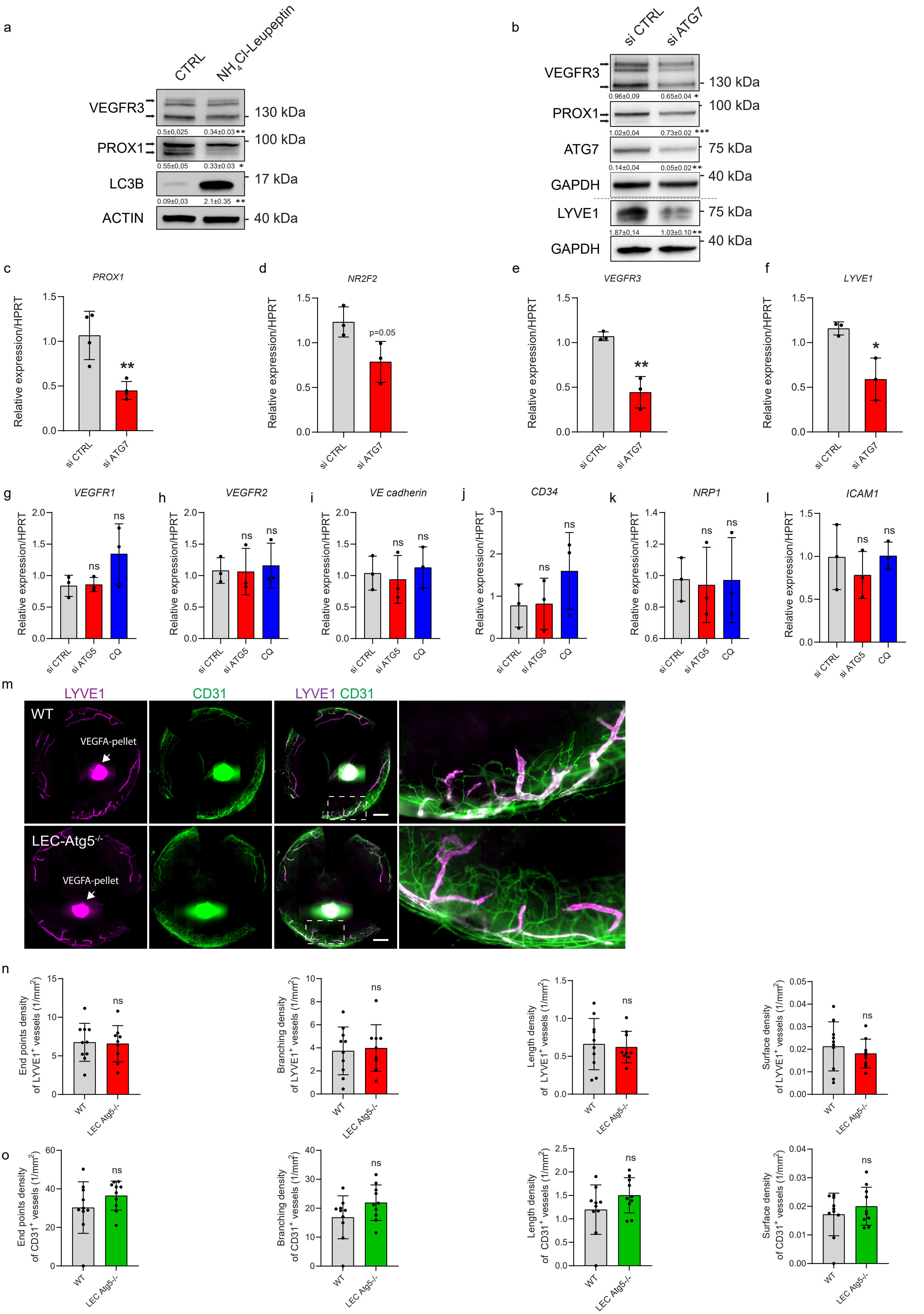

Supplementary Fig. 2

Supplementary Fig.2 | Autophagy deficiency does not impair general transcription of endothelial markers and responses to VEGF-A in vivo.

a) Representative blots for the indicated proteins in CTRL and NH4CL-Leupeptin-treated (20mM and 100µM, 48h) LEC. Densitometric quantification is indicated beneath the blots Mean ± SD, N=3 biological replicates analyzed using unpaired Student's t test, $*p < 0.05$ and $**p < 0.01$. **b)** Representative blots for the indicated proteins in si CTRL and si ATG7 LEC. Densitometric quantification is indicated beneath the blots. Mean ± SD, N≥3 biological replicates analyzed using unpaired Student's t test, $*p < 0.05$, $**p < 0.01$and $***p<0.001$. **c-f)** RT-qPCR analysis of si CTRL and si ATG7 LEC. mRNA expression of PROX1, VEGFR3 and LYVE1 (relative to HPRT). Mean ± SD, N=3 (d,e,f) and N=4 (c) biological replicates analyzed by unpaired Student's t test (two-tailed), $*p < 0.05$, $**p < 0.01$. **g-l)** RT-qPCR analysis of si CTRL and si ATG5 LEC or CQ-(25µM, 48h) treated LEC. mRNA expression of VEGFR1, VEGFR2, VE cadherin, CD34, NRP1 and ICAM1 (relative to HPRT). Mean ± SD, N=3 biological replicates analyzed by one-way ANOVA with Tukey's test for multiple comparisons. p=not significant. **m)** Representative immunofluorescent images from whole corneal mounts of wild type (WT) and LEC-Atg5 knock out mice (LEC-Atg5-/-) stained for LYVE1 and CD31, 5 days post pocket assay with VEGF-A (300ng/pellet) stimulation. Zoomed boxed areas show outgrowth of lymphatic vessel (magenta) and blood vessels (green). Scale bar represents 1 mm. **n)** Quantification for the number of end points, number of branch points, average of cumulative length and surface density for LYVE1+ vessels. Mean ± SD. N=10 corneas for WT and N=9 corneas for LEC-Atg5-/- analyzed using unpaired Student's t test (two-tailed), p=not significant. **o)** Quantification for the number of end points, number of branch points, average of cumulative length and surface density for CD31+ vessels. Mean ± SD. N=10 corneas for WT and N=9 corneas for LEC-Atg5-/- analyzed using unpaired Student's t test (two-tailed), p=not significant.

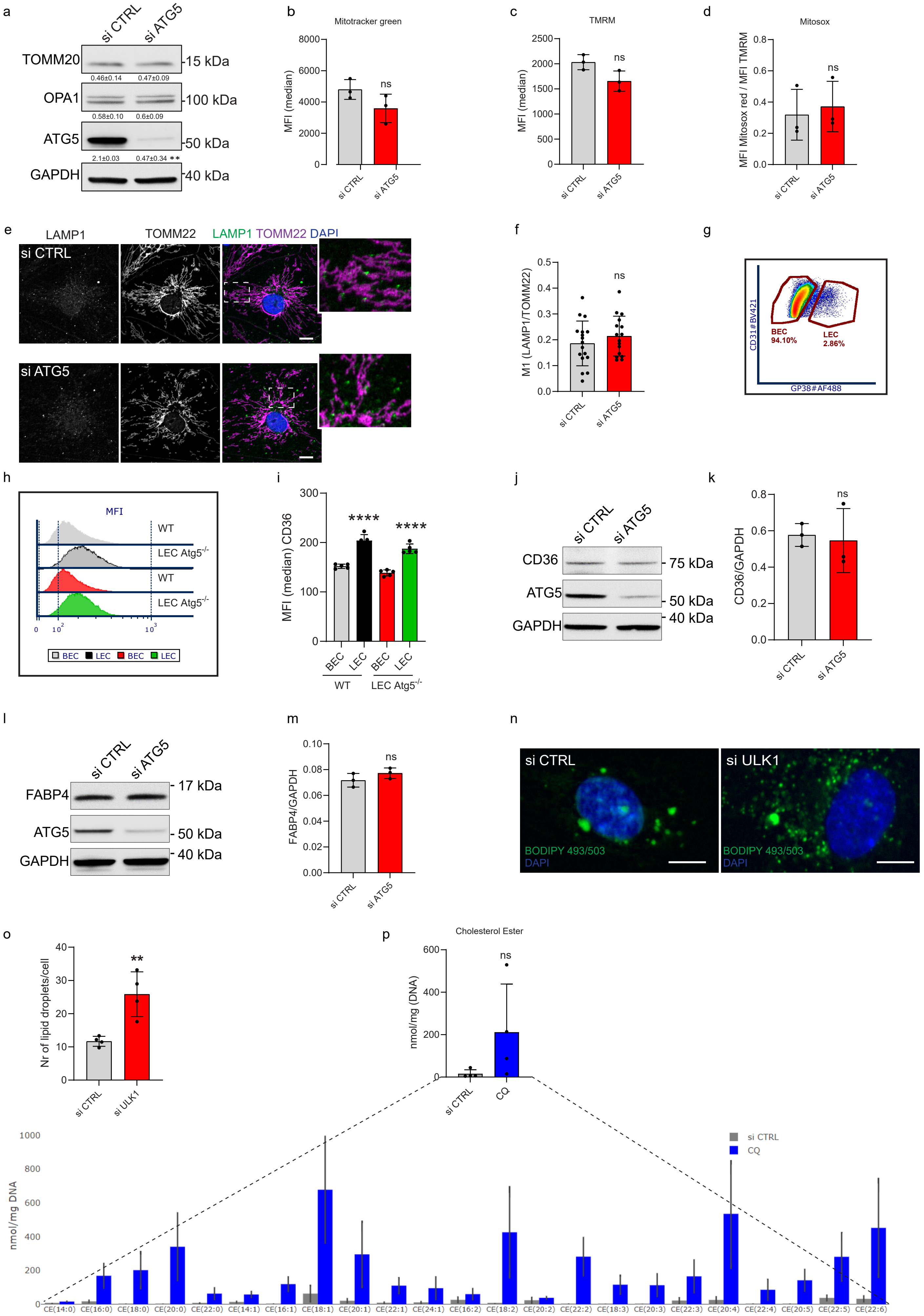

Supplementary Fig. 3

Supplementary Fig.3 | Effect of genetic or pharmacological inhibition of autophagy on mitochondrial protein turnover, mitochondrial ROS, mitochondrial membrane potential and lipid droplets.

a) Representative blots for the indicated proteins in si CTRL, si ATG5 LEC. Densitometric quantification is indicated beneath the blots. Mean ± SD, N=3 biological replicates analyzed using unpaired Student's t test, **p < 0.01. **b-d)** Flow cytometry analysis for mitochondrial mass using MitoTracker green (b), mitochondrial membrane potential using TMRM (c) and mitochondrial ROS using MitoSOX (d) in si CTRL and si ATG5 LEC. Mean ± SD, N=3 analyzed using unpaired Student's t test (two-tailed). p= not significant. **e)** Representative super resolution AiryScan images of si CTRL and si ATG5 immunostaining of TOMM22 and LAMP1. Nuclei are stained with DAPI. Scale bars represent 10 µm. **f)** Co-localization of LAMP1 and TOMM22 in si CTRL and si ATG5 LEC (Manders M1 coefficient). Mean ± SD, N=2 biological replicates with a minimum of 16 cells per condition in total analyzed using unpaired Student's t test (two-tailed), p=not significant. **g)** Flow cytometry histograms showing distinct populations of lymphatic (LEC, gated as double CD31+GP38+ double positive) and blood endothelial cells (BEC, gated as CD31+ positive), isolated from lung tissues of WT and LEC-Atg5-/- mice. **h,i)** median fluorescence intensity (MFI) of surface expression of CD36 in BEC and LEC from WT and LEC-Atg5-/- mice. Mean ± SD, N=5 mice analyzed by one-way ANOVA, with Tukey's test for multiple comparisons, ****p < 0.0001 to corresponding BEC. **j-m)** Representative blots for the indicated proteins (j,l) and densitometric quantification (k,m) in si CTRL, si ATG5 LEC. Mean ± SD, N=3 biological replicates analyzed using unpaired Student's t test (two-tailed), p=not significant. **n)** Representative images of BODIPY 493/503 staining of lipid droplets in si CTRL and si ULK1 LEC. Scale bars represent 10 µm. **o)** Quantification of lipid droplet number per cell. Mean ± SD, N=4 biological replicates analyzed using unpaired Student's t test (two-tailed), **p < 0.006. Mean represents mean per independent experiment, with a minimum of 29 cells analyzed per condition per independent experiment. **p)** Cholesterol ester (CE) bar plots calculated as an average of the lipid subspecies analyzed across si CTRL and CQ-treated LEC (25µM, 48h). Mean ± SD, N=4 analyzed by unpaired Student's t test (two-tailed), p = 0.14.

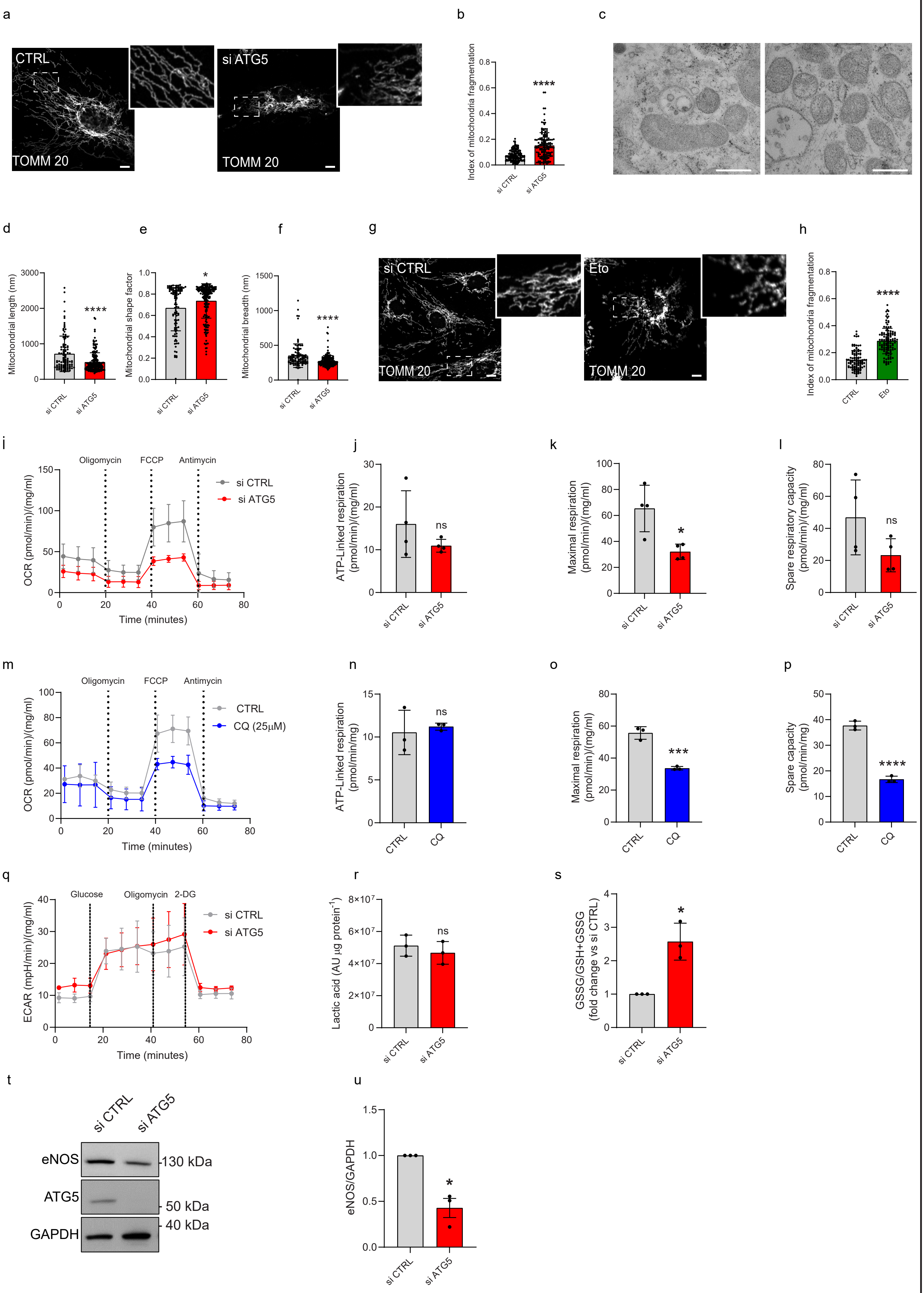

Supplementary Fig. 4

Supplementary Fig.4 | Assessment of mitochondrial respiration parameters in LEC upon ATG5 inhibition or CQ treatment and etomoxir effects on mitochondrial morphology.

a,b) Representative confocal immunofluorescent images (a) of the mitochondrial protein TOMM20 from si CTRL and si ATG5 LEC and quantification (b) of the mitochondrial fragmentation index (number of mitochondria/ total mitochondrial area). Zoomed boxed areas show the mitochondria shape. Mean ± SD, N=5 biological replicates with 144 cells for si CTRL and 153 cells for si ATG5 cells in total analyzed using Mann-Whitney test (two-tailed), ****$p < 0.0001$. Scale bars represent 10 µm. **c-f)** Representative electron microscopy images (c) of si CTRL and si ATG5 LEC (48h) and quantification of mitochondria morphometric parameters; mitochondrial length (d), shape (e) and breadth (f). Mean ± SD, N=104 (si CTRL) and N=189 (si ATG5) mitochondria from two biological replicates analyzed using Mann-Whitney test (two-tailed), *$p < 0.05$ and ****$p < 0.0001$. Scale bars represent 500 nm. **g,h)** Representative immunofluorescent images (g) of the mitochondrial protein TOMM20 in si CTRL LEC and after treatment with etomoxir (Eto, 100µM, 48h,) and quantification (h) of mitochondrial fragmentation index (number of mitochondria/ total mitochondrial area). Mean ± SD, N=3 biological replicates with a minimum of 104 cells per condition in total analyzed using Mann-Whitney test (two-tailed), ****$p < 0.0001$. **i)** Oxygen consumption rate (OCR) of si CTRL or si ATG5 LEC determined at baseline, after oligomycin, FCCP and antimycin treatment, analyzed via the Seahorse XFp Extracellular flux analyzer. **j)** ATP-linked respiration, **k)** Maximal respiration and **l)** Spare respiratory capacity calculated from OCR in i. Mean ± SD, N=4 biological replicates analyzed using unpaired Student's t test (two-tailed), *$p = 0.01$. **m)** OCR of CTRL or CQ-treated LEC (25µM, 48h) determined at baseline, after oligomycin, FCCP and antimycin treatment, analyzed via the Seahorse XFp Extracellular flux analyzer. **n)** ATP-linked respiration, **o)** Maximal respiration and **p)** Spare respiratory capacity calculated from OCR in m. Mean ± SD, N=3 biological replicates analyzed using unpaired Student's t test (two-tailed), ***$p < 0.001$ and ****$p<0.0001$. **q)** Extracellular acidification rate (ECAR) of si CTRL or si ATG5 LEC determined at baseline, after glucose, oligomycin and 2-deoxy-D-glucose (2-DG) treatment, analyzed via the Seahorse XFp Extracellular flux analyzer. **r)** Intracellular lactic acid levels. Mean ± SEM, N=3 biological replicates analyzed using unpaired Student's t test (two-tailed), $p = 0.47$. **s)** Oxidized glutathione calculated by the ratio of oxidized to total glutathione levels. Mean ± SEM, N=3 biological replicates analyzed using one sample t test (two-tailed), *$p = 0.04$. **t,u)** Representative blots (t) and densitometric quantification (u) of the indicated proteins in si CTRL and si ATG5 LEC. Mean ± SD, N=3 biological replicates analyzed using one sample t test (two-tailed), *$p = 0.03$.

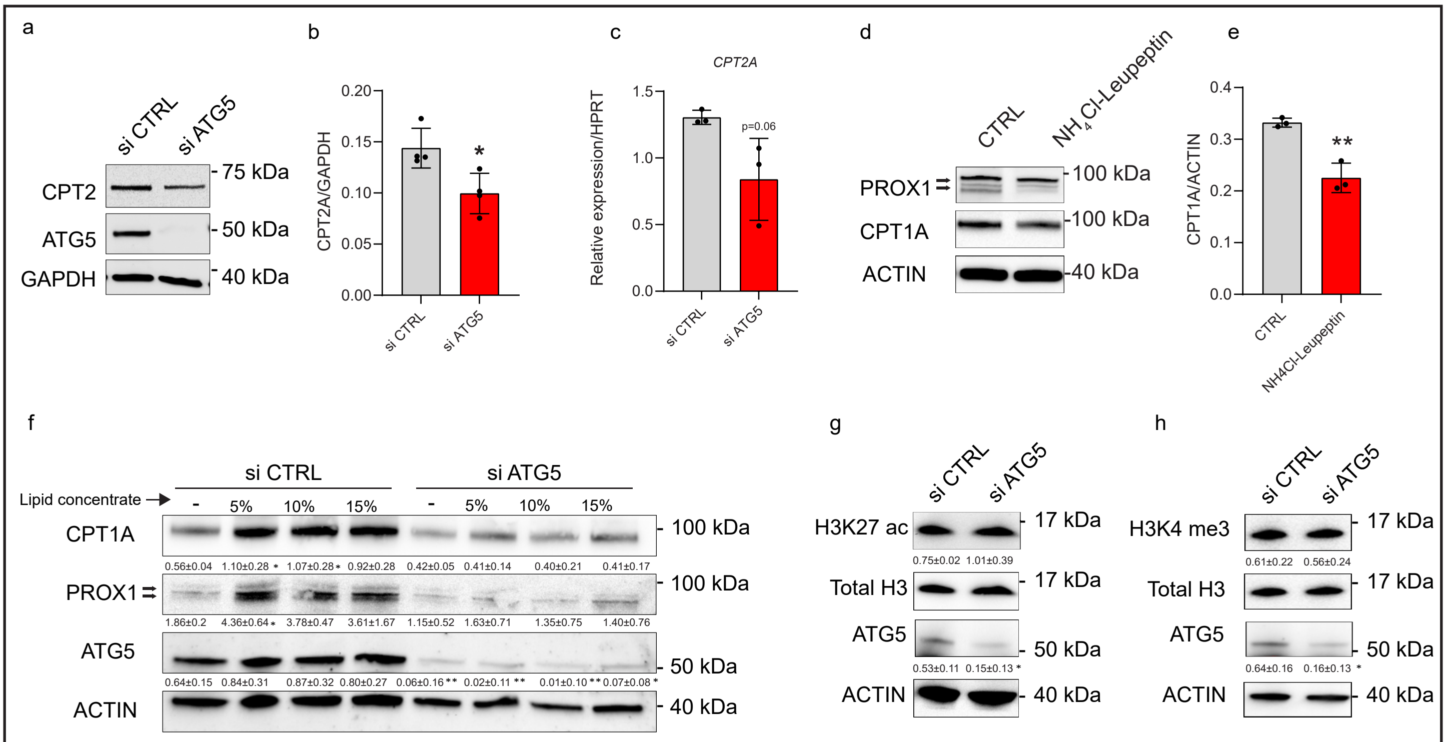

Supplementary Fig.5 | Expression of CPT1A and PROX1 is regulated by fatty acids and autophagy.

a,b) Representative blots (a) and densitometric quantification (b) for CPT2 levels of si CTRL or si ATG5 LEC . Mean ± SD, N=3 biological replicates analyzed using unpaired Student's t test (two-tailed), *p = 0.02. **c)** RT-qPCR analysis of si CTRL and si ATG5 LEC. mRNA expression of CPT2 (relative to HPRT). Mean ± SD, N=3 biological replicates analyzed by unpaired Student's t test (two-tailed). **d,e)** Representative blots (d) for indicated proteins and densitometric analysis (e) for CPT1A in CTRL and NH4Cl-Leupeptin (20mM and 100µM, 48h) treated LEC. Mean ± SD, N=3 biological replicates analyzed using unpaired Student's t test (two-tailed), **p < 0.004. **f)** Representative blots for indicated proteins in si CTRL and si ATG5 LEC upon stimulation with 5%, 10%, 15% lipid concentrate added to 10% dialyzed and lipid depleted serum for 48h. Densitometric quantification is indicated beneath the blots. Mean ± SD, N≥3 biological replicates analyzed using one way ANOVA with Tukey's test for multiple comparisons, *p < 0.05, **p<0.01, ***p<0.001 vs si CTRL. **g,h)** Representative blots for indicated proteins in si CTRL or siATG5 LEC. Densitometric quantification is indicated beneath the blots (H3K27 ac and H3K4 me3 levels relative to total H3 and ATG5 levels relative to ACTIN). Mean ± SD, N=3 biological replicates analyzed using unpaired Student's t test (two-tailed), *p < 0.05.

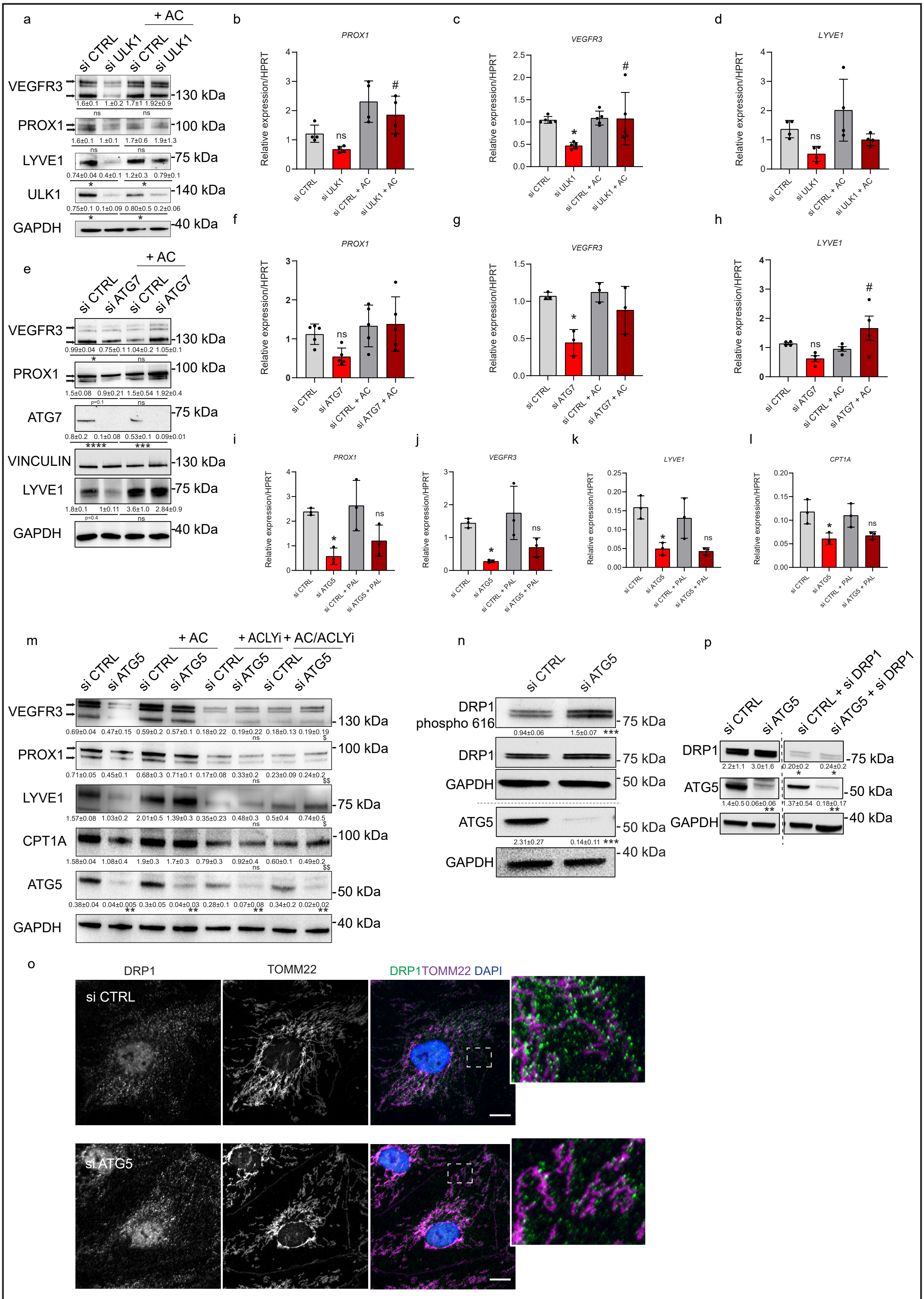

Supplementary Fig. 6

Supplementary Fig.6 | Rescue of PROX-1 driven lymphatic markers by acetate in autophagy compromised LEC.

a,e) Representative blots for indicated proteins in si CTRL, si ULK1 or si ATG7 LEC treated with sodium acetate (AC, 20mM, 48h) or vehicle .Densitometric quantification is indicated beneath the blots. Mean ± SD, N=3 biological replicates analyzed by one-way ANOVA, with Tukey's test for multiple comparisons, *p < 0.05, ***p<0.001 and ****p<0.0001. **b-d)** and **f-h)** RT-qPCR analysis in si CTRL, si ULK1 or si ATG7 LEC treated with AC or vehicle. mRNA expression of PROX1, VEGFR3 and LYVE1 genes (relative to HPRT).Mean ± SD N=3 (g), N=4 (b,d,h), N=5 (c,f) biological replicates analyzed by one-way ANOVA, with Tukey's test for multiple comparisons, *p < 0.05 vs si CTRL and #p < 0.05 vs si ULK1 or si ATG7. **i-l)** RT-qPCR analysis of si CTRL and si ATG5 LEC supplemented with palmitate (PAL, 500nM, 48h) or BSA. mRNA expression of PROX1, VEGFR3 and LYVE1 and CPT1A genes (relative to HPRT). Mean ± SD, N=3 biological replicates analyzed by one-way ANOVA, with Tukey's test for multiple comparisons, *p < 0.05 vs si CTRL and p = ns vs siATG5. **m)** Representative blots for indicated proteins in si CTRL and si ATG5 LEC upon ACLY inhibitor, AC alone or in combination (10 µM and 20 mM respectively, 48h). Densitometric quantifications indicated beneath the blots. Mean ± SD, N≥3 biological replicates analyzed by two-way ANOVA, with Tukey's test for multiple comparisons, **p < 0.01 vs si CTRL, $p<0.05, $$p<0.01 vs si ATG5 + AC. and ns vs si ATG5. **n)** Representative blots for indicated proteins in si CTRL and si ATG5 LEC. Densitometric quantification indicated beneath the blot. Mean ± SD, N=3 biological replicates analyzed by Student's t test, **p < 0.01. **o)** Representative super resolution AiryScan images of si CTRL and si ATG5 LEC stained with TOMM22 and DRP1. Nuclei are stained with DAPI. Scale bars represent 10 µm. **p)** Representative blots for indicated proteins in si CTRL, si ATG5, si CTRL + si DRP1 and si ATG5 + si DRP1 LEC. Densitometric quantification indicated beneath the blot. Mean ± SD, N≥3 biological replicates analyzed by one-way ANOVA, with Tukey's test for multiple comparisons, *p < 0.05 and **p < 0.01 vs si CTRL.

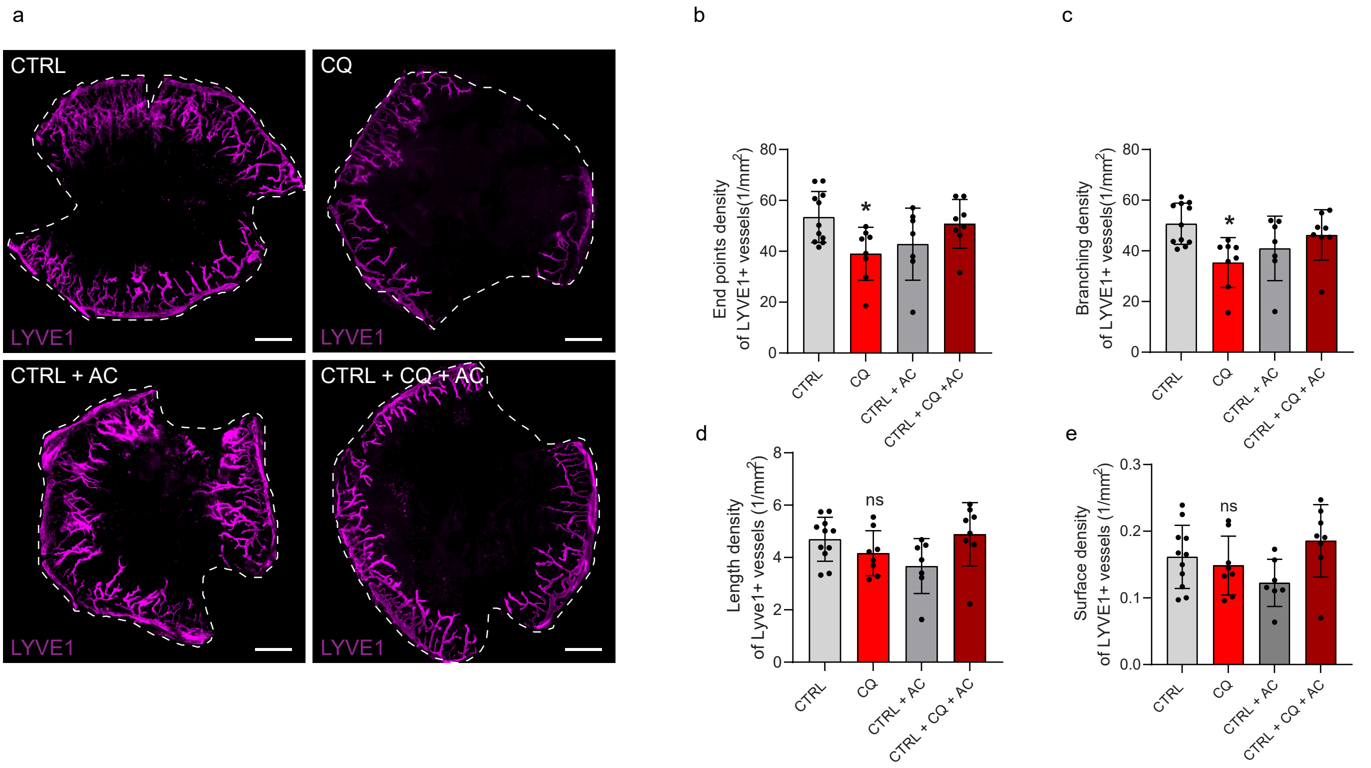

Supplementary Fig.7 | Acetate partly rescues injury-driven lymphangiogenesis in chloroquine treated mice.

a) Representative immunofluorescent images of whole corneal mounts (dashed lines) of vehicle control mice (CTRL) and mice daily treated with chloroquine (CQ, 50 mg/kg i.p daily), sodium acetate (AC, 400 µl 0.5 M i.p daily) or in combination stained for LYVE1, 8 days post corneal cauterization. Scale bar represents 1 mm. **b-e)** Quantification of the number of end points, number of branch points, average of cumulative length and surface density for LYVE1+ lymphatic vessels. Mean ± SD, N=11 corneas for CTRL, N=8 corneas for CQ, N=7 corneas for CTRL + AC and N=8 corneas for CQ + AC analyzed using one-Way ANOVA corrected for multiple comparisons using Tukey's test, *p < 0.05 vs CTRL.

Supplementary Table 1: Primer sequences

Gene	Forward primer	Reverse primer
Human *VEGFR3*	AAGATGTTTGCCCAGCGTAG	GCACTGTGGCATGAGGTCT
Human *LYVE1*	TGAAGGGGTAGGTGTGATGG	ATGACACCTGGATGGAAAGC
Human *PROX1*	TCACCTTATTCGGGAAGTGC	GAGCTGGGATAACGGGTATAAA
Human *NR2F2*	CCATAGTCCTGTTCACCTCAGA	AATCTCGTCGGCTGGTTG
Human *CPT1A*	CAATCGGACTCTGGAACCG	CCGCTGACCACGTTCTTC
Human *CPT2A*	CCACCATGCACTACCAGGA	TGGTGTCTTCAAGTTTGGGAAT
Human *HPRT*	GACCAGTCAACAGGGGACAT	GTGTCAATTATATCTTCCACAATCAAG
Human *ATG5*	CAACTTGTTTCACGCTATATCAGG	CACTTTGTCAGTTACCAACGTCA
Human *VEGFR1*	GCATACCTCACTGTTCAAGGA	GCCACACAGGTGCATGTT
Human *VEGFR2*	GCTCAAGACAGGAAGACC	GGTGCCACACGCTCT
Human *VE cadherin*	GCAGTCCAACGGACCAGAA	CATGAGCCTCTGCATCTTCC
Human *CD34*	GCGCTTTGCTTGCTGAGT	GGGTAGCAGTACCGTTGTTGT
Human *NRP1*	CACATTTCACAAGAAGATTGTGC	CATCAATTTTAATTTCTGGGTTCTTT
Human *ICAM1*	CCTTCCTCACCGTGTACTGG	AGCGTAGGGTAAGGTTCTTGC

Supplementary Table 2: Antibodies, Reagents and other Resourses

REAGENT or RESOURCE	SOURCE	IDENTIFIER
Antibodies		
Anti Goat IgG, HRP-linked	Thermo Fisher Scientific	PA1-28823<br>RRID:AB_10986856
anti-CD31	BD Biosciences	563356
anti-CD36	Biolegend	102605<br>RRID:AB_389348
anti-CD45	BD Biosciences	557659
Anti-Goat IgG, Alexa Fluor 488	Thermo Fisher Scientific	A21222<br>RRID: AB_10373853
anti-GP38	Biolegend	156207<br>RRID:AB_2814079
Anti-Mouse IgG, Alexa Fluor 488	Thermo Fisher Scientific	A11001<br>RRID:AB_2534069
Anti-Mouse IgG, Alexa Fluor 647	Thermo Fisher Scientific	A21235<br>RRID:AB_2535804
Anti-Mouse IgG, HRP-linked	Cell Signaling Technology	7076V<br>RRID: AB_330924
Anti-Rabbit IgG, Alexa Fluor 488	Thermo Fisher Scientific	A21206<br>RRID:AB_2535792
Anti-Rabbit IgG, Alexa Fluor 647	Thermo Fisher Scientific	A21222<br>RRID:AB_2535812244
Anti-Rabbit IgG, HRP-linked	Cell Signaling Technology	7074V<br>RRID:AB_2099233
Anti-Rat IgG, Alexa Fluor 546	Thermo Fisher Scientific	A11081<br>RRID:AB_141738
anti-ter119	BD Biosciences	560509
Goat anti-LYVE1	R&D systems	AF2089<br>RRID: AB_355144
Goat anti-LYVE1	R&D systems	AF2125<br>RRID: AB_2297188
Goat anti-Mouse IgG, Alexa Fluor 647	Thermo Fisher Scientific	A21235<br>RRID: AB_2535804
Hamster anti-PDPN	Biolegend	127401<br>RRID: AB_1089186
Mouse anti-ATG5	Biolegend	847410<br>RRID:AB_2814563
Mouse anti-CD36	Abcam	ab17044<br>RRID: AB_443600
Mouse anti-DRP1	BD Biosciences	611113<br>RRID:AB_398424
Mouse anti-DRP1	BD Biosciences	61113
Mouse anti-LAMP1	Abcam	ab25630

Mouse anti-NR2F2	Abcam	ab41859
Mouse anti-OPA1	BD Biosciences	612607<br>RRID: AB_399889
Mouse anti-TOMM20	BD Biosciences	BD 612278<br>RRID:AB_399595
Mouse anti-ULK1	Abcam	ab56344
Rabbit anti- Phospho-DRP1 (Ser616)	Cell Signaling Technology	3455S<br>RRID:AB_2085352
Rabbit anti-acetyl histone H3 (lysine 9)	Cell Signaling Technology	9671<br>RRID: AB_331532
Rabbit anti-ATG5	Cell Signaling Technology	12994S<br>RRID: AB_2630393
Rabbit anti-ATG7	Cell Signaling Technology	8558S<br>RRID:AB_10831194
Rabbit anti-CPT1	Cell Signaling Technology	D3B3<br>RRID: AB_2797857
Rabbit anti-CPT2	Abcam	ab181114
Rabbit anti-ENOS	BD Biosciences	610297
Rabbit anti-FABP4	Cell Signaling Technology	2120S<br>RRID: AB_2102466
Rabbit anti-GAPDH	Cell Signaling Technology	2118S<br>RRID: AB_561053
Rabbit anti-H3K27 ac	Cell Signaling Technology	8173T<br>RRID:AB_10949503
Rabbit anti-H3K4me3	Abcam	ab8580
Rabbit anti-LC3	Cell Signaling Technology	3868S<br>RRID: AB_2137707
Rabbit anti-p62	Millipore	p0067<br>RRID: AB_1841064
Rabbit anti-pan-acetyl-histone H3	Active Motif	39139<br>RRID: AB_2687871
Rabbit anti-PROX1	Proteintech	11067-2<br>RRID: AB_2268804
Rabbit anti-TOMM22	My Biosource	MBS7605092
Rabbit anti-VEGFR3	Abcam	ab154079
Rabbit anti-Vinculin	Cell Signaling Technology	#4650<br>RRID:AB_10559207
Rabbit anti-β-actin	Sigma-Aldrich	A5441<br>RRID: AB_476744
Rat anti-CD102	BD Biosciences	553326
Rat anti-CD31	Pharmigen	553370<br>RRID: AB_394816
Rat anti-VEGFR3	Thermo Fisher Scientific	14-5988-82<br>RRID:AB_467795
**Chemicals, Peptides, and Recombinant Proteins**		
[6-^{3}H]thymidine	Perkin Elmer	NET355L005MC
2-deoxy-D-glucose	Sigma Aldrich	D-6134
Antimycin A	Sigma-Aldrich	A8674
BMS-303141 (ATP citrate lyase inhibitor)	Sigma-Aldrich	SML0784
Chloroquine	Sigma-Aldrich	C6628
Etomoxir	Sigma-Aldrich	E1905
Glucose	Sigma-Aldrich	8769
Glutamine	Sigma-Aldrich	G7513
L-Carnitine	Sigma-Aldrich	8400920025
Methylcellulose	Sigma-Aldrich	M6385
Mitomycin C	Sigma-Aldrich	M4287
Oligomycin	Sigma-Aldrich	75351
Palmitic acid N-hydroxysuccinimide ester	Sigma-Aldrich	14464-31-4
Palmitic acid-$^{13}C_{16}$	Sigma-Aldrich	605573
Phenylhydrazone (FCCP)	Sanbio	1528-10
Propidium iodide	Sigma-Aldrich	P4170
Recombinant Mouse VEGF	R&D systems	493-MV-025

Saponin	Sigma-Aldrich	S-4521
Sodium acetate	Sigma-Aldrich	S2889
Sodium Pyruvate	Thermo Fisher Scientific	S8636
Tamoxifen	Sigma-Aldrich	T5648
Trichloroacetic acid	Sigma-Aldrich	T6399
VEGF-C human recombinant	Sigma-Aldrich	SRP3184
Critical Commercial Assays		
ORA qPCR Green L mix	HighQu	QPD0105
Reverse transcription kit	Qiagen	205313
RNeasy Plus mini kit	Qiagen	74136
Deposited Data		
Raw and analyzed data	This paper	
Experimental Models: Cell Lines		
Human dermal lymphatic endothelial cells	Promo Cell	C-12217
Experimental Models: Organisms/Strains		
Mouse: Atg5$^{fl/fl}$	Kuma et al. 2004	N/A
Mouse: Prox1-cre^{ERT2}	Bazigou et al. 2011	N/A
Oligonucleotides		
ON-TARGETplus Non-targeting Pool	Dharmacon	D-001810-10-20
SMARTpool: ON-TARGETplus ATG5 siRNA	Dharmacon	L-004374-00-0020
SMARTpool: ON-TARGETplus ATG7 siRNA	Dharmacon	L-020112-00-0050
SMARTpool: ON-TARGETplus DRP1 siRNA	Dharmacon	L-012092-00-0005
SMARTpool: ON-TARGETplus ULK1 siRNA	Dharmacon	L-005049-00-0005
Recombinant DNA		
pBABEpuro GFP-LC3 plasmid	Addgene	#22405
mCherry-hLC3B-pcDNA3.1	Addgene	#40827
Software and Algorithms		
Fiji (Image J)	Open Source	https://fiji.sc
FlowJo 8.8.6 software	FlowJo, LLC	https://www.flowjo.com
Image Lab Software	Licor	https://www.licor.com/bio/image-studio/?gclid=EAIaIQobChMIrP3L7_uS6wIVGed3Ch1uCw9yEAAYASAAEgJimPD_BwE
Prism v9	Graphpad	http://www.graphpad.com
Seahorse Wave Desktop Software	Agilent	https://www.agilent.com/?gclid=EAIaIQobChMIo5rHq-qS6wIVTe7tCh0YYw7gEAAYASAAEgJd1vD_BwE&gclsrc=aw.ds
FCS express V7	De Novo Software).	https://denovosoftware.com
MATLAB 8.3 (R2014a) software	Mathworks	https://www.mathworks.com/products/matlab.html
Leica MetaMorph AF2.1 morphometric software	Leica	https://www.moleculardevices.com/products/cellular-imaging-systems/acquisition-and-analysis-software/metamorph-microscopy
Other		
2x Antibiotic-Antimycotic	Sigma Aldrich	A5955
4% Paraformaldehyde	Thermo Fisher Scientific	J19943K2
4',6-Diamidino-2-Phenylindole, Dihydrochloride	Thermo Fisher Scientific	D1306

BODIPY 493/503	Thermo Fisher Scientific	D3922
BODIPY™ FL C_{16}	Thermo Fisher Scientific	D3821
Bovine serum albumin	Sigma-Aldrich	A6003-25G
CD31 magnetic beads	Miltenyi Biotec	130-097-418
CellVue™ Jade	Thermo Fisher Scientific	88-0876-16
CellVue™ NIR780	Thermo Fisher Scientific	88-0875-16
Charcoal stripped serum	Thermo Fischer Scientific	A3382101
Chemically Defined Lipid Concentrate	Thermo Fisher Scientific	11905031
Collagen gel (rat tail)	Merck	08-115
collagenase II	Thermo Fisher Scientific	17101015
collagenase IV	Thermo Fisher Scientific	17104019
DNase	Sigma Aldrich	D4527-10KU
ECGMV2	Promo Cell	C-22211
ECGMV2 Supplement Mix	Promo Cell	C-39226
FACS buffer	BD Biosciences	554656
FC block	Biolegend	101319
Fixable Viability Dye eFluor™ 780	Thermo Fisher Scientific	65-0865-14
Gelatin from bovine skin	Sigma-Aldrich	G9391
Gentle Macs C Tubes	Miltenyi Biotec	130-093-237
Goat serum	Cell Signaling Technology	#5425
Hepes	Gibco	12509079
Ketamine hydrochloride	Dechra	
KnockOut™ DMEM	Thermo Fisher Scientific	10829018
Lipofectamine 2000	Invitrogen	16688
Magnetic beads CELLection	Invitrogen	11533D
MitoSOX™ Red Mitochondrial Superoxide Indicator	Thermo Fischer Scientific	M36008
MitoTracker Green	Thermo Fischer Scientific	M7514
MitoTracker Deep Red	Thermo Fisher Scientific	M22426
Nile Red	Thermo Fisher Scientific	N1142
Non-Essential Amino Acids Solution	Sigma Aldrich	M7145
penicillin/streptomycin	Sigma Aldrich	P4333
Prolong® Gold Antifade Reagent	Thermo Fisher Scientific	P36934
Seahorse XF Assay Medium	Agilent	103335-100
Seahorse Xp culture plates	Agilent	103022-100
Sodium pyruvate	Sigma Aldrich	S8636
Supplement Mix 2	Promo Cell	C-39216
Supplement Mix MV2	Promo Cell	C-39226
TMRM	Thermo Fischer Scientific	T-668
Unicaïne	Thea	048720
Xylazine	VMD	

Gating strategies Supplementary Fig. 3i

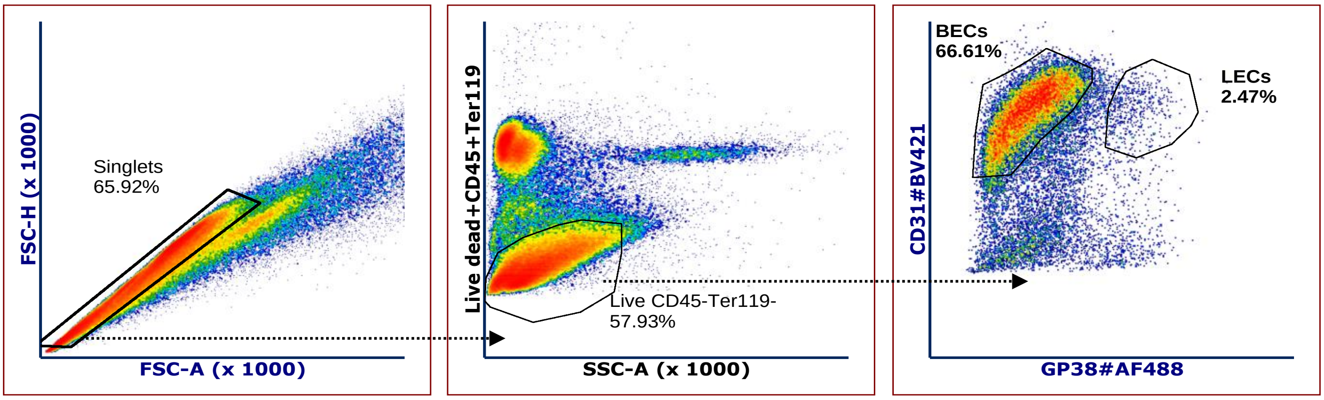

Endothelial cells were isolated from mice lungs as described in the method section. Single cells were gated based on FSC-A and FSC-H (first panel). Cells were further gated based on CD45, Ter119 and Live dead triple negative populations (middle panel). Lymphatic endothelial cells were further identified as CD31 and GP38 double positive cells (last panel).
