# Supplementary material for: Lipid droplet degradation by autophagy connects mitochondria metabolism to Prox1-driven expression of lymphatic genes and lymphangiogenesis
Source: Nat Commun. 2022 May 19;13:2760. doi: 10.1038/s41467-022-30490-6 (PMC9120506; doi:10.1038/s41467-022-30490-6)
Supplement: Supplementary file 2 — Reporting Summary [file 41467_2022_30490_MOESM2_ESM.pdf]

nature research

Corresponding author(s): Patrizia Agostinis

Last updated by author(s): Apr 7, 2022

Reporting Summary

Nature Research wishes to improve the reproducibility of the work that we publish. This form provides structure for consistency and transparency in reporting. For further information on Nature Research policies, see our Editorial Policies and the Editorial Policy Checklist.

Statistics

For all statistical analyses, confirm that the following items are present in the figure legend, table legend, main text, or Methods section.

n/a	Confirmed	
☐	☒	The exact sample size (*n*) for each experimental group/condition, given as a discrete number and unit of measurement
☐	☒	A statement on whether measurements were taken from distinct samples or whether the same sample was measured repeatedly
☐	☒	The statistical test(s) used AND whether they are one- or two-sided<br>*Only common tests should be described solely by name; describe more complex techniques in the Methods section.*
☒	☐	A description of all covariates tested
☐	☒	A description of any assumptions or corrections, such as tests of normality and adjustment for multiple comparisons
☐	☒	A full description of the statistical parameters including central tendency (e.g. means) or other basic estimates (e.g. regression coefficient) AND variation (e.g. standard deviation) or associated estimates of uncertainty (e.g. confidence intervals)
☐	☒	For null hypothesis testing, the test statistic (e.g. *F*, *t*, *r*) with confidence intervals, effect sizes, degrees of freedom and *P* value noted<br>*Give P values as exact values whenever suitable.*
☒	☐	For Bayesian analysis, information on the choice of priors and Markov chain Monte Carlo settings
☒	☐	For hierarchical and complex designs, identification of the appropriate level for tests and full reporting of outcomes
☒	☐	Estimates of effect sizes (e.g. Cohen's *d*, Pearson's *r*), indicating how they were calculated

Our web collection on statistics for biologists contains articles on many of the points above.

Software and code

Policy information about availability of computer code

Data collection	QuantaSmart TM PerkinElmer (scintillation counting), JEOL JEM1400 (Transmission electron microscope), Leica DMI6000 (cornea imaging), Leica sp8x confocal microscope, Zeiss LSM780, Zeiss LSM 880 – Airyscan, AttuneTM Cytometer (flow cytometry), IX73, Olympus (inverted microscope),Nikon C2 Eclipse Ni-E (inverted confocal microscope),Bio-Rad Chemidoc Imager ,Bio-Rad Laboratories N.V.3 (Western-Blot Imager), XFp Analyzer (Seahorse flux analyzer), HILIC LC-MS/MS, Lipometrix (lipidomics analysis), BD FACSymphony A5 instrument, ABI 7,500 machine (RT-qPCR analysis)
Data analysis	ImageJ/FIJI 1.50i was used for image analysis, FlowJo 8.8.6 or FCS express V7 software was used for flow cytometric analysis, GraphPad Prism V9 was used for statistical analysis, Seahorse Wave Desktop SoftwareV2.6 was used for seahorse analysis, MATLAB 8.3 (R2014a) software was used for analysis of corneal (lymph)angiogenesis and Leica MetaMorph AF2.1 morphometric software for EM analysis

For manuscripts utilizing custom algorithms or software that are central to the research but not yet described in published literature, software must be made available to editors and reviewers. We strongly encourage code deposition in a community repository (e.g. GitHub). See the Nature Research guidelines for submitting code & software for further information.

Data

Policy information about availability of data

All manuscripts must include a data availability statement. This statement should provide the following information, where applicable:

- Accession codes, unique identifiers, or web links for publicly available datasets
- A list of figures that have associated raw data
- A description of any restrictions on data availability

All data presented in this study are included in the article. No data are deposited in external repositories. A reporting summary for this article is available as a Supplementary Information file.

Field-specific reporting

Please select the one below that is the best fit for your research. If you are not sure, read the appropriate sections before making your selection.

☒ Life sciences ☐ Behavioural & social sciences ☐ Ecological, evolutionary & environmental sciences

For a reference copy of the document with all sections, see nature.com/documents/nr-reporting-summary-flat.pdf

Life sciences study design

All studies must disclose on these points even when the disclosure is negative.

Sample size	For all in-vitro and in-vivo analysis in this study, we choose the sample size based on literature in the field: Wong et al 2017, García-Caballero et al 2019, Maes et al 2014. For all in vitro analyses we performed a minimum of 3 independent biological replicates ; for in vivo experiments a minimum of 5 mice were analyzed for each group (the exact number of animals is presented by data points in each graph).
Data exclusions	No data was excluded in any of the in vivo or in vitro experiments .
Replication	All data presented in this article represent independent biological experiments, also stated in figure legends.
Randomization	No formal randomization techniques were used; however, samples were allocated randomly to experiments and processed in an arbitrary order. We tried to keep stable conditions while running experiments to avoid variability between experiments performed in different times.
Blinding	The investigators analyzing the data were blinded to experimental groups/treatments assignment.

Reporting for specific materials, systems and methods

We require information from authors about some types of materials, experimental systems and methods used in many studies. Here, indicate whether each material, system or method listed is relevant to your study. If you are not sure if a list item applies to your research, read the appropriate section before selecting a response.

Materials & experimental systems

n/a	Involved in the study	
☐	☒	Antibodies
☐	☒	Eukaryotic cell lines
☒	☐	Palaeontology and archaeology
☐	☒	Animals and other organisms
☐	☒	Human research participants
☒	☐	Clinical data
☒	☐	Dual use research of concern

Methods

n/a	Involved in the study	
☒	☐	ChIP-seq
☐	☒	Flow cytometry
☒	☐	MRI-based neuroimaging

Antibodies

Antibodies used

Anti Goat IgG HRP-linked antibody (Thermo Fisher Scientific, PA1-28823, RRID:AB_10986856, dilution 1:2000)
Anti-Goat IgG, Alexa Fluor 488 linked antibody (Molecular Probes, A21222, RRID: AB_10373853, dilution 1:2000)
Anti-Mouse IgG HRP-linked antibody (Cell Signaling Technology, 7076, RRID: AB_330924, dilution 1:2000)
Anti-Rabbit IgG HRP-linked antibody (Cell Signaling Technology, 7074, RRID:AB_2099233, dilution 1:2000)

Goat anti-LYVE1 (R&D systems, AF2089, RRID: AB_355144, dilution 1:1000)
Species Reactivity: Human, Application validated by manufacturer: Detects human LYVE-1 in direct ELISAs and Western blots

Goat anti-LYVE1 (R&D systems, AF2125, RRID: AB_2297188, dilution 1:100)
Species Reactivity: Mouse, Application validated by manufacturer: Detects human LYVE-1 in direct ELISAs and Western blots

Rat anti-VEGF Receptor 3 (eBioscience, Catalog # 14-5988-82, RRID:AB_467795, dilution 1:100)
Species reactivity: Mouse, Application validated by the manufacturer: Western blots, IHC-P, IHC-F.

Goat anti-Mouse IgG, Alexa Fluor 647 antibody (Thermo Fisher Scientific, A21235, RRID: AB_2535804, dilution 1:200)

Rabbit anti-CD36 (Abcam, ab133625, RRID:AB_2716564), dilution 1:1000)
Species reactivity: Human, Application validated by manufacturer: Western blots, IHC-P

Mouse anti-DRP1 (BD Biosciences, 611113, RRID:AB_398424, dilution 1:1000)
Reactivity: Mouse, Human, Dog, Application validated by manufacturer: Western blots, Immunofluorescence

Mouse anti-NR2F2 (Abcam, ab41859, dilution 1:1000)
Reactivity: Mouse, Rat, Human, Application validated by manufacturer: IP, ELISA, IHC-P, WB, ICC/IF

Mouse anti-OPA1 (BD Biosciences, 612607, RRID: AB_399889, dilution 1:1000)
Reactivity: Dog, Chicken, Human, Mouse, Rat, Application validated by manufacturer: Western blots, Immunofluorescence

Mouse anti-TOMM20 (BD Biosciences, BD 612278, RRID:AB_399595, dilution 1:1000)
Reactivity:Dog, Human, Rat, Application validated by manufacturer: Western blots, Immunofluorescence

Rabbit anti-ULK1 (Cell Signaling, 8054s, Cat# 8054, RRID:AB_11178668 dilution 1:1000)
Reactivity:Human, Mouse, Rat, Monkey, Application validated by manufacturer: Western Blot, Immunoprecipitation, Immunohistochemistry, Chromatin Immunoprecipitation, Immunofluorescence, Flow Cytometry, ELISA-Peptide

Rabbit anti- Phospho-DRP1 (Ser616) (Cell Signaling Technology, 3455S, RRID:AB_2085352, dilution 1:1000)
Reactivity: Human, Application validated by manufacturer: Western Blot, Immunoprecipitation, Immunohistochemistry, Chromatin Immunoprecipitation, Immunofluorescence, Flow Cytometry, ELISA-Peptide

Rabbit anti-acetyl histone H3 (lysine 9) (Cell Signaling Technology, 9671, RRID: AB_331532, dilution 1:1000)
Reactivity: Human, Mouse, Rat, Monkey, D. melanogaster, S. cerevisiae Application validated by manufacturer: Western Blot, Immunoprecipitation, Immunohistochemistry, Chromatin Immunoprecipitation, Immunofluorescence, Flow Cytometry, ELISA-Peptide

Rabbit anti-ATG5 (Cell Signaling Technology, 12994S, RRID: AB_2630393, dilution 1:100 for tissue staining, 1:1000 for WB)
Reactivity: Human, Mouse, Rat. Application validated by manufacturer: Western Blot, Immunoprecipitation, Immunohistochemistry, Chromatin Immunoprecipitation, Immunofluorescence, Flow Cytometry, ELISA-Peptide

Rabbit anti-ATG7 (Cell Signaling Technology, 8558S, RRID:AB_10831194, dilution 1:1000)
Reactivity: Human, Mouse, Rabbit. Application validated by manufacturer:Western Blot, Immunoprecipitation

Rabbit anti-CPT1 (Cell Signaling Technology, D3B3 12252, RRID: AB_2797857, dilution 1:1000)
Reactivity:Human, Application validated by manufacturer:Western Blot, Immunoprecipitation

Rabbit anti-CPT2 (Abcam, ab181114, RRID:AB_2687503 dilution 1:1000)
Reactivity: Mouse, Rat, Human. Application validated by manufacturer:Western Blot, Immunoprecipitation,Immunohistochemistry,Immunofluorescence

Rabbit anti-ENOS (BD Biosciences, 610297, RRID: AB_397691, dilution 1:1000)
Reactivity: Human, Mouse, Rat. Application validated by manufacturer: Western Blot, Immunoprecipitation, Immunohistochemistry, Immunofluorescence

Rabbit anti-FABP4 (Cell Signaling Technology, 2120S, RRID: AB_2102466, dilution 1:1000)
Reactivity: Human, Mouse, Application validated by manufacturer: Western Blot

Rabbit anti-GAPDH (Cell Signaling Technology, 2118S, RRID: AB_561053, dilution 1:5000)
Reactivity: Human, Mouse, Rabbit, Monkey, Bovine, Pig. Application validated by manufacturer: Western Blot, Immunohistochemistry, Immunofluorescence, Flow Cytometry

Rabbit anti-LC3 (Cell Signaling Technology, 3868S, RRID: AB_2137707, dilution1: 100 for tissue staining and 1:1000 for WB)
Reactivity: Human. Species predicted to react based on 100% sequence homology:
Mouse, Rat, Monkey, Bovine, Pig. Application validated by manufacturer: Western Blot, Immunofluorescence, Flow Cytometry

Rabbit anti-p62 (Millipore, p0067, RRID: AB_1841064, dilution 1:1000)
Reactivity: Human, Mouse, Rat. Application validated by manufacturer: Western Blot, Immunohistochemistry

Rabbit anti-pan-acetyl-histone H3 (Active Motif, 39139, RRID: AB_2687871, dilution 1:1000)
Reactivity: Human, Application validated by manufacturer:ChiP, ChiP-Sec, Western Blotting

Rabbit anti-PROX1 (Proteintech, 11067-2, RRID: AB_2268804, dilution 1:1000)
Reactivity: Human, Mouse, Rat. Application validated by manufacturer: Western Blot, Immunohistochemistry, ChiP, ELISA

Rabbit anti-VEGFR3 (Abcam, ab154079, dilution 1:1000)
Reactivity: Human. Application validated by manufacturer: Western Blot

Rabbit anti-Vinculin (Cell Signaling Technology, #4650, RRID:AB_10559207, dilution 1:1000)
Reactivity: Human, Mouse, Rat, Monkey, Dog. Application validated by manufacturer:Western Blot

Rabbit anti-β-actin (Sigma-Aldrich, A5441, RRID: AB_476744, dilution 1:5000)
Reactivity: Human, Bovine, Sheep, Pig, Rabbit, Cat, Dog, Mouse, Rat, Guinea pig, Chicken, Carp, and leech tissues. Application validated by manufacturer:Western Blot, Immunohistochemistry, Immunofluorescence

Rat anti-CD102 (BD Biosciences, 553326, RRID: AB_394784, dilution 1:50)
Reactivity: Mouse. Application validated by manufacturer: Blocking experiments, Flow cytometry/Cell sorting, Immunohistochemistry-frozen tissue, Immunohistochemistry-paraffin, Immunoprecipitation

Rat anti-CD31 (Pharmigen, 553370, RRID: AB_394816, dilution 1:100)
Reactivity: Mouse. Application validated by manufacturer: Blocking experiments, Flow cytometry/Cell sorting,

Immunohistochemistry-frozen tissue, Immunohistochemistry-paraffin, Immunoprecipitation

Rabbit anti- Phospho-DRP1 (Ser616) (Cell Signaling Technology, 3455S, RRID:AB_2085352, dilution 1:1000)
Reactivity: Human. Application validated by manufacturer: Western Blot, Immunoprecipitation, Immunofluorescence, Flow Cytometry

Rabbit anti-TOMM22 (My Biosource MBS7605092, dilution 1:100)
Reactivity: Human. Application validated by manufacturer: ELISA, Western Blot, Immunohistochemistry

Mouse anti-LAMP1 (Abcam ab25630, dilution 1:100)
Reactivity: Human, Rat, Green Monkey. Application validated by manufacturer: Flow cytometry/Cell sorting, Immunocytochemistry-immunoflourescence , Immunohistochemistry, Immunohistochemistry-paraffin ,Western Blotting

anti-GP38 (Biolegend 156207, RRID:AB_2814079, dilution 1:200)
Reactivity: Mouse. Application validated by manufacturer: Flow Cytometry

anti-CD31 (BD Biosciences 563356, dilution 1:200)
Reactivity: Mouse. Application validated by manufacturer:Flow cytometry/Cell sorting

anti-CD36 (Biolegend 102605, RRID:AB_389348, dilution 1:100)
Reactivity: Mouse. Application validated by manufacturer:Flow cytometry

anti-CD45 (BD Biosciences 557659, dilution 1:200)
Reactivity: Mouse. Application validated by manufacturer:Flow cytometry

Anti-Mouse IgG, Alexa Fluor 488 (Thermo Fisher Scientific, A11001, RRID:AB_2534069, dilution 1:200)
Anti-Mouse IgG, Alexa Fluor 647 (Thermo Fisher Scientific A21235, RRID:AB_2535804, dilution 1:200)
Anti-Rabbit IgG, Alexa Fluor 488 (Thermo Fisher Scientific A21206, RRID:AB_2535792, dilution 1:200)
Anti-Rabbit IgG, Alexa Fluor 647 (Thermo Fisher Scientific A21222, RRID:AB_2535812244, dilution 1:200)
Anti-Rat IgG, Alexa Fluor 546 (Thermo Fisher Scientific A11081, RRID:AB_141738, dilution 1:200)

anti-ter119 (BD Biosciences 560509, dilution 1:100)
Reactivity: Mouse. Application validated by manufacturer:Flow cytometry

Goat anti-Mouse IgG, Alexa Fluor 647 (Thermo Fisher Scientific A21235, RRID: AB_2535804)

Hamster anti-PDPN (Biolegend 127401, RRID: AB_1089186, dilution 1:100)
Reactivity: Mouse. Application validated by manufacturer:Flow cytometry, Immunocytochemistry

Validation

Validation: All antibodies used in this manuscript were commercially bought and validated by the manufacturers, as mentioned on their website. All antibodies used to detected the respective protein at the expected molecular weight, subcellular localization or tissue expression pattern.

Eukaryotic cell lines

Policy information about cell lines

Cell line source(s)	Human dermal lymphatic endothelial cells (LEC) were commercially purchased from Promocell or Mice lung lymphatic endothelial cells were isolated as described in the methods section.
Authentication	Only primary endothelial cells were used and no cell lines.
Mycoplasma contamination	Cells were regularly checked for mycoplasma contamination and used only when testing mycoplasma negative.
Commonly misidentified lines (See ICLAC register)	No misidentified cell lines were used in this study.

Animals and other organisms

Policy information about studies involving animals; ARRIVE guidelines recommended for reporting animal research

Laboratory animals	C57Bl/6, (Prox1-Cre+ERT2; Atg5fl/fl and Prox1-Cre-ERT2; Atg5fl/fl, male and female mice 8 to 12 weeks old.

Wild animals	This study did not involve wild animals
Field-collected samples	This study did not involve field collected samples.
Ethics oversight	All animal experiments were approved by the Ethical Committee for Animal Experimentation, Laboratory Animal Center KU Leuven, Belgium under the reference number LA1210202.

Note that full information on the approval of the study protocol must also be provided in the manuscript.

Human research participants

Policy information about studies involving human research participants

Population characteristics	*Describe the covariate-relevant population characteristics of the human research participants (e.g. age, gender, genotypic information, past and current diagnosis and treatment categories). If you filled out the behavioural & social sciences study design questions and have nothing to add here, write "See above."*
Recruitment	*Describe how participants were recruited. Outline any potential self-selection bias or other biases that may be present and how these are likely to impact results.*
Ethics oversight	*Identify the organization(s) that approved the study protocol.*

Note that full information on the approval of the study protocol must also be provided in the manuscript.

Flow Cytometry

Plots

Confirm that:

☒ The axis labels state the marker and fluorochrome used (e.g. CD4-FITC).

☒ The axis scales are clearly visible. Include numbers along axes only for bottom left plot of group (a 'group' is an analysis of identical markers).

☒ All plots are contour plots with outliers or pseudocolor plots.

☒ A numerical value for number of cells or percentage (with statistics) is provided.

Methodology

Sample preparation	LEC were detached and stained with corresponding fluorescent dyes (MitoTracker green, TMRM, MitoSox), mouse LEC were isolated from lung tissues, dissociated with gentle MACS dissociator system and magnetically enriched for CD31+ cells and then stained with corresponding antibodies.
Instrument	BD FACSymphony A5 instrument or AttuneTM Cytometer
Software	FlowJo 8.8.6 or FCS express V7 software
Cell population abundance	BEC 94.10%, LEC 2.86%
Gating strategy	FCS/SSC, FSC-A/FSC-H, viability, CD45-/ter119-, CD31/GP38

☒ Tick this box to confirm that a figure exemplifying the gating strategy is provided in the Supplementary Information.
