# Supplementary material for: Lipid droplet degradation by autophagy connects mitochondria metabolism to Prox1-driven expression of lymphatic genes and lymphangiogenesis
Source: Nat Commun. 2022 May 19;13:2760. doi: 10.1038/s41467-022-30490-6 (PMC9120506; doi:10.1038/s41467-022-30490-6)

Figure 1A

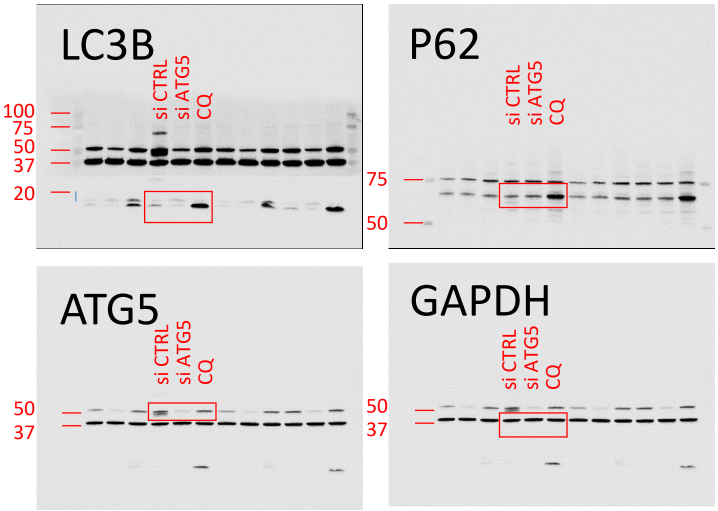

LC3B
si CTRL
si ATG5
CQ
100
75
50
37
20
P62
si CTRL
si ATG5
CQ
75
50
ATG5
si CTRL
si ATG5
CQ
50
37
GAPDH
si CTRL
si ATG5
CQ
50
37

Figure 2A

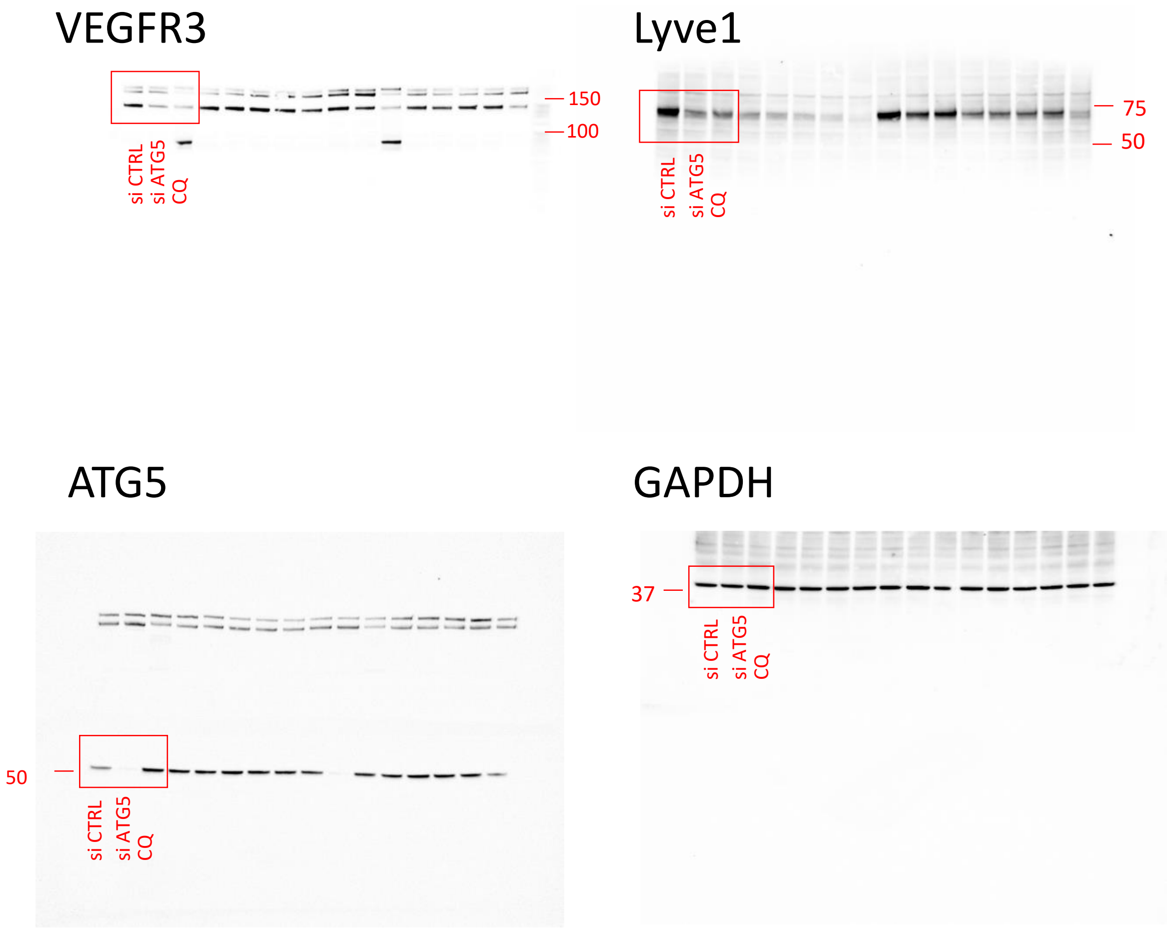
VEGFR3
150
100
si CTRL
si ATG5
CQ
Lyve1
75
50
si CTRL
si ATG5
CQ
ATG5
50
si CTRL
si ATG5
CQ
GAPDH
37
si CTRL
si ATG5
CQ

Figure 2B

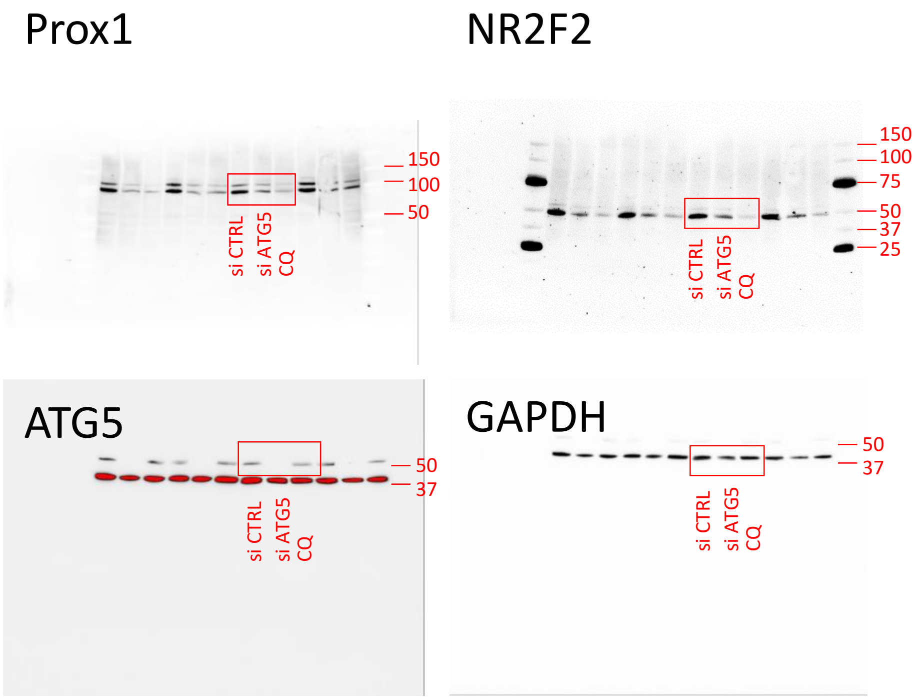
Prox1
150
100
50
si CTRL
si ATG5
CQ
NR2F2
150
100
75
50
37
25
si CTRL
si ATG5
CQ
ATG5
50
37
si CTRL
si ATG5
CQ
GAPDH
50
37
si CTRL
si ATG5
CQ

Figure 2C

First gel

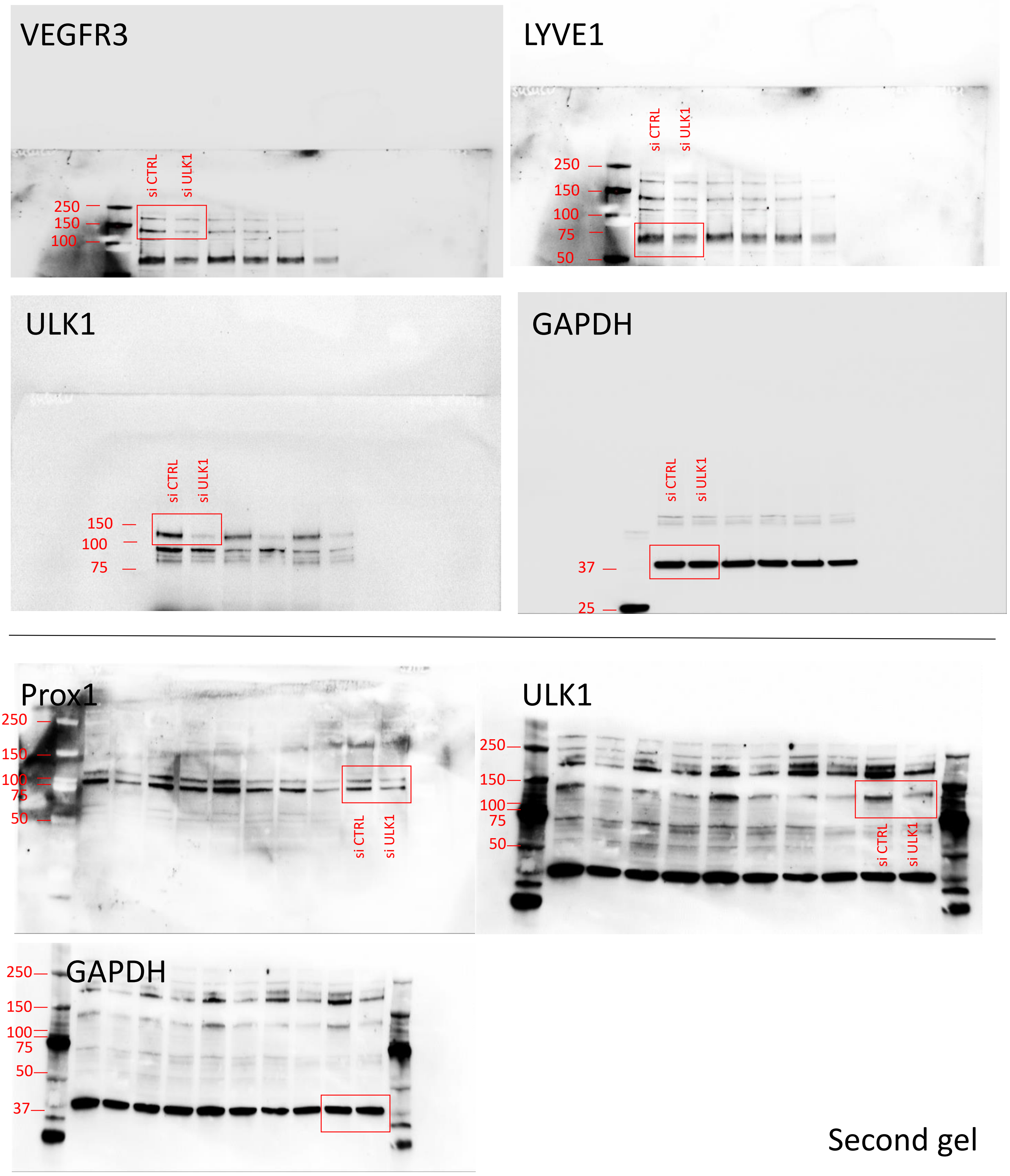

Second gel

Supplementary Figure 2A

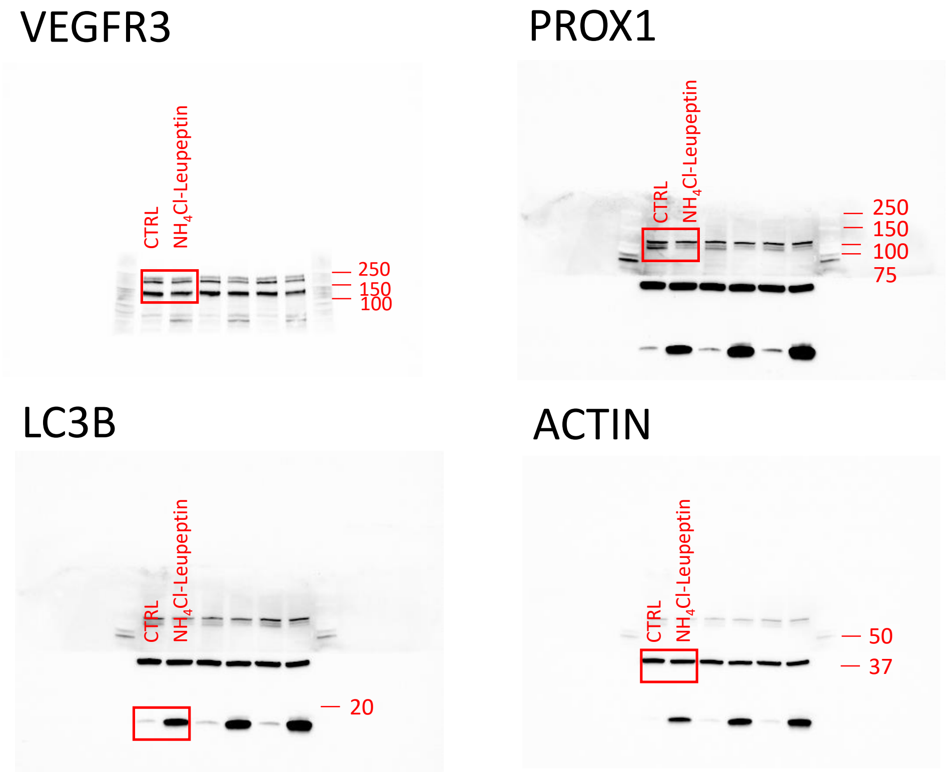

Supplementary Figure 2B

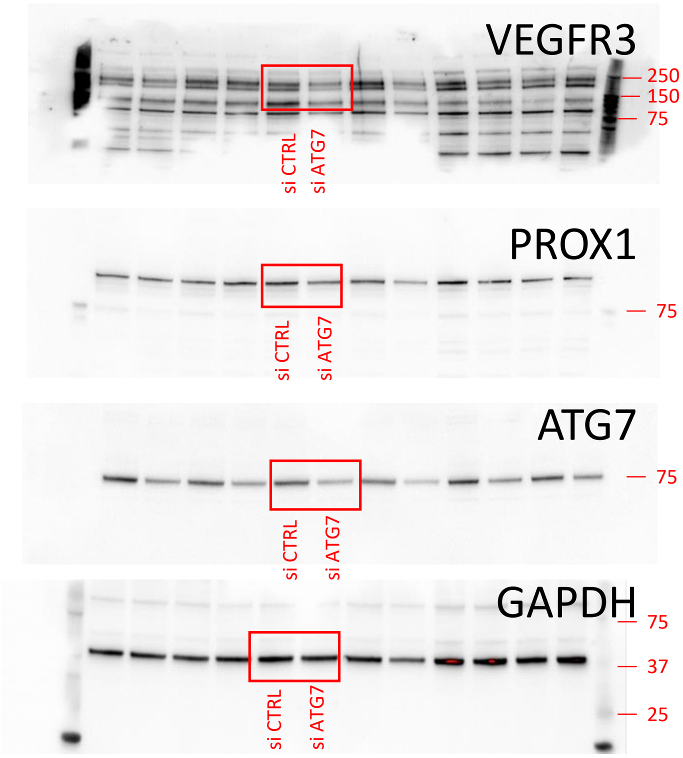

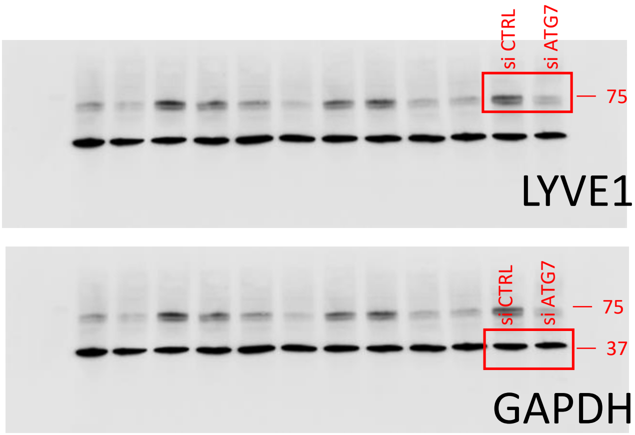

Supplementary Figure 3A

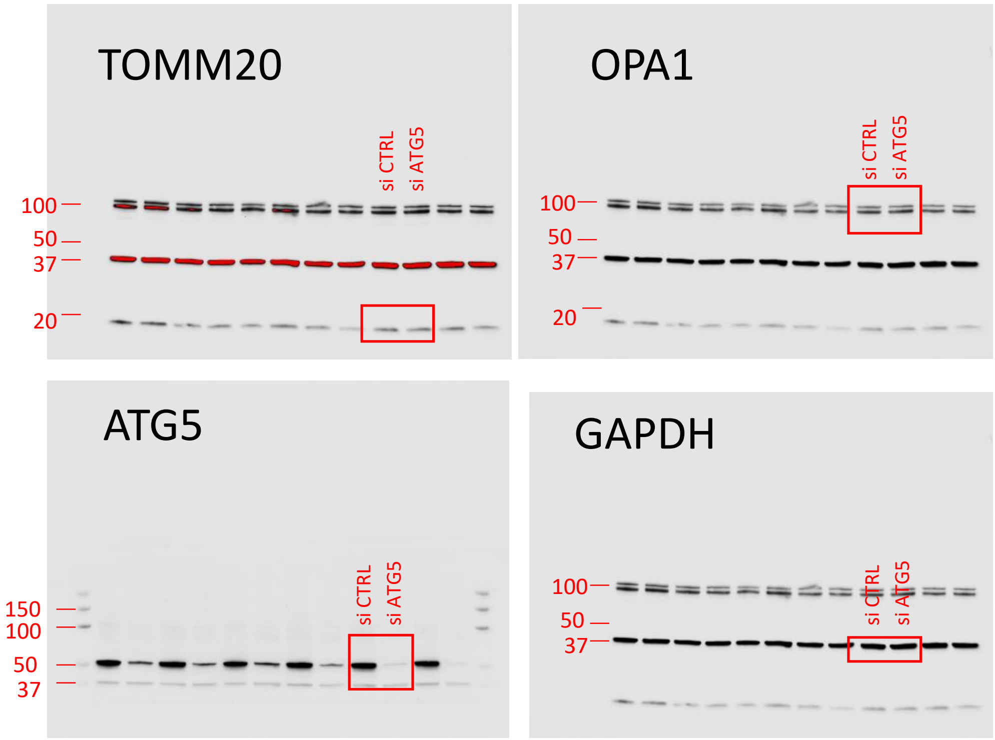

Supplementary Figure 3J

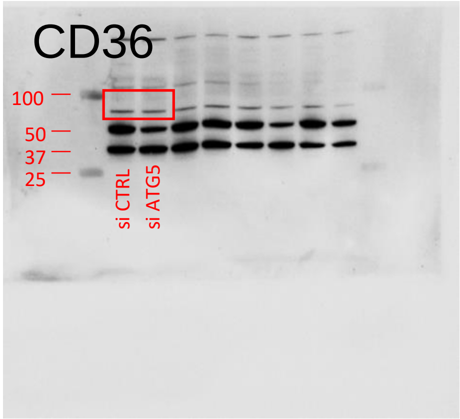

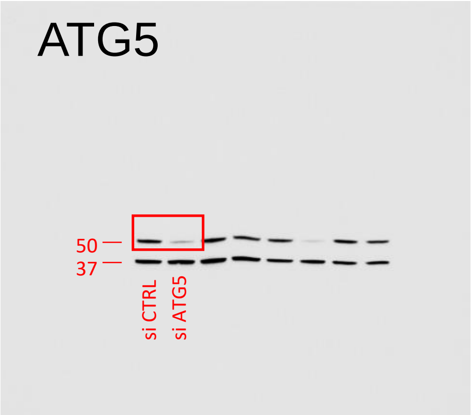

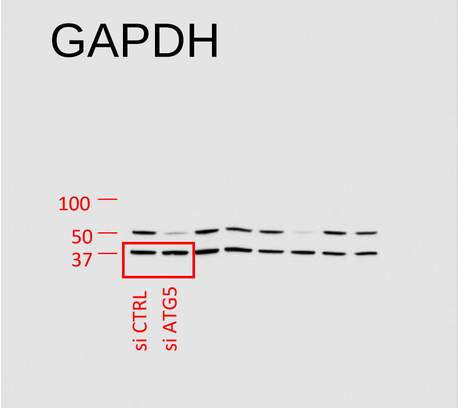

Supplementary Figure 3L

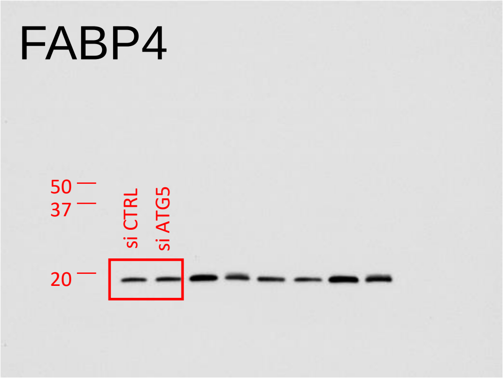

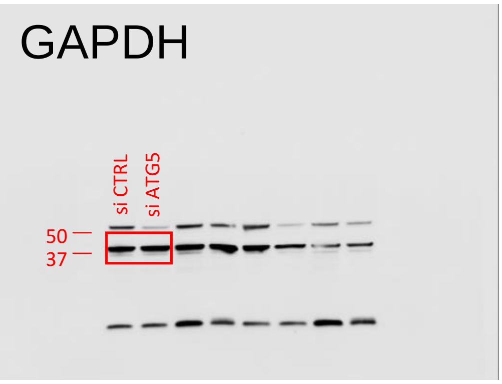

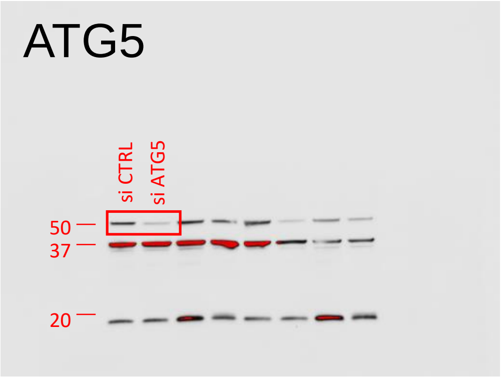

Supplementary Figure 4T

eNOS

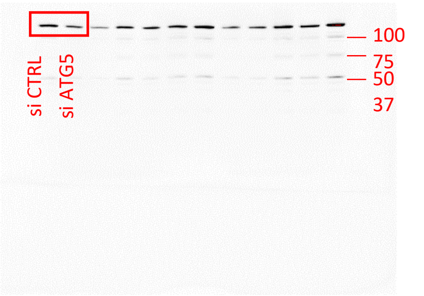

ATG5

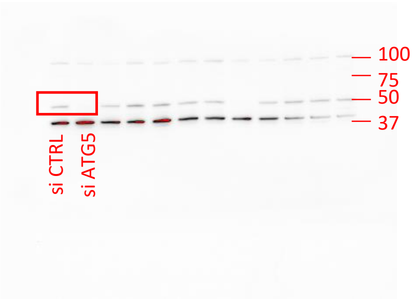

GAPDH

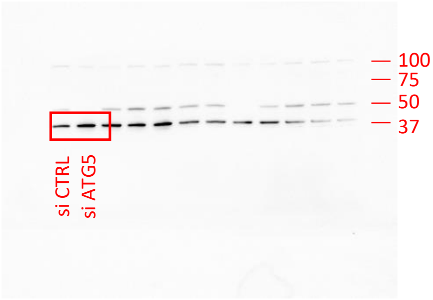

Figure 5A

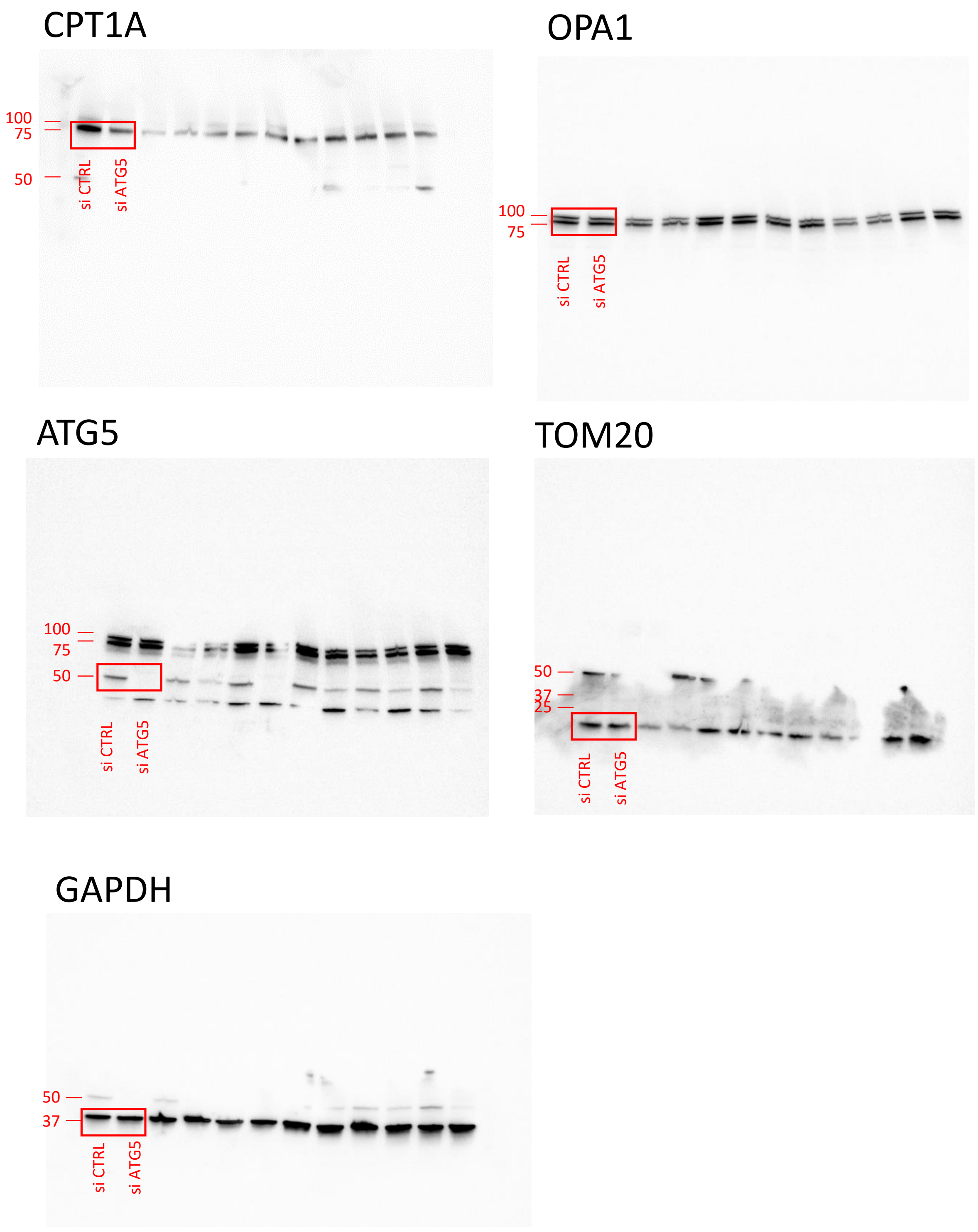

CPT1A
100
75
50
si CTRL
si ATG5
OPA1
100
75
si CTRL
si ATG5
ATG5
100
75
50
si CTRL
si ATG5
TOM20
50
37
25
si CTRL
si ATG5
GAPDH
50
37
si CTRL
si ATG5

Figure 5F

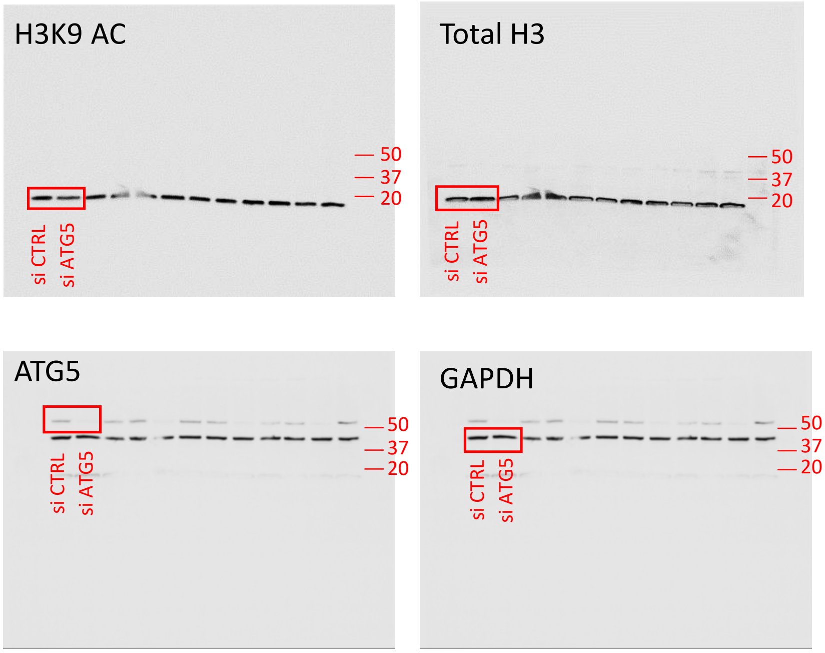
H3K9 AC
50
37
20
si CTRL
si ATG5
Total H3
50
37
20
si CTRL
si ATG5
ATG5
50
37
20
si CTRL
si ATG5
GAPDH
50
37
20
si CTRL
si ATG5

Figure 5H

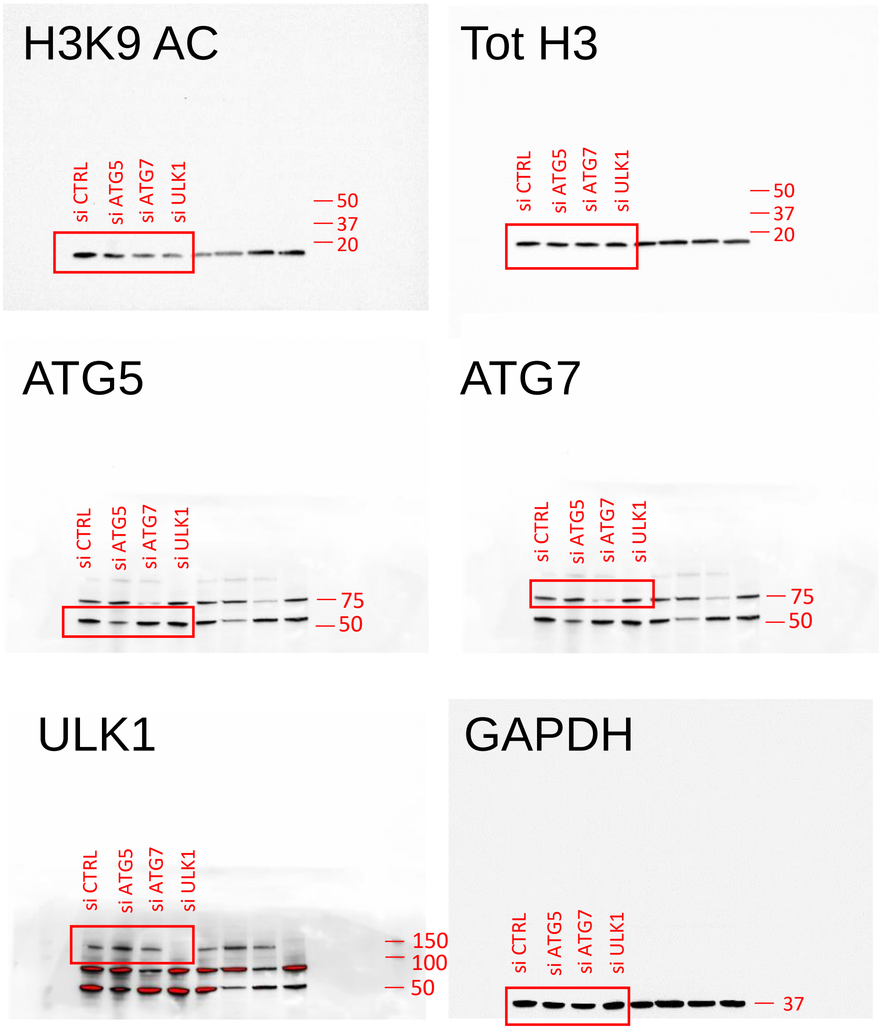
H3K9 AC
si CTRL
si ATG5
si ATG7
si ULK1
50
37
20
Tot H3
si CTRL
si ATG5
si ATG7
si ULK1
50
37
20
ATG5
si CTRL
si ATG5
si ATG7
si ULK1
75
50
ATG7
si CTRL
si ATG5
si ATG7
si ULK1
75
50
ULK1
si CTRL
si ATG5
si ATG7
si ULK1
150
100
50
GAPDH
si CTRL
si ATG5
si ATG7
si ULK1
37

Supplementary Figure 5A

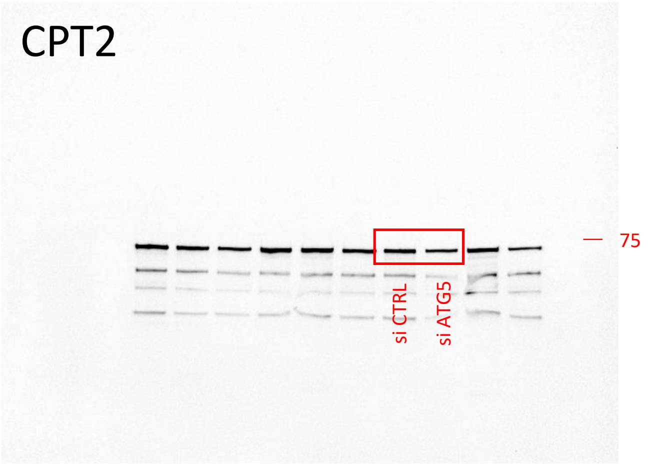

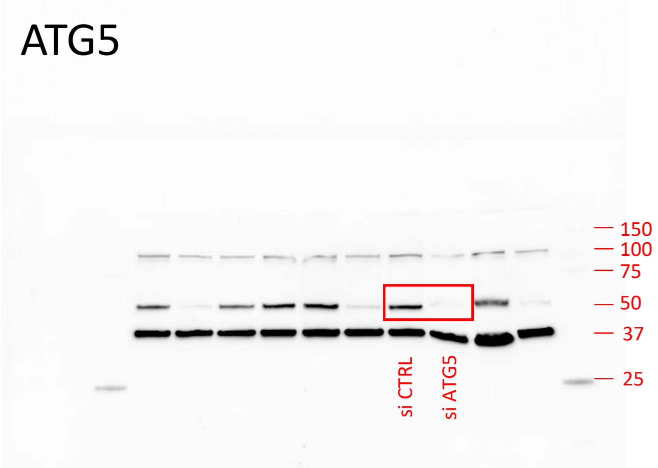

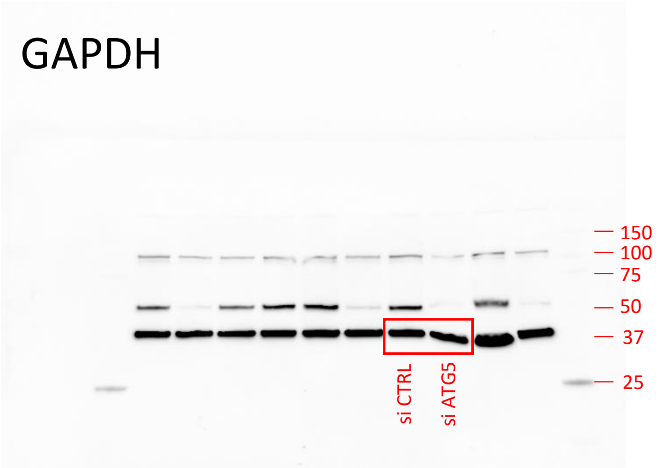

Supplementary Figure 5D

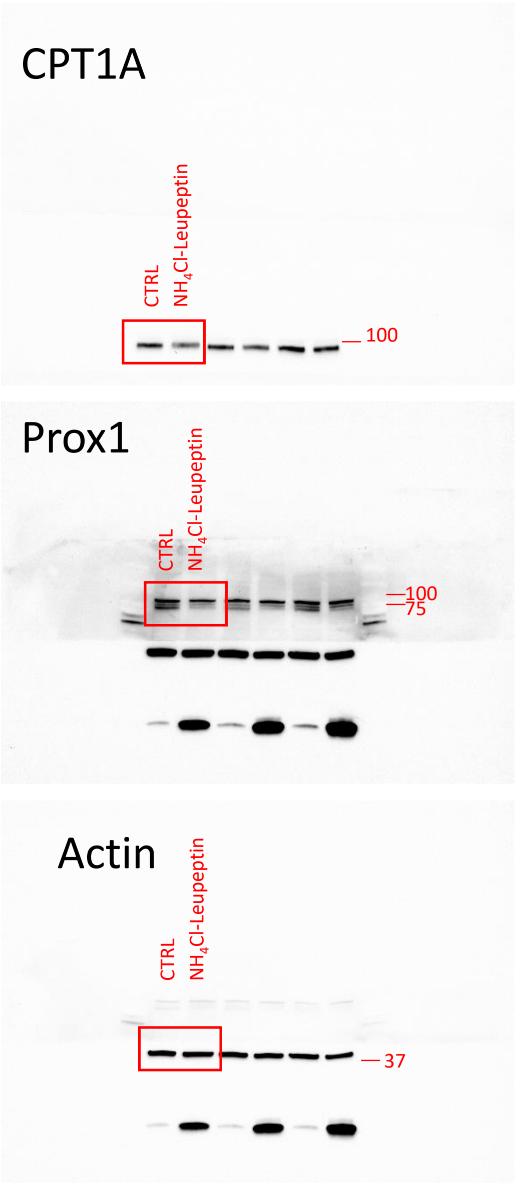

Supplementary Figure 5F

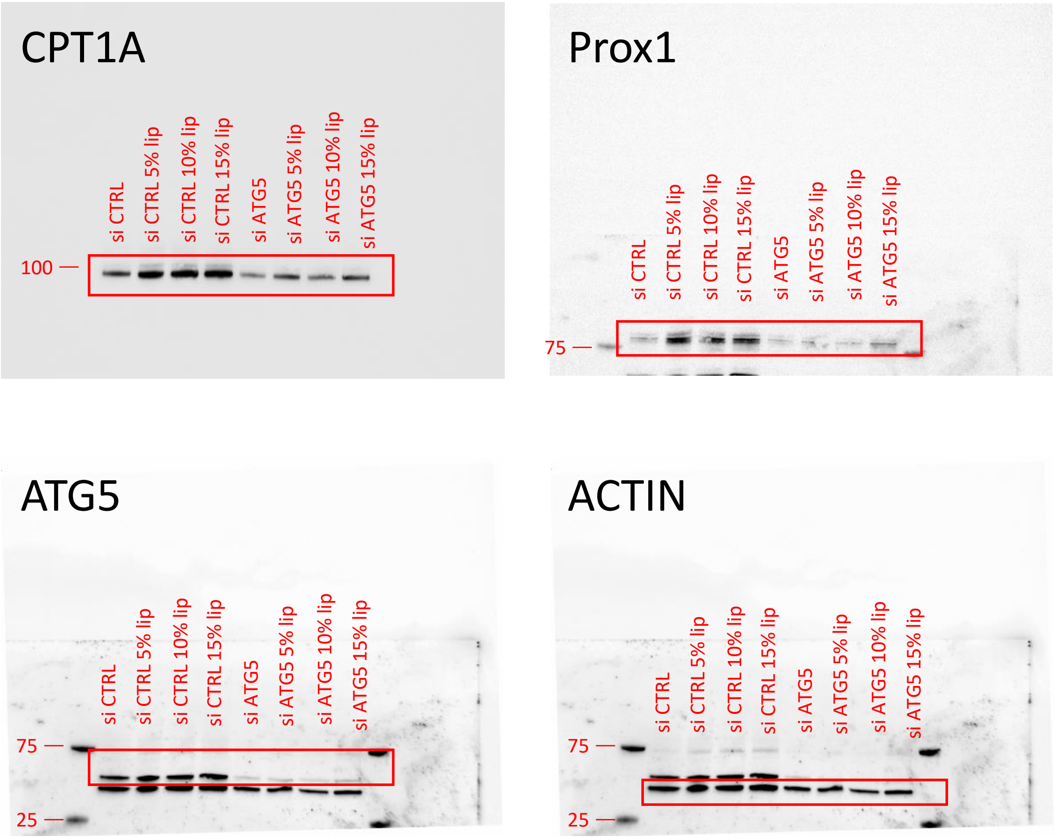

Supplementary Figure 5G

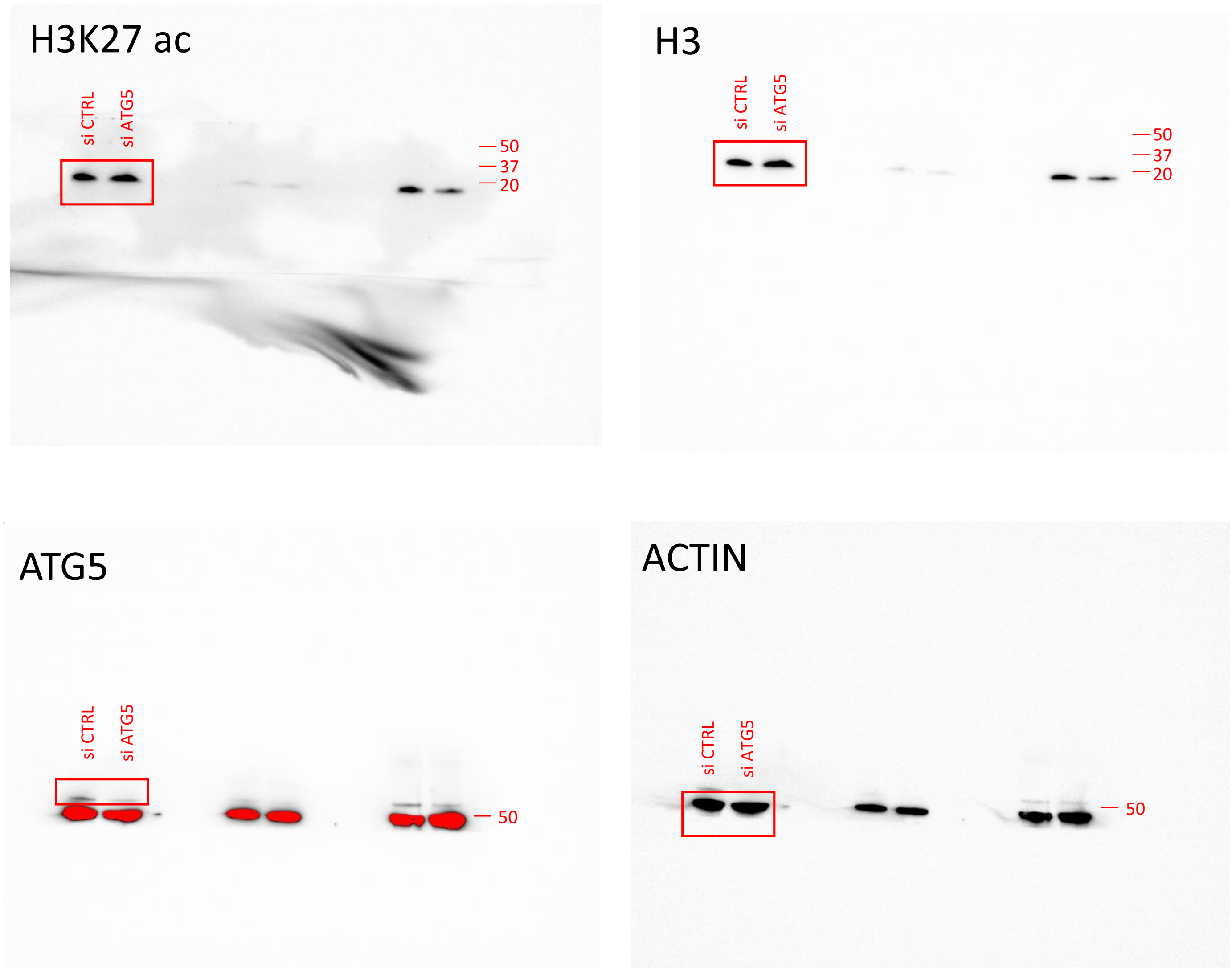

Supplementary 5H

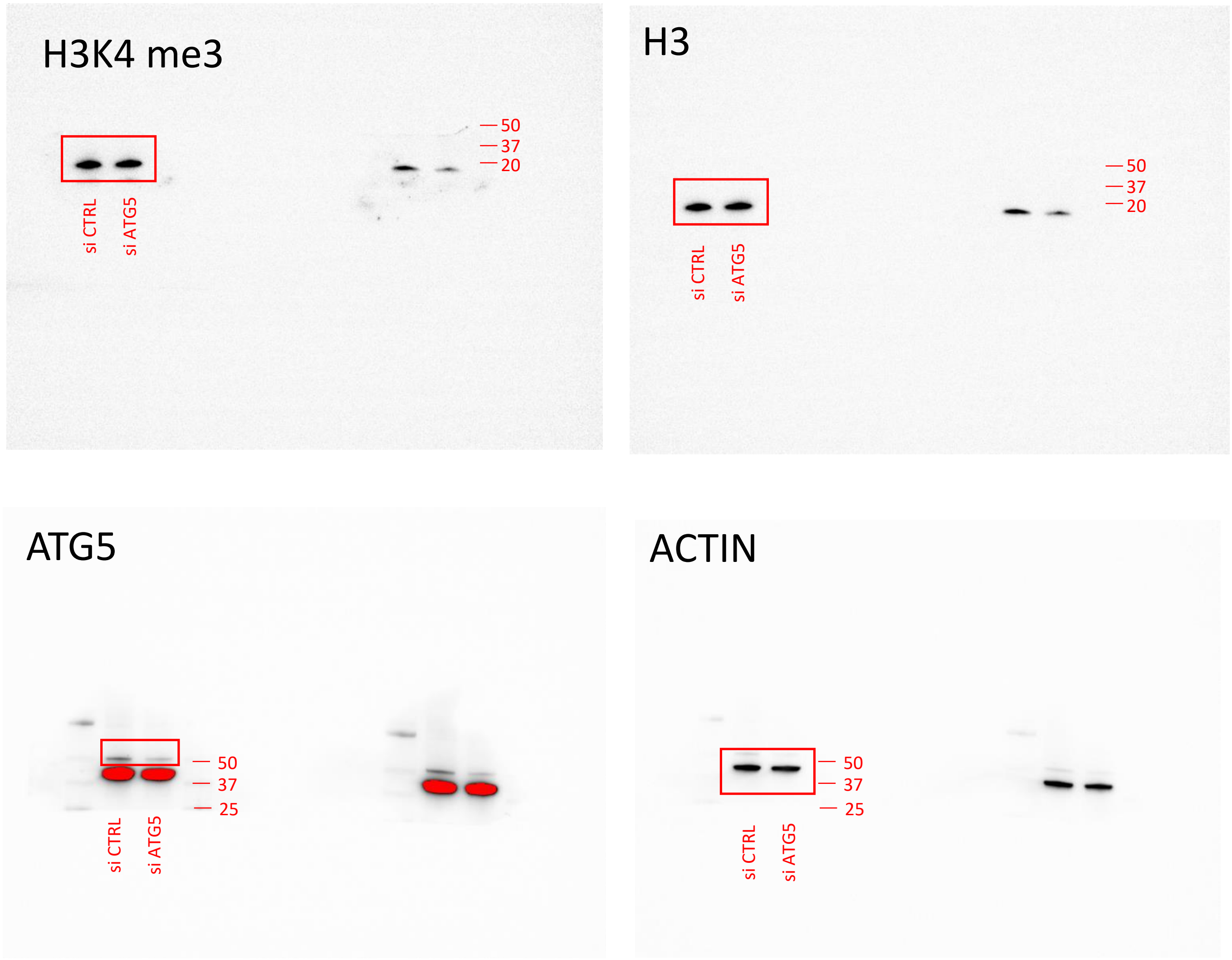

Figure 6D

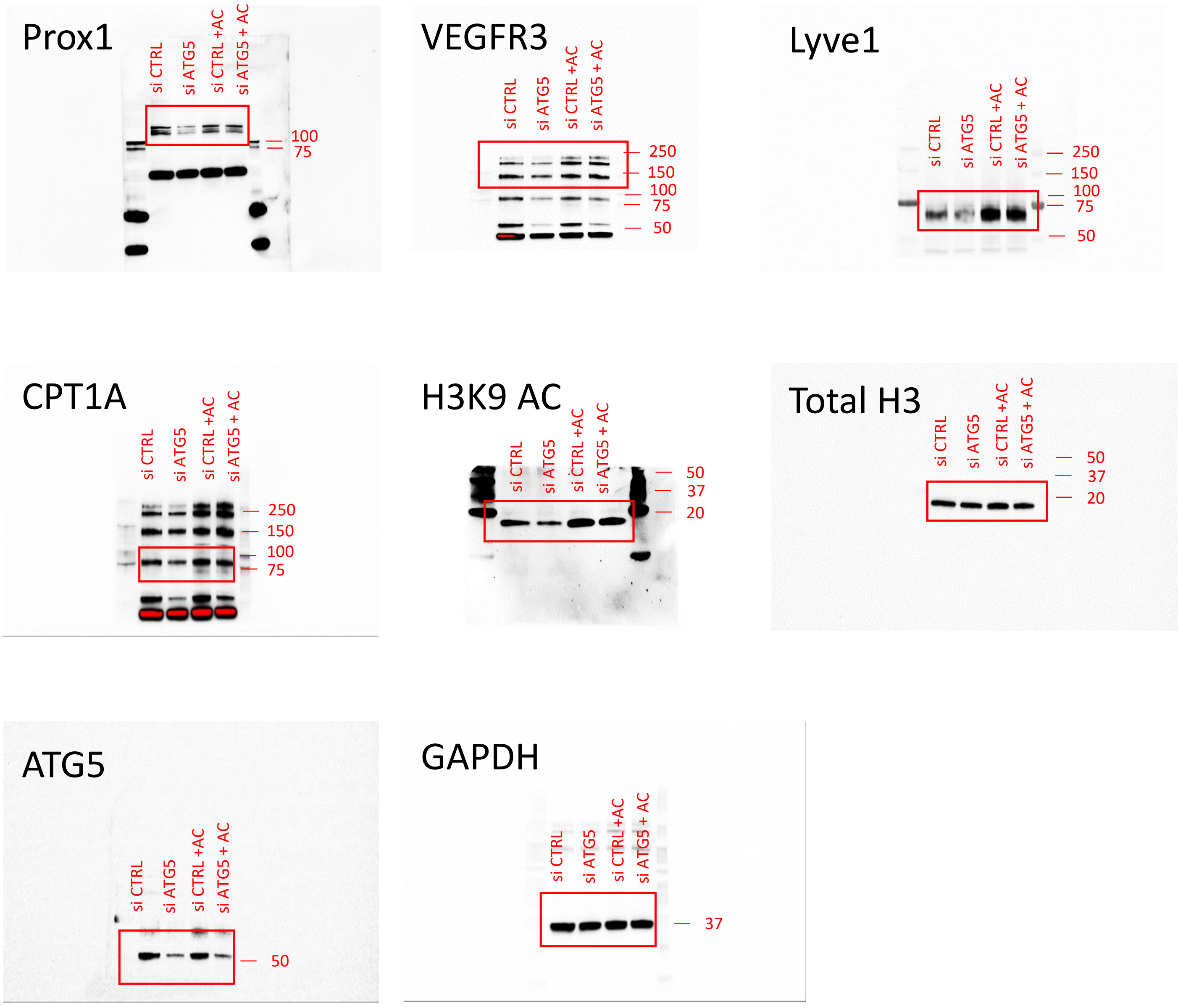
Prox1
si CTRL
si ATG5
si CTRL +AC
si ATG5 + AC
100
75
VEGFR3
si CTRL
si ATG5
si CTRL +AC
si ATG5 + AC
250
150
100
75
50
Lyve1
si CTRL
si ATG5
si CTRL +AC
si ATG5 + AC
250
150
100
75
50
CPT1A
si CTRL
si ATG5
si CTRL +AC
si ATG5 + AC
250
150
100
75
H3K9 AC
si CTRL
si ATG5
si CTRL +AC
si ATG5 + AC
50
37
20
Total H3
si CTRL
si ATG5
si CTRL +AC
si ATG5 + AC
50
37
20
ATG5
si CTRL
si ATG5
si CTRL +AC
si ATG5 + AC
50
GAPDH
si CTRL
si ATG5
si CTRL +AC
si ATG5 + AC
37

Figure 6E

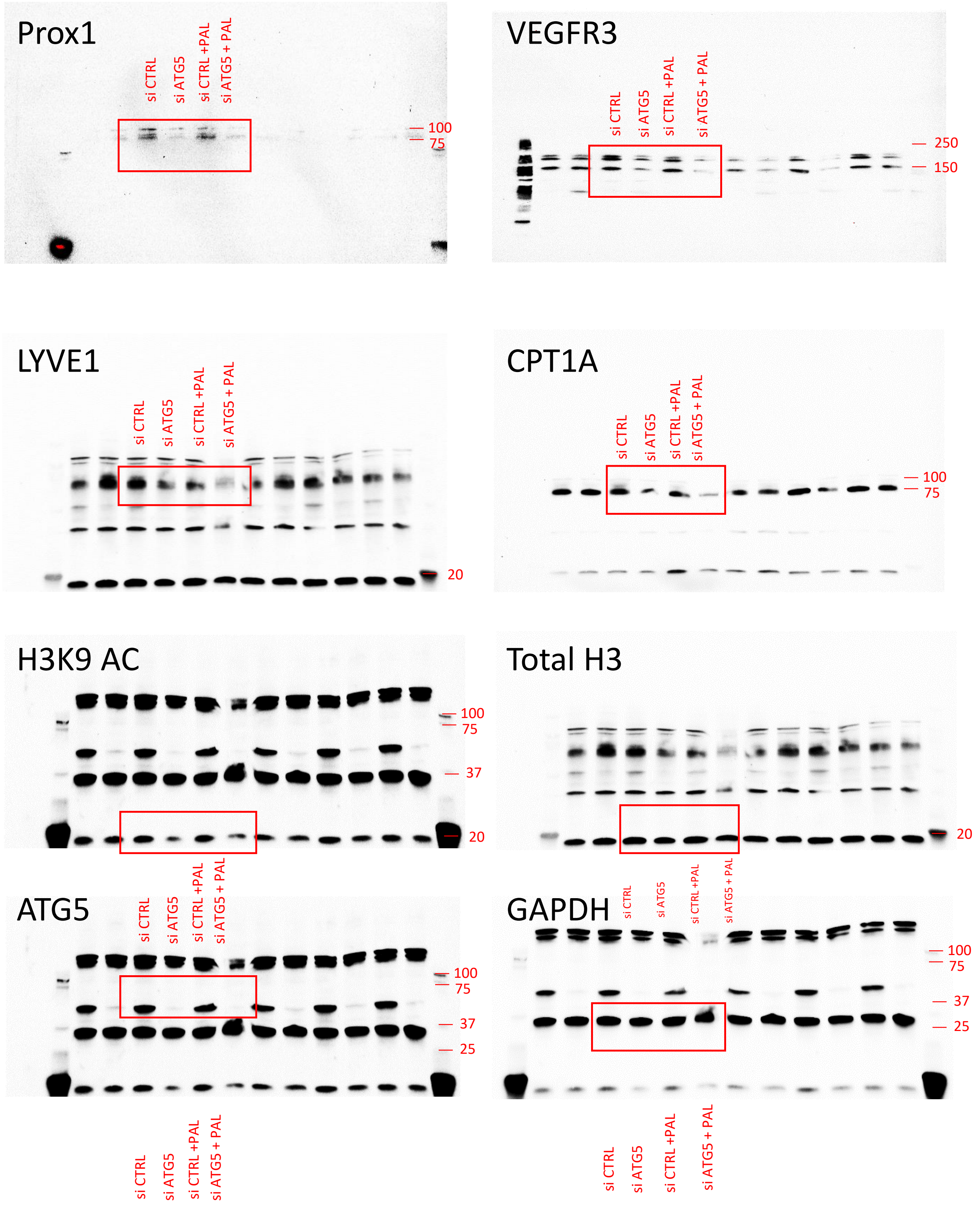

Prox1
si CTRL
si ATG5
si CTRL +PAL
si ATG5 + PAL
100
75
VEGFR3
si CTRL
si ATG5
si CTRL +PAL
si ATG5 + PAL
250
150
LYVE1
si CTRL
si ATG5
si CTRL +PAL
si ATG5 + PAL
20
CPT1A
si CTRL
si ATG5
si CTRL +PAL
si ATG5 + PAL
100
75
H3K9 AC
100
75
37
20
Total H3
20
ATG5
si CTRL
si ATG5
si CTRL +PAL
si ATG5 + PAL
100
75
37
25
GAPDH
si CTRL
si ATG5
si CTRL +PAL
si ATG5 + PAL
100
75
37
25
si CTRL
si ATG5
si CTRL +PAL
si ATG5 + PAL
si CTRL
si ATG5
si CTRL +PAL
si ATG5 + PAL

Supplementary Figure 6A

VEGFR3

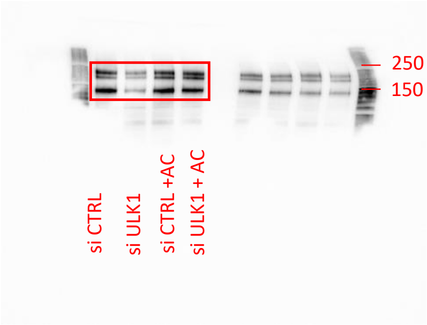

PROX1

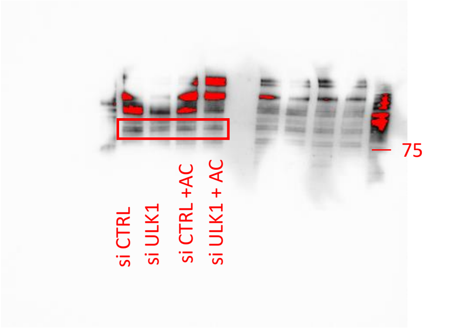

LYVE1

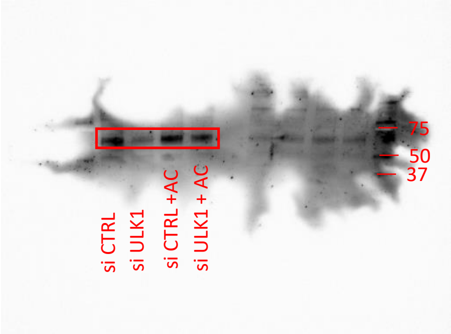

ULK1

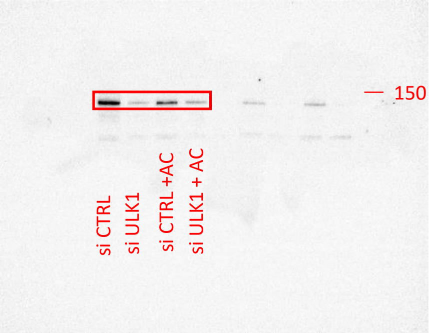

GAPDH

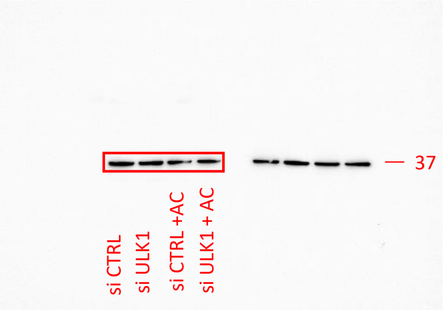

Supplementary Figure 6E

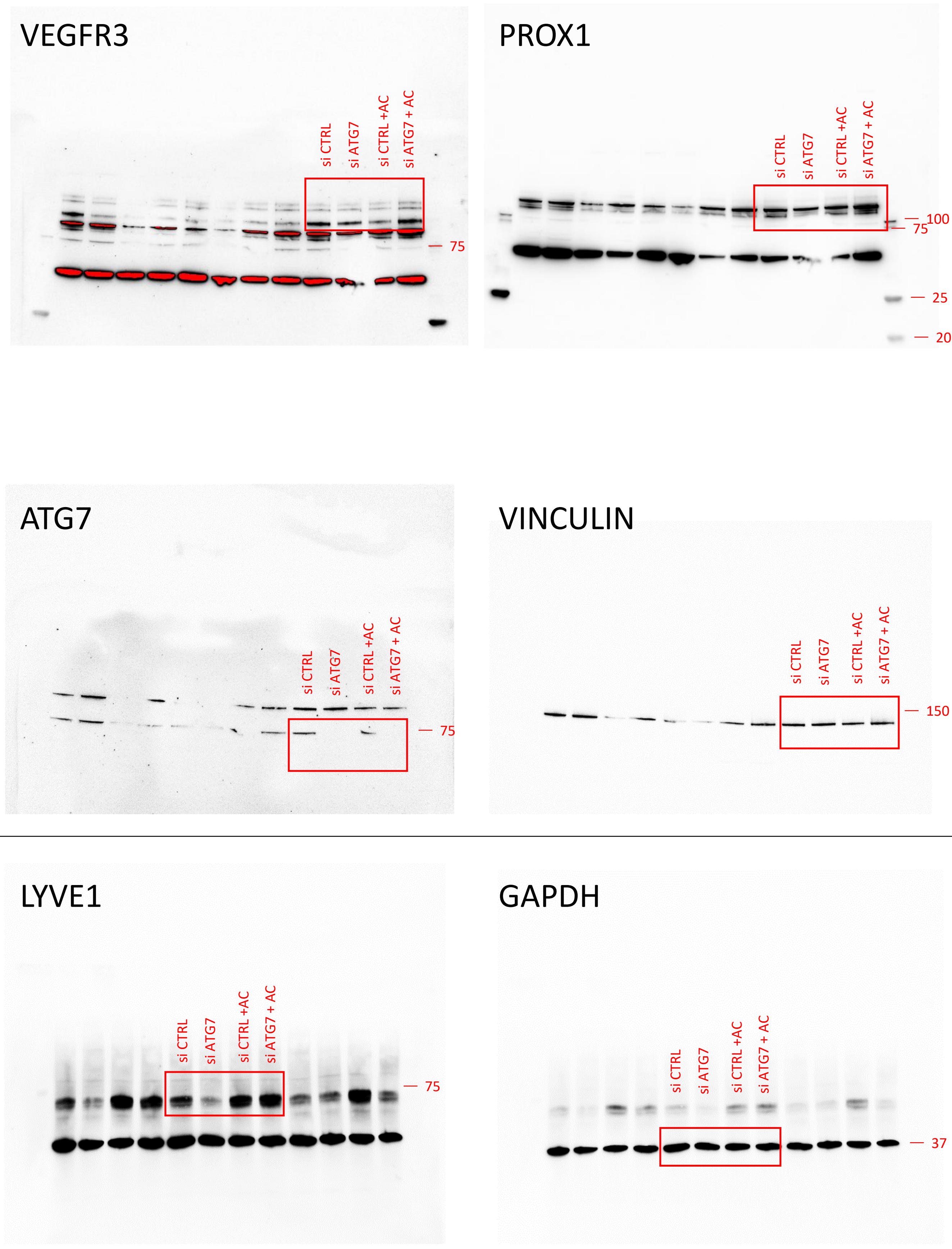

Supplementary Figure 6M

VEGFR3

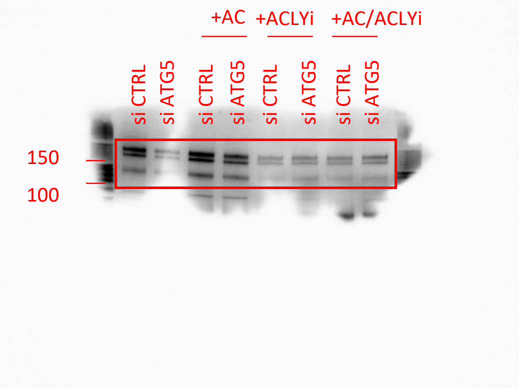

PROX1

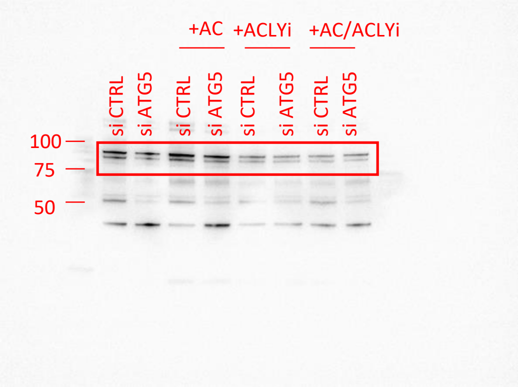

LYVE1

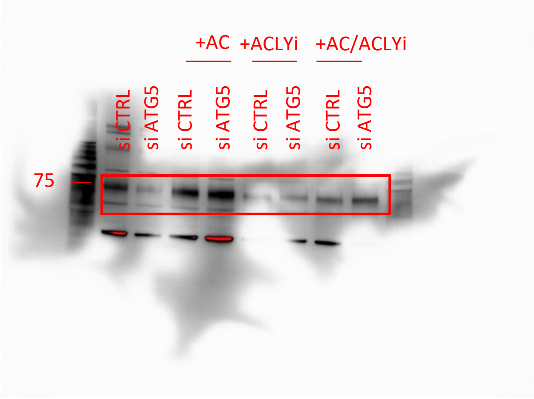

ATG5

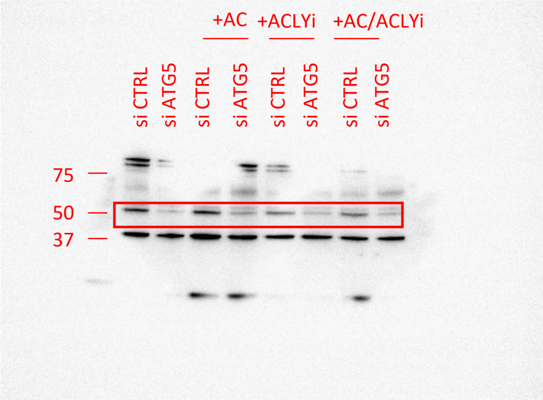

CPT1A

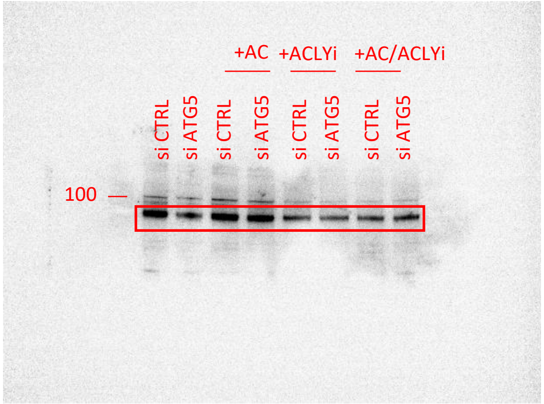

GAPDH

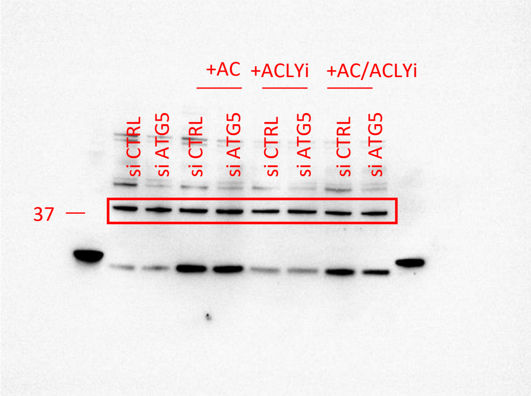

Supplementary Figure 6N

Blot 1 (upper part)

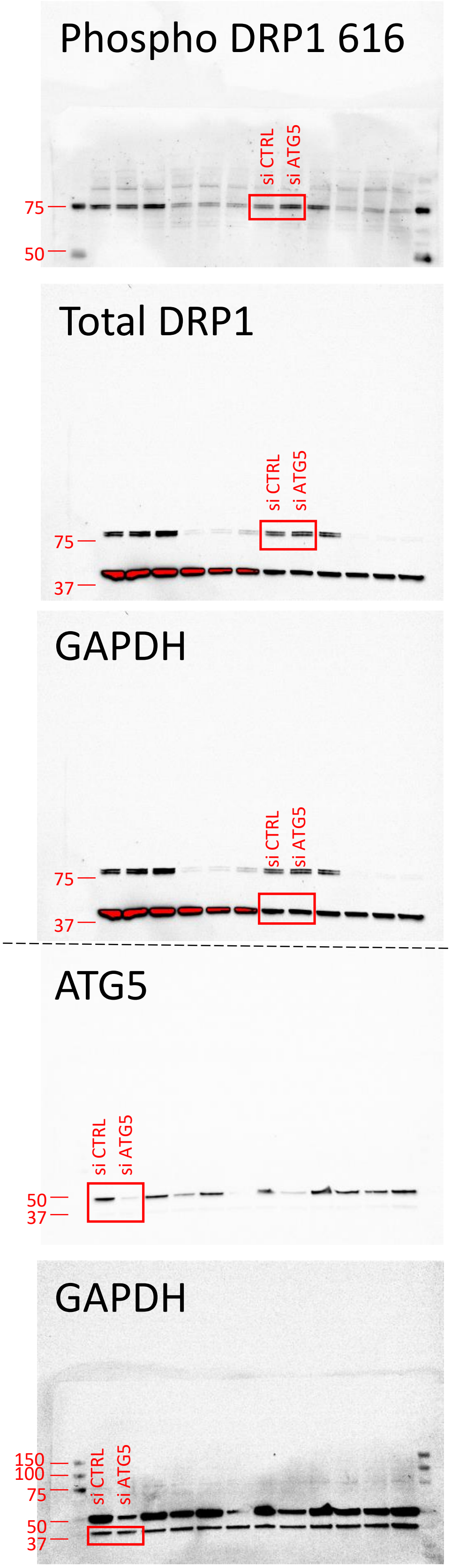

Blot 2 (lower part)

Supplementary Figure 6P

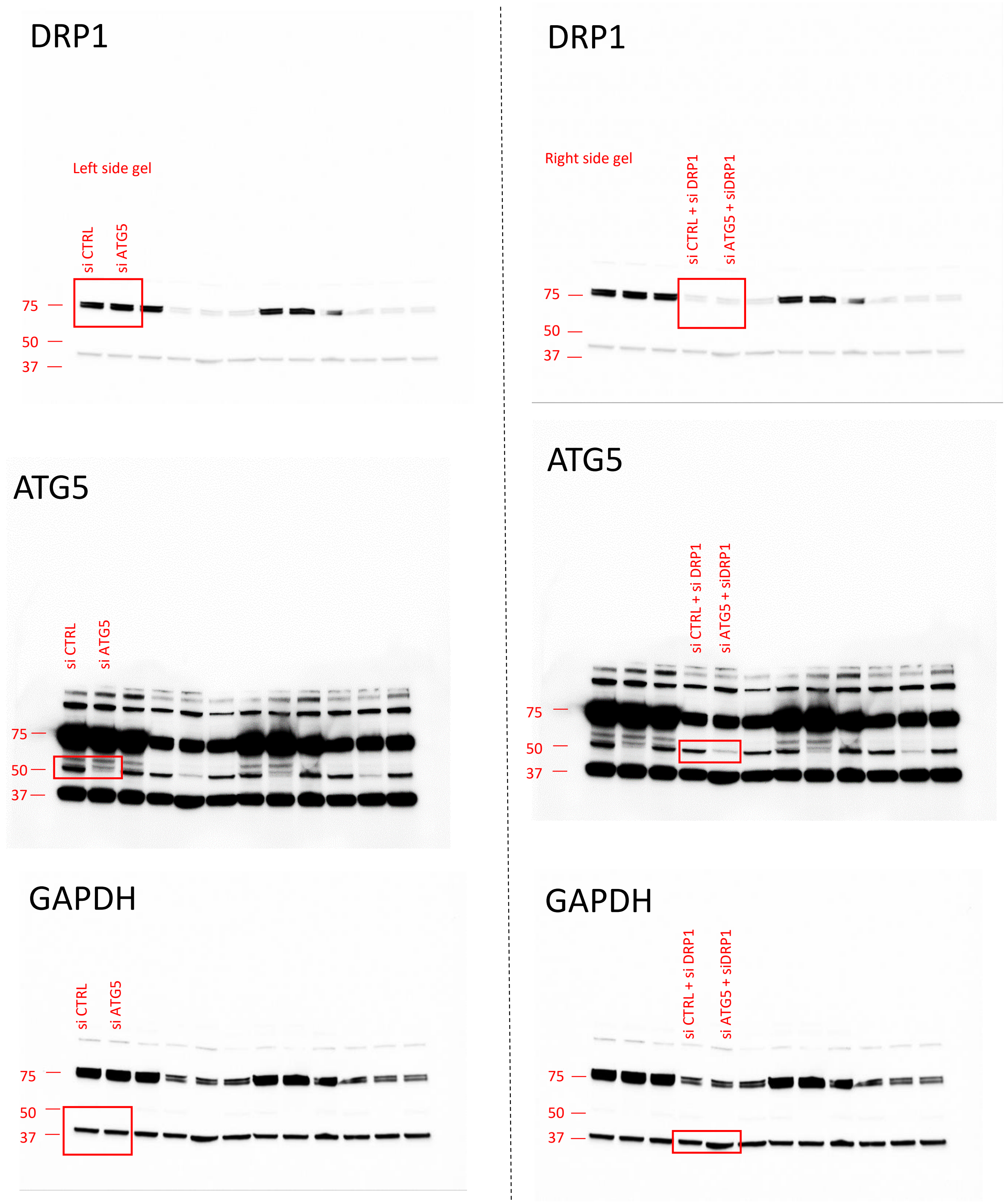

Supplement: Supplementary file 3 — Source Data [file 41467_2022_30490_MOESM3_ESM.zip › Uncropped blots.pdf]
